# Systematic Study of the Multiple Variables Involved in V_2_AlC Acid-Based Etching Processes, a Key Step in MXene Synthesis

**DOI:** 10.1021/acsami.3c01671

**Published:** 2023-05-30

**Authors:** Beatriz Mendoza-Sánchez, Enrique Samperio-Niembro, Oleksandr Dolotko, Thomas Bergfeldt, Christian Kübel, Michael Knapp, Christopher E. Shuck

**Affiliations:** † Institute for Applied Materials—Energy Storage Systems (IAM-ESS), Karlsruhe Institute of Technology, Eggenstein-Leopoldshafen D-76344, Germany; ‡ Helmholtz Institute Ulm for Electrochemical Energy Storage (HIU), Ulm 89081, Germany; § Institute for Applied Materials—Applied Materials Physics (IAM-AWP), Karlsruhe Institute of Technology, Eggenstein-Leopoldshafen D-76344, Germany; ∥ Institute of Nanotechnology and Karlsruhe Nano Micro Facility, Eggenstein-Leopoldshafen D-76344, Germany; ⊥ A. J. Drexel Nanomaterials Institute and Department of Materials Science and Engineering, Drexel University, Philadelphia, Pennsylvania 19104, United States

**Keywords:** V_2_C, MXene synthesis, multivariable, etching, mechanisms, kinetics

## Abstract

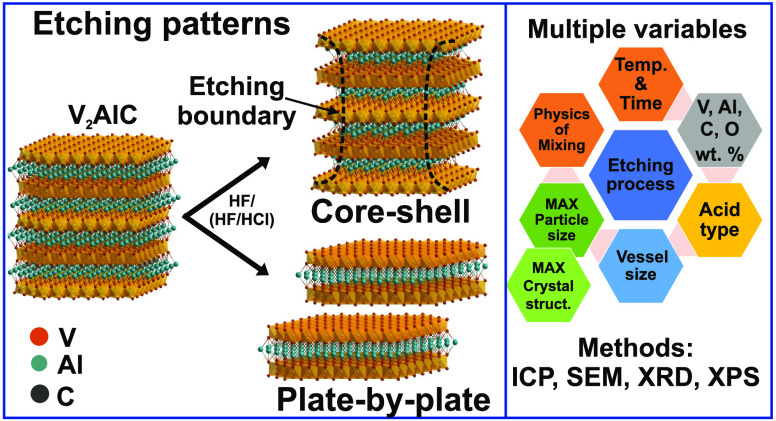

The realization of the broad range of application of MXenes relies on the successful and reproducible synthesis of quality materials of tailored properties. To date, most MXenes have been produced making use of acid-based etching methods, yet an in-depth understanding of etching processes is lacking. Herein, we have engaged in a comprehensive study of the multiple variables involved in the synthesis of V_2_CT_*x*_ with focus on the properties of etched materials. Two main sets of experiments were considered, each using a different V_2_AlC precursor and a range of synthesis variables including reaction time and temperature, mixing rate, and type of acid. Correlations of synthesis conditions–materials properties were investigated using a broad range of characterization techniques including analytical methods, scanning and transmission electron microscopy, X-ray diffraction (XRD), and X-ray photoelectron spectroscopy (XPS). Findings indicated the crucial relevance of properties of the MAX precursor such as elemental composition, particle size, and crystal structure on etching processes and properties of etched materials. Particularly, depending on the MAX precursor, two etching patterns were identified, core–shell and plate-by-plate, the latter describing a more efficient etching. Combined studies of elemental composition, crystal structure, and yield quantification allowed us to evaluate the effectiveness of etching processes. XRD studies revealed key crystal-structure-type of acid correlations showing advantages of using a HF/HCl mix over only HF. Analytical methods XRD and XPS delivered insights into undergoing chemical processes and their influence on bulk and surface chemistry of etched materials. The relevance for reaction kinetics of highly correlated variables such as reaction vessel dimensions, mixing efficiency, and reaction temperature was recognized. For the first time, a MXene synthesis has been investigated comprehensively highlighting its multivariable nature and the high variable intercorrelation, opening up venues for further investigation on MAX and MXene synthesis.

## 1 Introduction

Since their discovery in 2011, MXenes have been utilized for a broad range of applications.^1−3^ MXenes, M_*n*+1_X_*n*_T_*x*_, are derived from M_*n*+1_AX_*n*_ precursors (termed MAX), where *n* = 1–4, M is an early transition metal (Ti, V, Mo, Ta, Nb, etc.), A is an element of group IIIA or IVA (e.g., Al, Ga, Si, Ge, etc.), X is C and/or N, and T_*x*_ describes surface chemical functionalities (-O, -F and -OH, -Cl groups and others).^4,5^ The broad range of properties of MXenes include high electrical conductivity, flexibility of films, in-plane mechanical stiffness, hydrophilicity, which makes them suitable for solution processing, absorption of electromagnetic waves, and others.^2,3,5^ These properties make MXenes suitable for a variety of applications including energy storage, catalysis, electromagnetic interference shielding, sensors, and many more.^2,3^

The realization of these applications depends largely on the physical and chemical properties of MXenes, which are largely determined by the corresponding synthesis procedures. MXenes were first synthesized by etching the A element of the MAX phase using hydrofluoric acid (HF) according to the chemical reaction^1^
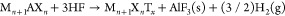
1

In practice, the etching of the A element is partial leading to what is known as multilayer MAX with a M_*n*+1_A_1–*x*_X_*n*_ composition, where *x* is the number of moles of A element that have reacted. Full delamination into a 2D nanomaterial is then achieved by intercalation of a variety of molecules into the multilayer MAX, in some cases followed by the application of mechanical energy.^6,7^ The HF treatment is effective in removing the A element. However, if synthesis conditions are not optimized, HF-based etching procedures can have a deleterious impact on the physical and chemical properties of the resulting MXenes. This includes the presence of crystal defects at basal-planes and edges, especially at high acid concentrations.^8,9^ Alternative methods using a combination of hydrofluoric and hydrochloric acids (HF/HCl), or an *in situ* synthesis of HF, by reacting LiF with HCl, have been demonstrated.^10^ While the latter method produces less defective MXenes, it has only been successful for the delamination of Ti-^10,11^ and some Mo-based MXenes^12^ and, generally leads to low (∼10%) few-layers MXene yields. More recently, methods using molten chloride and fluoride salts have been successful in the synthesis of Ti-based MXenes selectively functionalized with -Cl or -F.^13−15^ Nevertheless, the synthesis based on HF or HF/HCl continues to be the prevailing method to successfully produce most MXenes synthesized to date. Therefore, an in-depth understanding of acid-based etching procedures leading to optimization of yield and properties of MXenes is of critical importance.

Selectively etching the A element of a MAX phase is a crucial step in MXene synthesis. This implies breaking M–A bonds but not M–X bonds. Bond strengths in MAX phases are determined by their elemental composition and stoichiometry. Theoretical calculations, based on force constants as descriptor of bond strength, have led to ranking of the easiness of producing MXenes from a variety of MAX phases.^16^ In general, M–X bonds are stronger than M–A bonds, which should allow for a selective etching of the A element. While these theoretical calculations constitute useful guidance, experimentally, etching processes are complex. Recently, the reaction kinetics of acid-based etching processes of Ti_3_AlC_2_ has been investigated and proposed models offer an insight into the optimization of reaction variables.^17^ However, further understanding of other variables involved in etching processes, evaluation of their impact on properties of resulting MXenes, and studies that involve the many other MXenes of interest for applications are yet to be developed.

Herein, etching processes undergone by V_2_AlC in acids are comprehensively studied. V_2_AlC was selected for this case of study due to known challenges in achieving reproducibility of synthesis and successful delamination. Etching in both HF and HF/HCl was considered. Two main sets of experiments were considered, each using A-V_2_AlC and B-V_2_AlC precursors, synthesized using different synthesis protocols. For each set of experiments, a range of synthesis conditions were considered and included reaction time, temperature, stirring rate, and reaction vessel dimensions. Analytical methods revealed a very different elemental composition of etched materials derived from A and B precursors. The combination of analytical methods, morphology, and XRD studies allowed us to identify two etching patterns, core–shell and plate-by-plate, for etched materials derived from A and B precursors, respectively. The latter described a more efficient etching. These different etching patterns were attributed to different MAX properties that included elemental composition, crystal structure, and particle size. Our studies showed that XRD patterns can be disguising in evaluating etched materials. Only when considered along with elemental composition and yield quantification, we adequately evaluated etched materials as underetched, early etched, well etched, early overetched, and overetched materials. Underetching and overetching were recognized as failure modes of etching processes, and the synthesis variables leading to it were identified. Overetching was correlated to a high degree of V dissolution, and XRD and XPS studies identified the enhanced formation of Al_2_O_3_. Transmission electron microscopy (TEM) and XRD studies confirmed effective etching at edges of well etched materials. Further XRD studies on thermally treated etched materials revealed key differences in crystal structure, depending on the type of acid utilized for etching, showing advantages of using a HF/HCl mix over only HF. Our findings established key correlations between MAX properties and reaction variables with properties of resulting etched materials paving the way for future investigation and development of MXene synthesis methods.

## 2 Experimental Methods

Synthesis procedures are described here. For characterization methods see Supporting Information (section S1).

### 2.1 Chemicals

Hydrofluoric acid HF (48 wt %, 27.58 M), hydrochloric acid HCl (37 wt %, 12.17 M), and tetrabutylammonium hydroxide TBAOH (40 wt % in water) were acquired from Sigma-Aldrich, Germany.

### 2.2 Synthesis of V_2_AlC

#### 2.2.1 Synthesis of A-V_2_AlC, C-V_2_AlC, and D-V_2_AlC

Vanadium powder (99.5%, −325 mesh, Thermo Fisher Scientific), aluminum powder (99.5%, −325 mesh, Thermo Fisher Scientific), and graphite (99%, −325 mesh, Thermo Fisher Scientific) were mixed in a 2:1.3:1 atomic ratio (40 g in total). The precursors were balled milled with 10 mm zirconia balls (2:1 ball:powder ratio) in plastic jars at 60 rpm for 18 h. The powder mixture was then transferred to alumina crucibles, which were placed into a high temperature tube furnace (Carbolite Gero). Ar gas (200 cm^–3^) was continually flown through the furnace for 1 h prior to heating and during the entire annealing procedure. The furnace was heated to 1500 °C at 3.5 °C min^–1^, held for 4 h, then cooled to room temperature at 3.5 °C min^–1^. The resulting solid block product was milled using a drill press with a titanium nitride coated drill. Samples were sieved a first time according to Table S1.

#### 2.2.2 Synthesis of B-V_2_AlC

Vanadium powder (99.5%, −325 mesh, Alfa Aesar), aluminum powder (99.5%, −325 mesh, Alfa Aesar), and graphite (99%, −325 mesh, Alfa Aesar) were mixed in a 2:1.1:0.9 atomic ratio (10 g in total). The precursors were balled milled with 10 mm zirconia balls (2:1 ball:powder ratio) in plastic jars at 50 rpm for 18 h. The powder mixture was then transferred to alumina crucibles, which were placed into a high temperature tube furnace (Carbolite Gero). Ar gas (200 cm^–3^) was continually flown through the furnace for 1 h prior to heating and during the entire annealing procedure. The furnace was heated to 1550 °C at 3 °C min^–1^, held for 2 h, then cooled to room temperature at 3 °C min^–1^. The resulting solid block was milled manually using mortar and pestle. The powders were then washed with HCl 37 wt %; 10 g of powder was added slowly to 20 mL of HCl 37 wt % and stirred overnight. Then the acid was removed by vacuum-assisted filtration while flushing water until the filtered liquid had pH 7. The powders were then dried in vacuum at room temperature.

### 2.3 Synthesis of Etched Materials

Sets A, B, C, and D of etched samples were synthesized using precursors A-V_2_AlC, B-V_2_AlC, C-V_2_AlC, and D-V_2_AlC, respectively. Prior to etching procedures, particle size selection was considered as described in the Supporting Information, section S2.1, Table S1. MAX batches of 10 g were homogenized before sampling for each etching reaction.

In general, 1 g of V_2_AlC powder was added to the acid (20 mL) contained in PTFE reaction vessels in small portions over 20 min. Two types of reaction vessels of different dimensions, L = large and S = small, were used. The reaction mixture was stirred using a magnetic bar. Dimensions of vessels and magnetic bar are reported in the Supporting Information, section S3. A design of experiments for the synthesis of etched materials departed from preliminary experiments where reaction time and temperature were selected based on the sole criteria of etching materials leading or not to delamination (Supporting Information, section S3). For set A, using A-V_2_AlC and etching in HF 48 wt % (20 mL), for most samples, selected reaction time and temperature were 4 days and 45 °C. For set B, using B-V_2_AlC and etching in HF 48 wt % (12 mL)/HCl 37 wt % (8 mL), selected reaction time and temperature were 5 days and 40 °C. Then, a series of samples were synthesized according to the synthesis conditions described in Table 1. Etched samples were named as *P-x-TC-Acid*, where *P* = A, B, C, D indicates the MAX batch, *x* is the sample number, *T*C and *Acid* are the temperature/units (C) and type of acid, respectively, used for etching. For only sample A-5-45C-HF-100rpm, the mixing (stirring) rate was added to the name. Table 1 describes main study samples (used for core chemical analysis, morphology and XRD studies) and complementary samples used for further XRD studies or XPS studies.

**Table 1:** Synthesis Conditions of Samples^a^

Sample	Acid (mL)	Vessel size	*T* (°C)	*t* (d)	MR (rpm)	Yield (%)	Delam.	Deg. etch.
Set A, A-V_2_AlC (1 g)								
Main study samples								
A-1-35C-HF	HF(20)	L	35	4	400	55.84	-	under
A-2-45C-HF	HF(20)	L	45	4	400	25.58	-	over
A-3-55C-HF	HF(20)	L	55	4	400	48.96	-	over
A-4-45C-HFHCl	HF(12)/HCl(8)	L	45	4	400	63.12	-	under
A-5-45C-HF-100rpm	HF(20)	L	45	4	100	60.79	-	under
Samples for XRD								
A-6-40C-HF	HF(20)	L	40	5	400	24.00	no	over
A-7-45C-HF	HF(20)	S	45	4	400	83.96	yes	early etch
A-8-45C-HF	HF(20)	L	45	4	400	64.32	no	under
Set B, B-V_2_AlC (1 g)								
Main study samples								
B-1-40C-HFHCl	HF(12)/HCl(8)	L	40	5	400	80.84	yes	well
B-2-40C-HFHCl	HF(12)/HCl(8)	L	40	5	400	85.80	yes	well
B-3-50C-HFHCl	HF(12)/HCl(8)	L	50	5	400	72.23	yes	well
B-4-55C-HF	HF(20)	L	55	4	400	3.90	-	over
Set C, C-V_2_AlC (1 g)								
Samples for XRD								
C-1-45C-HF	HF(20)	L	45	4	400	60.96	yes	early over
C-2-40C-HF	HF(20)	L	40	5	400	97.48	yes	well
C-3-40C-HF	HF(20)	L	40	5	400	53.80	yes	early over
C-4-40C-HF	HF(20)	L	40	5	400	96.00	yes	well
Set D, D-V_2_AlC (1 g)								
Samples for XPS								
D-1-35C-HF	HF(20)	S	35	4	400	80.84	yes	well
D-2-40C-HF	HF(20)	L	40	4	400	64.1	-	early over
D-3-50C-HF	HF(20)	L	50	4	400	31.9	-	over

a The acids concentration was HF 48 wt % and HCl 37 wt %. Abbreviations: Delam = delamination, Deg. etch. = degree of etching, *T* = temperature, *t* = time, L = large, S = small, MR = mixing rate. In the column of delamination, a dash indicates that no delamination reaction was performed. For delaminated samples, the note “no” or ”yes” makes reference to whether delaminated product was obtained. The notes under = underetched, early etch = early etched, well = well etched, early over = early overetched, and over = overetched make reference to the degree of etching of the sample (see text).

After etching, the reaction mixture was placed in centrifuge tubes (150 mL), DI water was added, and centrifugation was done at 5000 rpm for 10 min. The acidic supernatant was collected with a pipettte and discarded. This step was repeated until the supernatant reached pH 5–6. The etched powder was then further washed and collected by vacuum-assisted filtration, using PVDF membrane filters (0.22 μm pore size). DI water (200–300 mL) was continuously flushed, then the powders were left to settle and dry over the membrane filter (for 3–5 min) prior to collection.

Selected samples underwent delamination (Table 1). Nondelaminated etched powders were weighed and dried using a Büchi glass vacuum oven and a three-step program (room temperature for 3 h, 40 °C for 5 h, and room temperature for 10 h). The yield of etching reactions was calculated as [mass etched material (dry)/mass MAX] × 100 (%). Powders were then processed for characterization or placed in a glovebox (<0.1 ppm of O_2_, <0.1 ppm of H_2_O). Etched powders used for delamination were divided into two fractions. A 20 wt % of the powder was dried and used for yield calculation. The other 80 wt % was used in wet conditions for delamination.

### 2.4 Delamination of Samples

For delamination, the etched powder was added to 20 wt % TBAOH, in a 1.5:1 mol ratio of TBAOH:V_2_C, and stirred at 350 rpm, at 35 °C, for 16 h. Afterward washing steps were carried out to retrieve the TBAOH. The TBAOH intercalated products were placed in centrifuge tubes (150 mL), DI water was added and centrifugation was done at 5000 rpm for 10 min. The top 70–80% volume was pipetted out. This step was repeated until achieving pH 7–8, usually three steps. Then, DI water was added, the product was shaken by hand for 10 min, followed by centrifugation at 3500 rpm for 30 min. The top 70% constituted the delaminated material. This step was repeated twice to obtain more delaminated product. Delaminated films were manufactured using vacuum-assisted filtration.

## 3 Results and Discussion

### 3.1 Studies of A-V_2_AlC and B-V_2_AlC Precursors

The morphology (Figure S1) and particle size (Figure S2) of MAX precursors A-V_2_AlC and B-V_2_AlC, prior to sieving procedures, were studied. The particle size was measured over selected SEM images, and particle size distribution (PSD) was determined (Figure S2). Both samples were constituted by individual particles but also particle aggregates (Figure S1). A-V_2_AlC was constituted of particles of 4–70 μm size with most particles between 10 and 25 μm (Figure S2a). Particles of this sample had a large degree of aggregation (Figure S1b,c) and a large fraction of particles below 1–2 μm (Figure S2b). B-V_2_AlC had a broader PSD as compared to A-V_2_AlC, with particles of 6–160 μm size and with most particles between 15 and 45 μm (Figure S2c). However, the smaller particles were 2–6 μm and their fraction was smaller than in sample A-V_2_AlC (Figure S2d).

The XRD patterns of A-V_2_AlC and B-V_2_AlC were analyzed by Rietveld refinement (RR), and a full description is included in the Supporting Information (section S4). In both samples the main phase was identified as V_2_AlC (*P*6_3_/*mmc* space group (194)). For A-V_2_AlC, three other secondary phases were found: V_4_AlC_3_ (*P*6_3_/*mmc* space group (194)), Al_2_O_3_ (*R*3̅*c* space group, ICSD 9770), and a third one not identified and with only three minor reflections (Figure S3). The weight percentage of phases were V_2_AlC (91.89 ± 0.24 wt %), V_4_AlC_3_ (6.52 ± 0.07 wt %), Al_2_O_3_ (1.59 ± 0.07 wt %) (Table S3). For B-V_2_AlC, only one secondary phase was found: Al_2_O_3_ (*R*3̅*c* space group, ICSD 9770) (Figure S4). The weight percentage of phases were V_2_AlC (97.32 ± 0.37 wt %), Al_2_O_3_ (2.68 ± 0.26 wt %) (Table S4).

Elemental analysis revealed the chemical compositions of A-V_2_AlC and B-V_2_AlC (Table S5). In view of the presence of secondary phases, revealed by XRD, the mass percentage corresponding to only the V_2_AlC phase was taken into account to calculate mol composition. The 2 × (mol i/mol V) ratio, where i = V, Al, C, O, was 2 ± 0.02, 0.96 ± 0.007, 1.00 ± 0.008, 0.09 ± 0.007 for A-V_2_AlC and 2 ± 0.02, 0.91 ± 0.007, 1.00 ± 0.008, 0.09 ± 0.0007 for B-V_2_AlC. This is a mol composition close to the theoretically expected but with a deficiency in Al, slightly more significant for B-V_2_AlC. A slight content of O was detected, which is linked to the presence of Al_2_O_3_ revealed by XRD studies and surface oxidation of V_2_AlC particles confirmed by XPS studies (section 3.10). For practical purposes, the MAX precursors continued to be named using the theoretical stoichiometry.

### 3.2 Elemental Analysis of Etched Materials Using Analytical Methods

MAX particle classification was considered prior to etching procedures (section S2.1, Table S1). Only the fraction of a particle size <36 μm was utilized. In addition, MAX powder homogenization was considered prior to etching procedures.

The elemental composition of etched samples of sets A and B was investigated using analytical methods that included inductively coupled optical emission spectroscopy (details of methods are given in section S1.2). Table 1 summarizes the synthesis conditions for set A and set B. Only A-(1-5)-TC-Acid samples of set A and B-(1-3)-TC-Acid samples of set B were considered for chemical analysis.

**Figure 1:**
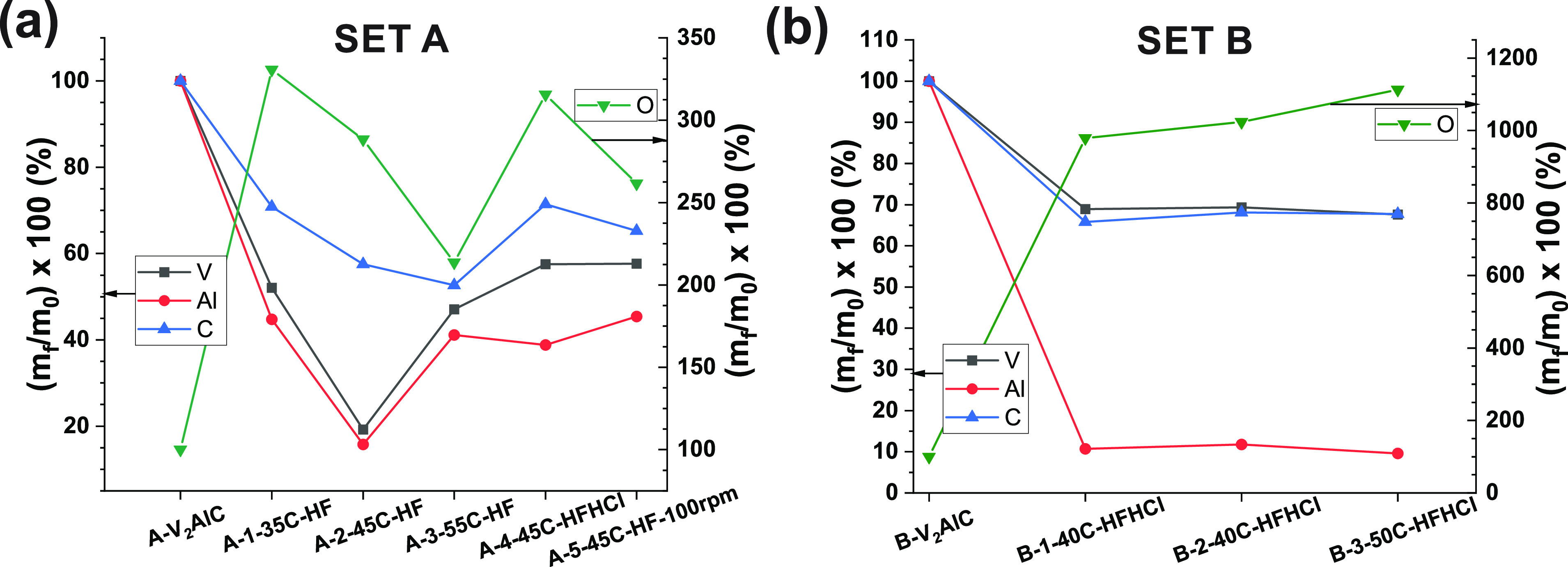
Element analysis of samples of (a) set A and (b) set B. Here, % *m*_*f*,*n*_ = (*m*_*f*,*n*_/*m*_*i*,*n*_) × 100, where *m*_*f*,*n*_ is the mass of an element *n* in an etched sample and *m*_*i*,*n*_ is the initial mass of element *n* in the corresponding precursor. The determination error is <0.5 wt % for Al, <1.0 wt % for C, <1.5 wt % for V, and <2–3 wt % for O.

Figure 1 shows the mass percentage quantification of elements for samples of sets A and B. Notice that this analysis applies to the total sample composed of different phases. Nevertheless, since the V_2_AlC phase is the main phase (>91.89 ± 0.24), findings apply mainly to it. For set A, it is clear that Al is indeed being etched away, but in parallel, V also gets etched away. For most samples, 42.5–53 wt % V was lost. Only in the sample A-2-45C-HF, the mass loss was very drastic, 80.8 wt % (Figure 1a). Similarly, for most samples, 54–61 wt % Al was etched away except for a drastic mass loss of 84.3 wt % for sample A-2-45C-HF, i.e., in pretty much the same proportion as V. Then, 28–47 wt % C was also lost for all samples.

In set B, again all three elements are lost during etching procedures. However, unlike in the case of set A, 88–91 wt % Al was preferentially etched away over only 30–32 wt % V. C was etched away in a similar proportion to V (31–34 wt %). Here, the V and Al composition is clearly more reproducible across samples. Consideration of mol of elements per mol of C allows a clearer visualization of the variations of V and Al versus the precursor MAX (section S6.1, Figure S5). Determined O and F content for sets A and B is discussed in the Supporting Information (section S6.2, Figure S6).

### 3.3 Discussion of the Phenomenon of V Dissolution

The mass loss of V during etching of V_2_AlC in HF or HF/HCl is experimentally well-known and gives a characteristic green color to supernatants during postwashing steps of etched products (Figures S7 and S8). Theoretical calculations have reported the force constants around M, A, and X atoms, which are an indicator of bonding strength of a particular atom within the MAX structure.^16^ For V_2_AlC, the force constants for V, Al, and C atoms are 51.94 eV/Å^2^, 21.85 eV/Å^2^, and 59.16 eV/Å^2^, respectively.^16^ Thus, Al atoms are expected to be more weakly bonded to surrounding atoms as compared to V and C. Thus, Al should be preferentially etched away by acids. However, the force constant for V–V bonds, in the same layer, is 1.91 eV/Å^2^ < 2.05 eV/Å^2^ for V–Al atoms.^16^ Thus, at a MXene surface, V–V bonds are more vulnerable to reaction with an acid than the V–Al bonds. This would leave dangling V atoms in the V–C (force constant = 8.35 eV/Å^2^) structure of the MXene, which upon further acid attack are dissolved. Previous studies report “carbonization” (presumably implying V reaction) of V_2_AlC pillars after only 6–15 h, at the edge and 10 μm from the edge of pillars.^18^ Thus, for the 4–5 days of reaction time here used, HF is expected to dissolve V atoms. On the other hand, the presence of crystal defects such as atom vacancies may contribute to destabilization of the crystal, especially when etched at high acid concentrations.^8^ Recent findings report the presence of O in the C layer of some MAX and MXenes, which may contribute to destabilization of crystal structure.^19,20^

The green color of V in dissolution indicates the presence of V^3+^ ions.^21^ According to standard reduction potentials *E*°,^21^ in acidic media, V^2+^ (violet) ions will oxidize to V^3+^ (green), (V^3+^ to V^2+^*E*° = −0.26 V). If present, V^5+^ (yellow) would reduce to V^4+^ (V^5+^ to V^4+^*E*° = +1 V), and V^4+^ would reduce to V^3+^ (V^4+^ to V^3+^*E*° = +0.337 V). Therefore, in acidic media, only V^3+^ can exist. XAS studies of V_2_C showed oxidation sates below +2.^22^ Thus, the oxidation state of V in the MAX phase would be expected to be even lower as per the presence of Al. Therefore, we can conclude that upon etching, V in an oxidation state below +2 gets oxidized to V^3+^.

### 3.4 Discussion of Variables Playing a Role in Etching Processes

In the following, reaction variables are discussed, where the terms underetching and overetching are utilized. These terms make reference to the degree of etching of a sample implying several chemical and material properties. Such properties are investigated here, and thus, their definition is further specified as the text advances (section 3.6). In general, underetching makes reference to a sample where the removal of A atoms has been too poor so that not even a minimum delamination can be obtained. Conversely, overetching makes reference to a sample where etching has advanced to a high degree that has caused major undesirable changes on material properties including a high loss of material. Other terms making reference to degree of etching are introduced throughout the text.

#### 3.4.1 Reaction Time

Samples of set A, in general, needed a shorter etching reaction time (4 days) than samples of set B (5 days) to achieve materials that led to delamination (see section S3, Table S2). This is explained by the submicrometer particle size fraction present in A-V_2_AlC (Figure S2b) but not in B-V_2_AlC (Figure S2d). Some samples of set A had a very low reaction yield, e.g., sample A-2-45C-HF (Table 1). Similarly, this was correlated to a large fraction of particles of submicrometer size, which underwent overetching. This was, in turn, correlated with a high degree of V and Al dissolution (Figure 1a).

#### 3.4.2 Reaction Temperature

The V mass loss was larger for sample A-3-55C-HF (53 wt %) than for sample A-1-35C-HF (48 wt %), which indicated an enhanced etching with a 10 °C temperature increase (Figure 1a). However, the mass loss for a reaction at 45 °C was much larger for A-2-45C-HF (80.8 wt %), which indicated that other variables played a role in enhancing etching for this sample, including mixing-related aspects (see section 3.7). When using HF/HCl instead of HF, the mass loss was much lower for sample A-4-45C-HFHCl (42.5 wt %) than for A-2-45C-HF (80.8 wt %), indicating that HF/HCl decreased V-dissolution. The same observations apply for Al, as the quantities of this element and V were highly correlated for samples of set A. For samples of set B, there was a slightly larger V mass loss for sample B-3-50C-HFHCl (32.4 wt %) than for sample B-2-40C-HFHCl (30.7 wt %) (Figure 1b). Correlated variables having an impact on etching processes, and thus degree of dissolution of V and Al, include particle PSD and morphology (discussed in section 3.5).

#### 3.4.3 Nominal Mixing Rate

For set A, all the samples were mixed (stirred with a magnetic bar) at a nominal rate of 400 rpm except for A-5-45C-HF-100rpm, which was mixed at 100 rpm. This resulted in a slightly lower V mass loss as compared to the other samples but not for all, e.g., A-1-35C-HF and A-4-45C-HFHCl. Therefore, the nominal mixing rate had no clear correlation with V dissolution. Mixing issues, other than the nominal mixing rate, are further discussed in section 3.7.

#### 3.4.4 MAX Properties

A striking difference across set A vs set B is the very different proportion of elements lost during the etching reaction (Figure 1). In set A, the correlation of loss of V and Al is clear, whereas in set B, V is rather lost in a similar proportion to C while Al is the element preferentially etched away. This indicated that differences in etching results are in a high degree rooted on properties of the V_2_AlC precursor. First, particle size and PSD have already been highlighted as playing a role in reaction time and degree of V/Al dissolution. A second variable is chemical composition. Here, A-V_2_AlC and B-V_2_AlC were synthesized using different V:Al:C mol ratios, which had an impact on resulting MAX elemental composition (Table S5), presence of secondary phases, and presence of defects (see discussion in section 3.7). Analytical methods reported a slightly larger deficiency of Al in B-V_2_AlC than in A-V_2_AlC (0.91 < 0.96), which most likely had an influence in bond strength and, thus, etching processes. Third, the presence of the secondary phase V_4_AlC_3_ in A-V_2_AlC, but not in B-V_2_AlC, in a significant mass percentage (6.52 ± 0.07 wt %) most likely affected the crystal growth and, thus, etching processes. We know that V_4_AlC_3_ requires longer times of reactions (7 days) and at least 45 °C to etch away the Al in HF/HCl. Interspersion of this secondary phase with particles of the main phase would lead to different etching reaction times. Morphology and XRD studies, addressed next, revealed further details of etching processes.

### 3.5 Morphology Studies of Etched Materials

**Figure 2:**
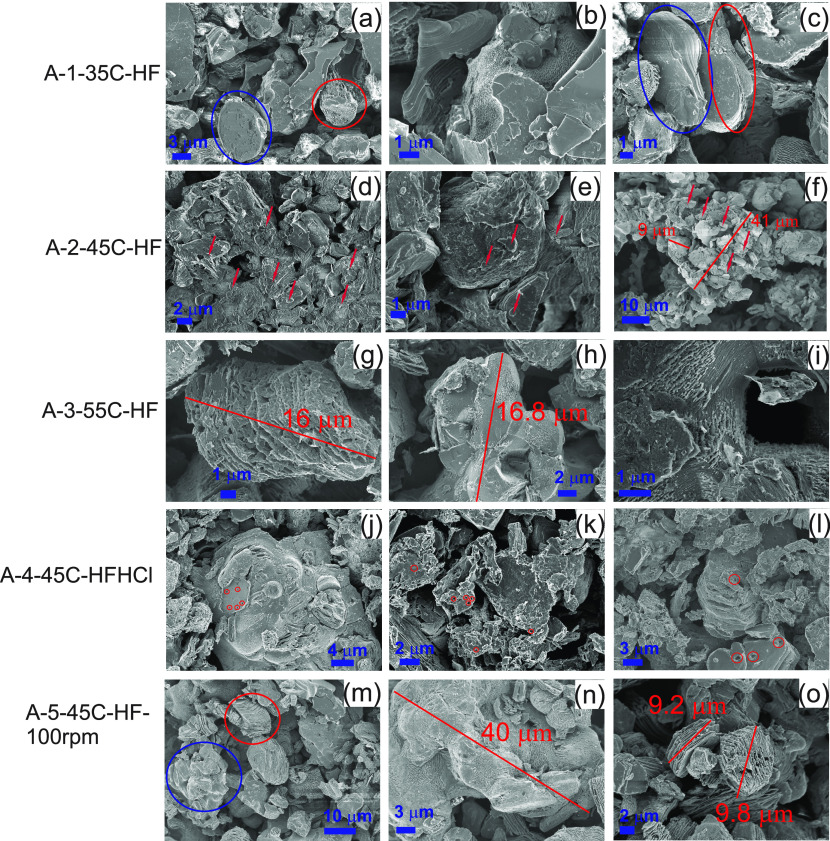
SEM images of samples: (a–c) A-1-35C-HF, (d–f) A-2-45C-HF, (g, h) A-3-55C-HF, (j–l) A-4-45C-HFHC,l and (m–o) A-5-45C-HF-100rpm. Red circles indicate etched particles, blue circles indicate poorly etched or nonetched particles, and arrows indicate very small particles. Very small red circles indicate pores of size < 0.5 μm.

**Figure 3:**
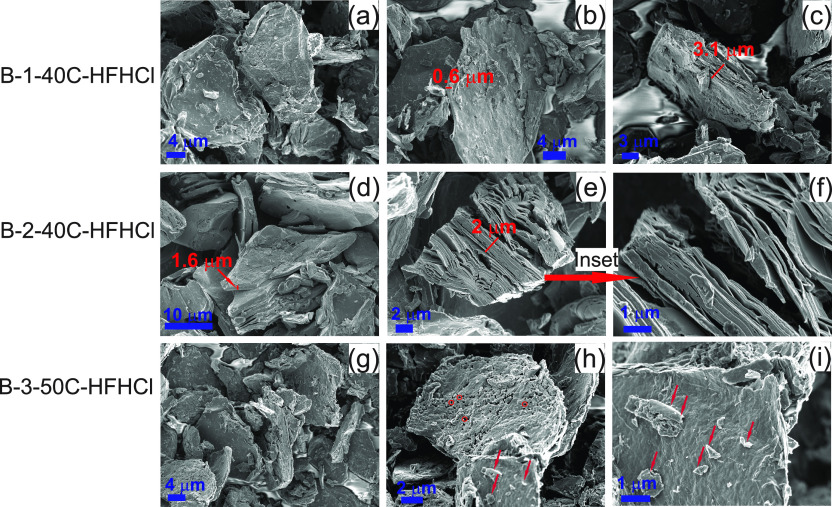
SEM images of samples: (a–c) B-1-40C-HFHCl, (d–f) B-2-40C-HFHCl, and (g–i) B-3-50C-HFHCl. Arrows indicate very small particles. Very small red circles indicate pores of size < 0.5 μm.

The morphology of etched samples of set A and set B was studied (Figures 2 and 3). Etching induced morphological features that departed from the morphology of the starting MAX phase. These changes included an amorphous morphology at the top of particles clearly lacking the integrity of a MAX crystal (as in Figure 2e), layers sticking out of particles (as in Figure 2i), “galleries” in crystals (as in Figure 2g), and porosity (as in Figure 2k). In the following, etching refers to these morphological changes and etching degree refers to the visible extent of these morphological changes.

For samples of set A, etching was heterogeneous within a particle and across particles. For the A-1-35C-HF sample, particles were barely etched at the surface (Figure 2a,b). Just besides these large particle aggregates, some particles were etched, at the surface, and some others did not (Figure 2a,c). This heterogeneous etching was attributed partly to the low temperature used of 35 °C, below the optimized temperature of 45 °C (see section S3). A second factor could be an inefficient mixing, other than the nominal mixing rate, which includes an uneven mixing across the reaction vessel (see section 3.7). Third, a condition contributing to poor etching could be the disintegration of large particle aggregates into constituent particles at a late stage of the reaction, which were then etched for a shorter time than the rest of the particles. Supporting this view, previous studies on etching of Ti_3_AlC_2_ in HF reported disintegration of particle aggregates during a 24 h reaction time.^9^

For the A-2-45C-HF sample, the top surface of particles showed an amorphous top layer (Figure 2d–f). The mass composition of this sample was V 19.22 ± 1.11 wt %, Al 15.73 ± 0.3 wt %, and C 57.52 ± 1.66 wt %. Due to an enhanced etching at the surface, the C mass percentage was, most likely, even higher, constituting an amorphous carbon top layer. Supporting this view, previous Raman studies proved the presence of amorphous carbon at the surface of V_2_AlC particles, formed as a result of overetching.^23^ Their top surface morphology was very similar to the morphology of our samples. Furthermore, unlike other samples of set A, this sample showed charging effects during imaging, which described the poor electrical conductivity of particles covered with carbon. In addition, this sample had many submicrometer size particles with an amorphous morphology, either lying on top of large particles (marked by arrows in Figure 2d–f) or forming aggregates (Figure 2f). Due to the submicrometer particles size, overetching is expected, which contributed to the large loss of V and Al and to the low yield obtained (Table 1).

For the A-3-55C-HF sample, the higher temperature of 55 °C, as compared to the other samples, indeed induced a deeper etching within an entire particle showing “galleries” left by etched away Al (Figure 2g). However, some aggregates of similar size remained unetched (Figure 2h). The temperature indeed induced a better etching within a particle, but this effect was not homogeneous across particles and particle aggregates. Thus, it is clear that the particle size polydispersity prevents a true reaction temperature optimization. Reaction temperature can be optimized for a given particle size *p*_*m*_. Particles of size <*p*_*m*_ will undergo overetching, and particles of size >*p*_*m*_ will undergo underetching. Another intriguing fact is that some particles of these samples showed tunnel structures upon etching, e.g. Figure 2i. This is particularly true for samples derived of this particular precursor A-V_2_AlC. This might have been produced by a leaving crystal particle of either MAX or a secondary phase (elucidated by XRD studies (section 3.1)) upon etching.

The sample A-4-45C-HFHCl showed an interesting particularity, the presence of pores of size < 0.5 μm at the surface (Figure 2j) and within (Figure 2k,l) particles. This seemed to be induced by etching with the combination of HF/HCl acids. The same phenomenon was observed for the samples of set B etched with a HF/HCl mix.

For the A-5-45C-HF-100rpm sample, the poorer mixing at 100 rpm, instead of 400 rpm used for the other samples, seemed to have induced a more localized etching at the top surface, observed for both large particle aggregates (Figure 2n) and small particles (Figure 2o). However, as in the other samples, the nonhomogeneous etching across particles depending on size was evident and surely enhanced (Figure 2m,o). XPS studies revealed that the slow mixing rate used in this sample favored the formation of vanadium oxides (section 3.10).

**Figure 4:**
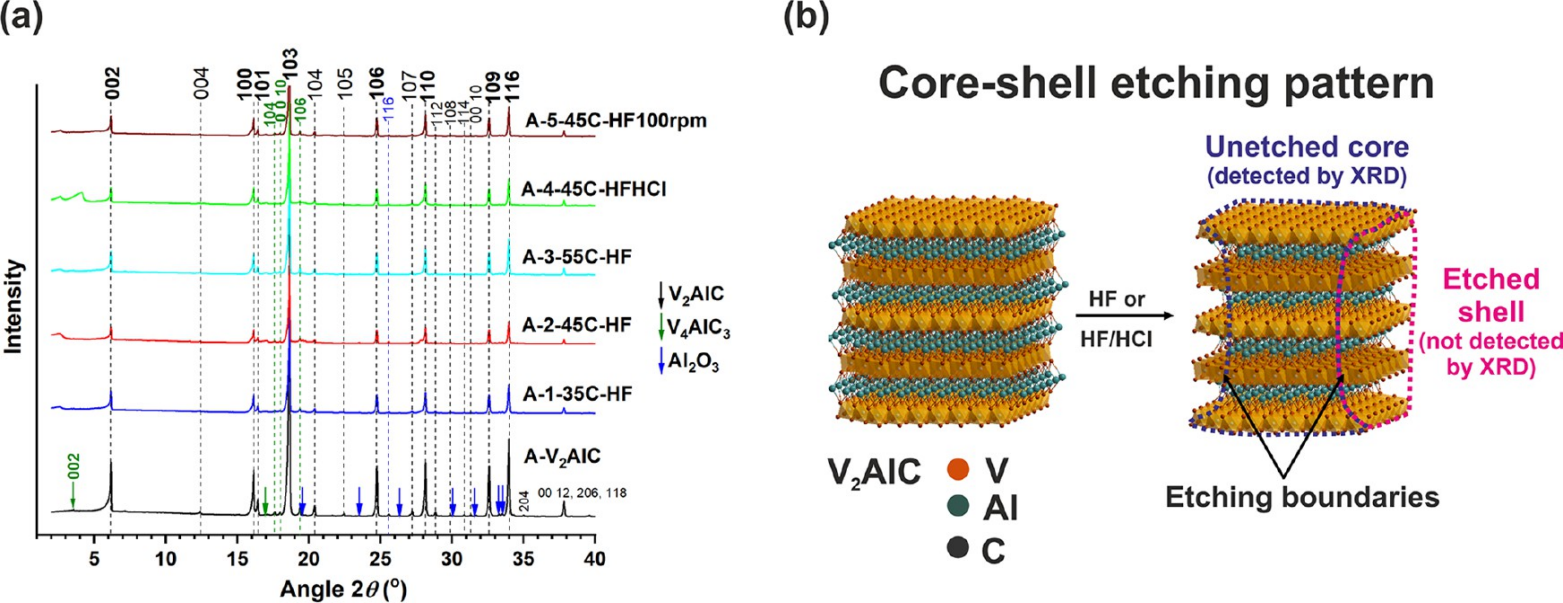
(a) XRD patterns of samples of set A: A-1-35C-HF, A-2-45C-HF, A-3-55C-HF, A-4-45C-HFHCl, and A-5-45C-HF-100rpm. The main reflections of the V_2_AlC phase are indicated by labels in bold font. (b) Schematic of the core-shell etching pattern followed by samples of set A.

In view of these results for set A, a “core–shell” type pattern, where the shell was etched first and then it advanced toward the core of the particle, was proposed (Figure 4b). XRD studies described the unetched crystalline cores of samples of set A (section 3.6). This etching pattern is expected as per the solid–liquid nature of etching processes and was enhanced for samples A-1-35C-HF (Figure 2b) and A-2-45C-HF (Figure 2d–f). Similar to this work, previous findings reported etching advancing from particle surface to core for Ti_3_AlC_2_ synthesized in HF and HF/HCl.^9^

The effect of particle size on efficiency of etching processes was evident on all samples of this set (Figure S10a–e). Submicrometer and few micrometers size particles were overetched, whereas larger particles remained unetched. In large particle aggregates etching occurred on small constituent particles, whereas large constituent particles did not get etched (Figure 2j). Moreover, in a large particle aggregate, etching seemed to follow preferential planes, for instance Figure 2b. This may also be linked to interspersion of secondary phases within particles and particles aggregates of the main phase, which then had different etching rates.

Etching process of set B proceeded in a very different manner than in set A (Figure 3). First, the sieving procedure of the B-V_2_AlC powder selected well the particles below 36 μm. Large particle aggregates were rarely observed in the etched samples (Figure S10f–h). Second, the etching was more homogeneous across individual particles, as per the constituent “plate” crystal units breaking apart, e.g., in Figure 3e,f. These plates constituted by various “subplate” crystal units, when further breaking apart, seemed to have given origin to “platelets” of microsized thickness (Figures 3a,b,d) of 0.6 μm (Figures 3b). Here, we identify this as a “plate-by-plate” etching pattern (Figure 7b), very different from the core–shell etching pattern followed by samples of set A. Then, the etching was uniform across different particles in the sample, as “platelets” were mostly observed, with rare appearance of integral/uneteched particles (Figure S10f–h). In the sample B-3-50C-HFHCl, the top surface of the platelets was populated by tiny particles, most likely being the remains of overetched small particles (Figure 3h,i). In addition, and just as sample A-4-45C-HFHCl of set A, samples in set B showed pores of size < 0.5 μm on the flakes surface (e.g., Figures 3h). Again, this was correlated to etching procedures in HF/HCl.

In general and in agreement with the mass percentages reported by the analytical methods (Figure 1), this plate-by-plate etching pattern was consistent with a more efficient etching of Al and decreased dissolution of V, getting closer to an ideal etching procedure. These microsized platelets are the ones that get then fully delaminated into MXenes upon delamination processes. Delamination procedures were followed for samples of set B, and the delamination was successful for all of them (Figure S9).

### 3.6 X-ray Diffraction Studies of Etched Samples

**Figure 5:**
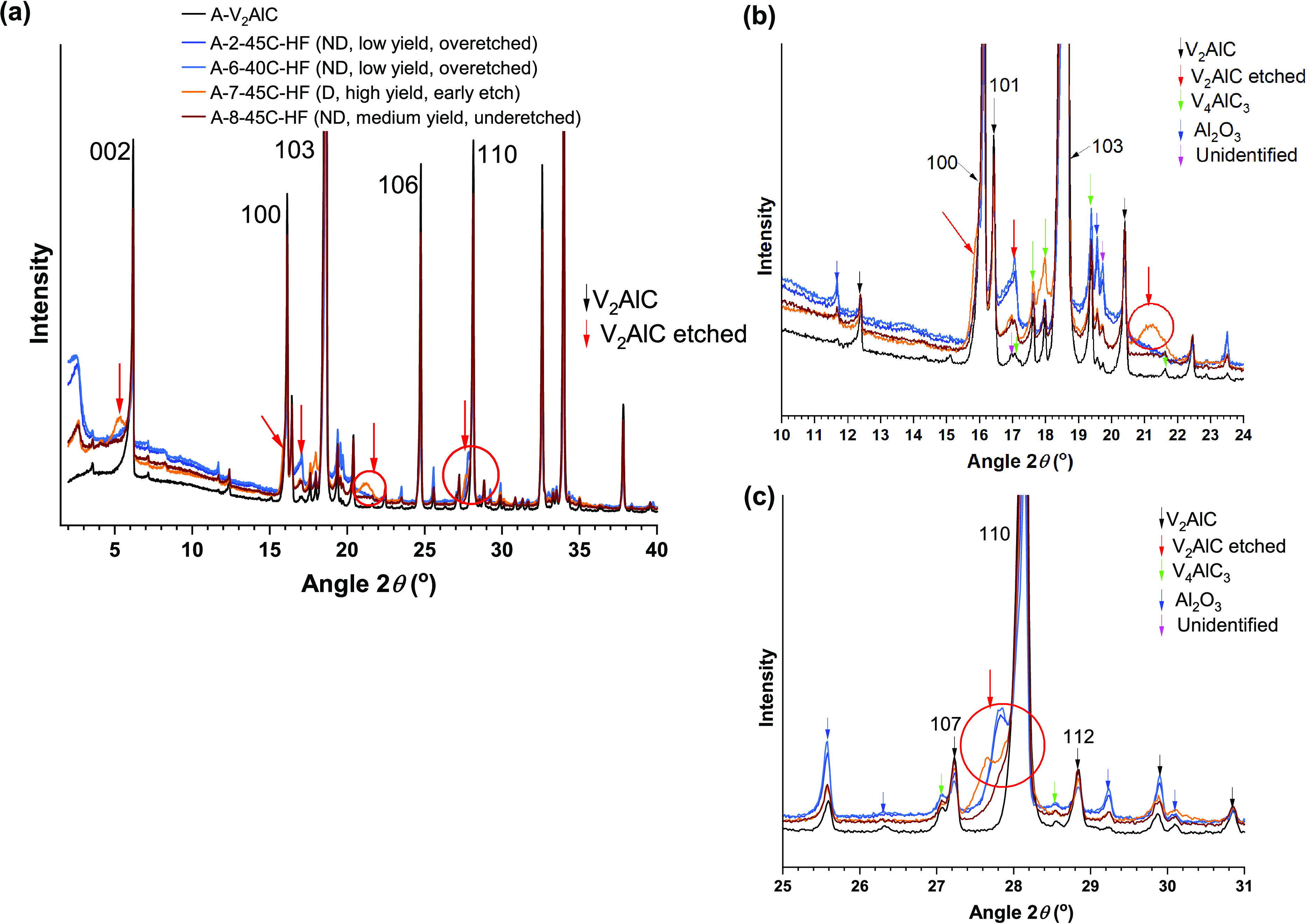
(a) XRD patterns of samples of set A: A-2-45C-HF (overeteched), A-6-40C-HF (overetched), A-7-45C-HF (early etching), and A-8-45C-HF (underetched). (b)–(c) are enlarged views at specific 2θ ranges. Emerging features of the sample A-7-45C-HF are indicated with red circles and red arrows. The XRD pattern of the precursor A-V_2_AlC is included for reference. ND indicates no delamination, and D indicates delamination. The rest of the legend makes reference to the yield (Table 1).

XRD studies of samples of sets A and B were performed. Interestingly, the XRD patterns of all samples of set A were very similar to each other and to the XRD pattern of the precursor A-V_2_AlC (Figure 4a). In principle, this could mean an underetched MAX for all samples. However, this is not the case for several samples including sample A-2-45C-HF. For this sample, a low etching reaction yield (25 wt %) was obtained and morphology and analytical studies indicated a top layer composed of mostly carbon. Another sample, A-6-40C-HF, processed using the same precursor and similar etching conditions (Table 1), led to the same yield (25 wt %) and no delamination. Thus, sample A-2-45C-HF would not lead to delamination either. The XRD patterns of both samples were practically the same (Figure 5). Altogether, this indicated that the A-2-45C-HF sample consisted of unetched material constituted by the cores of large particles overetched only at the surface (shells) (Figure 2d–f, Figure 4b). Since the remaining shells were amorphous, as suggested by SEM, they could not be detected by XRD (Figure 4b). The overetched material (75 wt %) was then constituted by material coming off from overetched shells and a high fraction of micrometer and submicrometer sized particles, both dissolved away during the etching reaction. All the gathered evidence, morphology, crystal structure, elemental composition, and quantified yield, indicated a core–shell etching pattern (Figure 4b).

**Figure 6:**
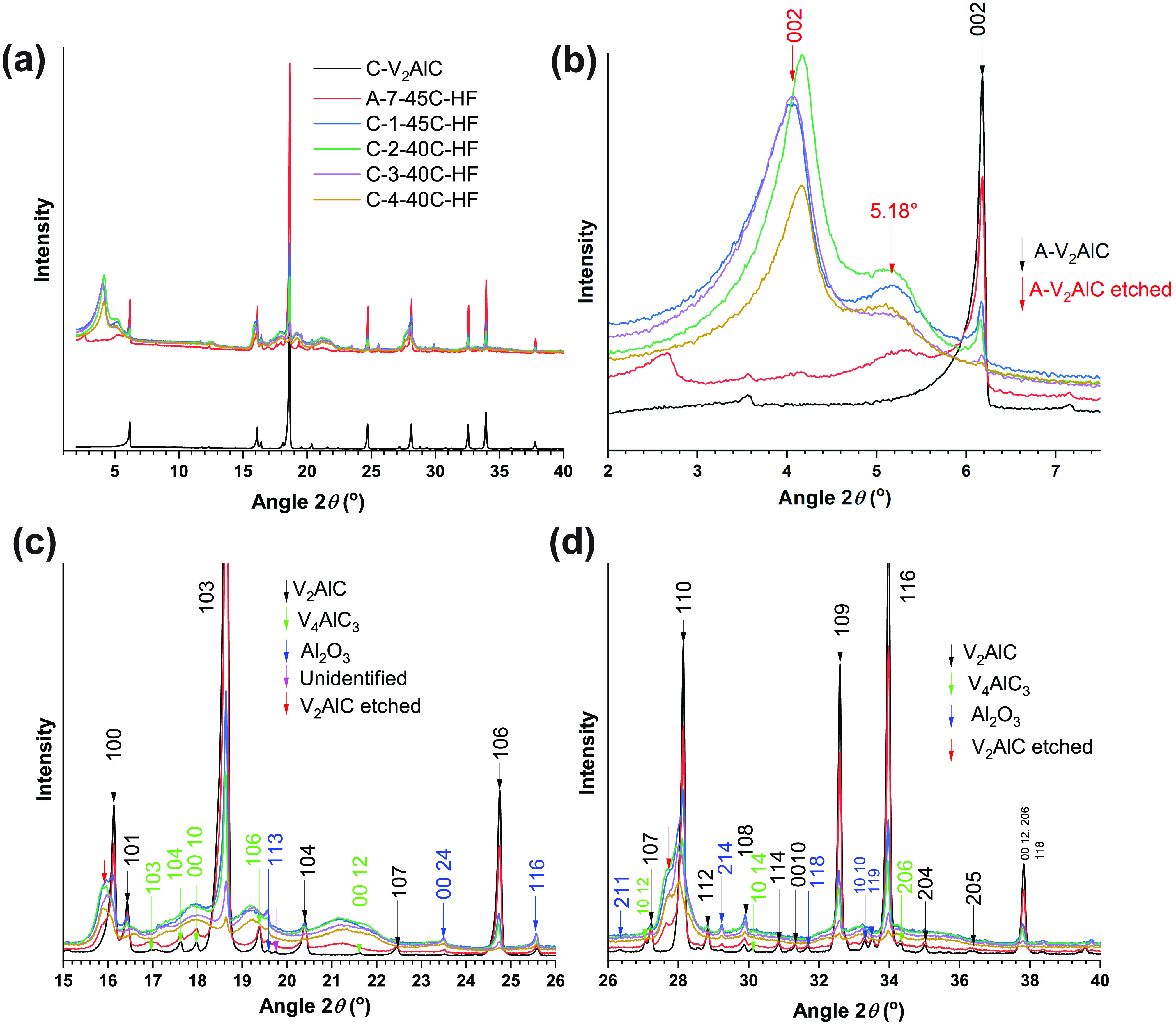
(a) XRD patterns of samples of sample of A-7-45C-HF (early etching) and samples of set C that lead to delamination (Table 1). (b–d) High magnification at specific 2θ ranges.

Another sample, A-7-45C-HF, again processed using the same precursor and etching conditions except for the use of a smaller reaction vessel (60 mL(small) instead of 250 mL(large)) led to a higher yield (84%) and to some degree of delamination (Table 1). However, the XRD pattern of sample A-7-45C-HF had subtle differences as compared to those of samples A-2-45C-HF and A-6-40C-HF. This included broad features at slightly lower angles than the 002 reflection of the A-V_2_AlC precursor, 2θ = 5.3°, and around the corresponding 100 and 110 reflections, and new emerging signals at 2θ = 17° and 2θ = 21.2° (Figure 5). Comparison against well etched samples (Figure 6) confirmed that these are features describing etching but at an early stage.

A third set C of samples was synthesized (Table 1) in a very similar manner than samples A-2-45C-HF and A-(6-8)-TC-Acid (Table 1). As for synthesis of set A, only the mass fraction of the precursor C-V_2_AlC with particles size <36 μm was considered for etching procedures. Notice processing at a temperature of 40–45 °C and the use of the large reaction vessel. The XRD patterns of these samples revealed features at 2θ = 5.2°–5.3° and 2θ = 4°–4.16° (Figure 6b). The latter was assigned to the 002 reflection of the emerging etched phase.^24^ The former will be discussed in section 3.9. The broadening of the reflections 100 and 110 was enhanced for all these samples (Figure 6c,d). A medium yield (53.80 wt %, 60.96 wt %) indicated early overetching of samples C1-45C-HF and C3-40C-HF. A high yield (96 wt %, 97.48 wt %) indicated good etching of samples C2-40C-HF and C4-40C-HF. XRD patterns of all samples of set C are similar, indicating that the slightly overetched material is amorphous. Since for all these samples delamination was obtained, it follows that well etched and slightly overetched samples can lead to delamination. On the other hand, the similarity of the features of the XRD pattern of sample A-7-45C-HF with those of the XRD patterns of samples of set C indicated an early etching of sample A-7-45C-HF (Figure 6a).

Lastly, the sample A-8-45C-HF, etched in similar conditions as A-2-45C-HF and A-6-40C-HF (both with very low yield), had a medium yield (64.3 wt %) and led to no delamination. The XRD patterns of all such samples were very similar (Figure 5). Given the yield and the no delamination result, it is concluded that this is the case of an underetched sample. The XRD pattern then described the poorly etched MAX phase.

The following conclusions can be drawn. XRD patterns of underetched and overetched samples are similar (samples of set A, Figure 4a). The same applies for well etched and early overetched samples (samples of set C, Figure 6). This is explained by the core–shell etching pattern where only cores of the etched material are detected by XRD (Figure 4b). Therefore, results of an etching reaction cannot be judged by only looking at XRD studies. The yield of the reaction and elemental analysis are critical for interpretation of results of etching processes. Yields below < 50 wt % are indicative of overetching, and severe overetching if < 30 wt %, leading to no delamination. High yields, > 80%, are generally correlated with a good etching leading to delamination. A yield of 50–80% is indicative of early overetching when it is followed by effective delamination. Yields of 50–60 wt % are correlated to underetching, when no delamination is observed. The similar yield ranges of the latter two categories is explained by variations in MAX properties from batch to batch, including PSD. Based on these criteria, samples of set A and C are determined as underetched, early etched, well etched, early overetched, and overetched (Table 1).

**Figure 7:**
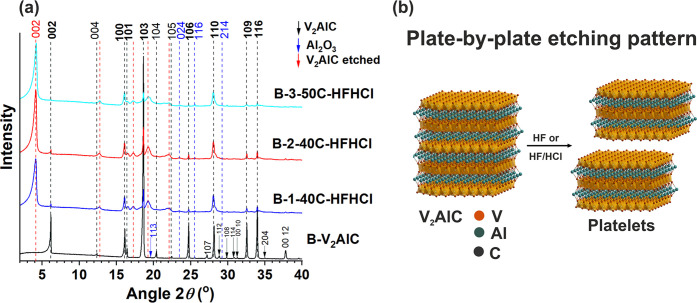
(a) XRD patterns of samples B-1-40C-HFHCl, B-2-40C-HFHCl, and B-3-50C-HFHCl. The main reflections of the V_2_AlC phase are indicated by labels in bold font. (b) Schematic of the plate-by-plate etching pattern followed by samples of set B.

On the other hand, the XRD patterns of samples of set B were similar (Figure 7a). Given the yield, the elemental analysis describing a suitable etching (Figure 1b), and the successful delamination of these samples (Figure S9, Figure S14), these XRD patterns were considered to describe well etched samples (Table 1). Suitable etching was achieved following a plate-by-plate etching pattern (Figure 7b). Here, XRD detected the well etched platelets resulting of a more uniform etching across each MAX precursor particle.

### 3.7 Discussion of Etching Conditions

#### 3.7.1 Reaction Vessel Volume

Since samples A-7-45C-HF (early etched) and A-8-45C-HF (underetched) were processed in the same conditions (Table 1) except for a smaller reaction vessel for the former, this confirmed that smaller reaction vessels favored etching processes. A large volume/diameter ratio of small reaction vessels favored an improved heat transfer across the reactor and also, indirectly, favored a more homogeneous mixing. Both conditions improved the reaction kinetics. However, samples of set C (Table 1) were efficiently etched in large reactions vessels. This indicated that C-V_2_AlC had properties, not further studied, that facilitated etching processes as compared to A-V_2_AlC. MAX properties outweighed reaction conditions as determinant variables leading to a successful etching.

#### 3.7.2 Mixing Efficiency and Reaction Kinetics

Various experimental results pointed out that mixing efficiency implied considerations other than the nominal mixing rate.

The sample A-2-45C-HF, synthesized under the optimized conditions (45 °C, 4 days, mixing rate of 400 rpm), underwent severe overetching (low yield and large V and Al dissolution), which was enhanced at the shell of the particles. Lowering the mixing rate to 100 rpm, for the A-5-45C-HF-100rpm sample, led to a case of an average underetching (Table 1), with etching limited to the shell. This indicated that lowering the mixing rate slowed down the reaction kinetics. However, the higher mixing rate of 400 rpm for the sample A-2-45C-HF did not explain the overetching results. Other experiments using exactly the same etching conditions including the same mixing rate (45 °C, 4 days, mixing rate of 400 rpm) led to a variety of etching results: A-2-45C-HF (overetched), A-7-45C-HF (early etched), and A-8-45C-HF (underetched). This ruled out the nominal mixing rate as a determinant variable of the average degree of etching of samples.

Such discrepancies can only be explained as rooted in a scenario involving multiple and highly correlated variables. First, considering the physics and engineering of mixing processes, beyond a nominal mixing rate is critical. The efficiency of mixing processes is determined by dimensions of the effective reaction volume relative to the geometry and dimensions of the mixer. This will, in turn, determine mass transport variables affecting the reaction materials and ultimately the reaction kinetics (a function of effective reaction temperature, acid concentration, and particle surface area to volume ratio^17^). In addition, the wall thickness and material of the reaction vessel will largely determine the heat retained and thus the effective temperature inside the reaction vessel. The very different etching results obtained while using the very same mixing rate could be explained based on these considerations. In practice, we have observed that mixing using a magnetic bar is not the best method to ensure a homogeneous mixing across the reaction vessel. The bar can go off center, and such random variations most likely contributed to a poor mixing efficiency and ultimately to poor reproducibility of etching results. Future work must involve an in-depth investigation of mixing processes, mass transport phenomena, and their correlation with reaction kinetics.

#### 3.7.3 An Early Overetching at MAX Particle’s Surface

The overetching of sample A-2-45C-HF can be explained as the result of a combination of various conditions. First, a deficient mixing described above. Second an overetching of V at MAX particles surfaces while adding the MAX to the acid. Since the etching reaction is exothermic (eq 2), it is recommended to add the MAX phase to the acid slowly and in very small quantities to avoid overheating. Overheating may have well accelerated etching of V at the surface of MAX particles triggering etching toward the core.

#### 3.7.4 The MAX Properties

Morphology and XRD studies confirmed that samples of set A followed a core–shell etching pattern while samples of set B followed a plate-by-plate etching pattern. This is rooted in highly correlated MAX properties: stoichiometry, phase composition, crystal structure, and morphology. It is known that by varying the element proportions in MAX synthesis, the morphology of resulting crystals and properties of derived MXenes can be largely modified. Particularly, synthesizing a MAX phase with excess Al has produced Al-Ti_3_AlC_2_ with an enhanced stability to oxidation.^20^ On the other hand, recently, it has been reported that MAX, and derived MXenes, synthesized using stoichiometric element proportions led to incorporation of O atoms in the C layers of MAX and MXenes.^19^ Oxycarbides were not formed when utilizing an Al excess in the MAX synthesis.^19^ The presence of compositional oxygen on MAX and MXenes has major implications on properties, including reactivity and structural stability.^19^ Here, A-V_2_AlC and B-V_2_AlC were synthesized using 2:1.3:1 and 2:1.1:0.9 V:Al:C mol ratios, respectively. The former considered a slight excess of Al. Whether oxycarbides are present on the latter but not on the former was not studied. Surely, these different stoichiometries led to MAX phases with different properties that were reflected in the different etching patterns and yields of etching reactions. Further specialized studies will be necessary to optimize MAX synthesis procedures in view of optimizing yield and quality of etched materials and stability and properties of derived MXenes.

#### 3.7.5 Other Variables

Further XRD (section 3.8) and XPS (section 3.10) studies revealed that Al_2_O_3_ was formed on overetched samples, which indicated a larger and preferential loss of V over Al. XPS showed that Al_2_O_3_ was also formed on well etched (high etching yield) samples synthesized at optimized temperatures (35–40 °C) (Figure S19). This indicated that Al_2_O_3_ readily forms at the surface of etched particles as soon as Al is exposed to an O_2_ containing environment, and it is not unique to high reaction temperatures.

### 3.8 XRD Analysis of Overetched Samples and Reaction Mechanisms

**Figure 8:**
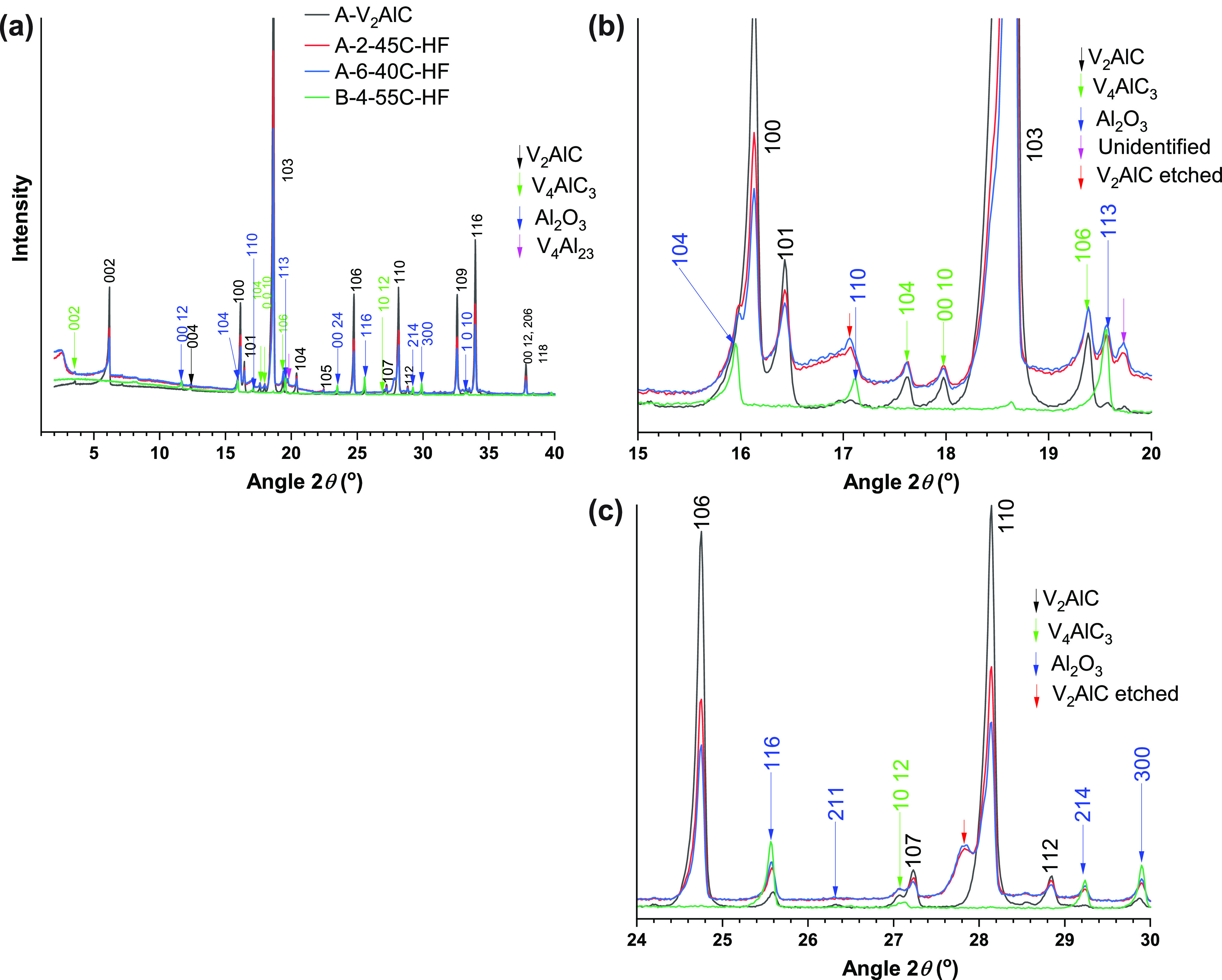
(a) XRD patterns of samples A-2-45C-HF and A-6-40C-HF (set A) and B-4-55C-HF (set B), all representative cases of overetched samples with a low etching yield and that led to no delamination. (b, c) High magnification at specific 2θ ranges.

XRD analysis of overetched samples, as described by a low yield, revealed the presence of an emerging Al_2_O_3_ phase (Figure 8). This applied to samples A-2-45C-HF and A-6-40C-HF (Table 1) and another sample that was intentionally overetched using a high temperature of 55 °C, sample B-4-55C-HF (Table 1). The emergence of the 104 reflection of Al_2_O_3_ “under” the 100 reflection of the V_2_AlC phase was observed typically as a reflection “splitting” (Figure 8b). The 116, 214, and 300 reflections of the Al_2_O_3_ phase became stronger for overetched samples (Figure 8c). The presence of Al_2_O_3_ was confirmed by XPS studies (section 3.10).

In view of these results it is worth discussing the chemistry of etching reactions in regard to the formation of Al-containing species. From the elemental composition analysis, it is clear that as etching advances, V etches away together with Al, e.g. sample A-2-45C-HF (Figure 1a), but clearly in general Al never gets completely etched away in a sample. Previous reports establish that metallic Al exposed to HF would react according to eq 2 to form AlF_3_.^1^
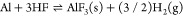
2
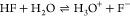
3
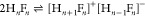
4
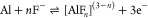
5
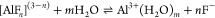
6

However, this chemistry assumes a complete dissociation of HF in H_3_O^+^ ions (eq 3), which is not the case. In highly concentrated HF, the acidity of HF is no longer based on H_3_O^+^ (eq 3) but rather on other more acidic species (H_*n*+1_F_*n*_)^+^ (eq 4).^25,26^ The acidity of HF at high concentrations is rather measured by the Hammett function *H*_0_, which increases (more negative) as the concentration of the acid increases. For HF 48 wt % (27.5 M), *H*_0_ = −3.8, and it is around this concentration where the *H*_0_ function increases sharply. Since H_2_ evolution has been observed in etching procedures, it is likely that the equilibrium of the acid is still partially based on eq 3 but also on eq 4. The latter will bring as a consequence a partial conversion to AlF_3_ (eq 2). Accordingly, it is likely that species of the type [AlF_*n*_]^(3–*n*)^ are formed (eq 5).^27^

On the other hand, AlF_3_ cannot exist in etching reactions in a solid state. Precipitation of AlF_3_ is favored in millimolar concentrations of HF,^27^ so it is unlikely that AlF_3_ will exist as a solid phase in highly concentrated HF in the MAX etching reactions. Rather, species of the type [AlF_*n*_]^(3–*n*)^, including AlF_3_(aq) in the dissociated state, will be present (eq 6).^21^ These species would then be washed away during washing steps of etching products and are not expected to be present in powder reaction products. This was confirmed by XPS studies (section 3.10).

On the other hand, according to the Pourbaix diagram of Al, Al_2_O_3_, in the highly acidic conditions of the etching procedures, dissociates into Al^+^/Al^3+^.^28^ However, in neutral conditions, Al_2_O_3_ is stable.^28^ Therefore, Al_2_O_3_ exposed to HF cannot exist during etching procedures, as it would dissociate into ions and react according to eqs 5 and 6.^29^ However, if this phase is in the core of large MAX particles or particle aggregates, it may survive the etching. Therefore, the Al_2_O_3_ detected by XRD on etched materials may well be part of the minor secondary fraction originally present on the precursor MAX but mostly a product of oxidation in air of unetched Al at particles surfaces during washing steps, when the pH is raised to neutral. Since the amount and conversion to Al_2_O_3_ were clearly enhanced for overetched samples, this indicated that more V than Al is lost during such etching procedures, resulting in Al exposed to imminent oxidation in the standard atmosphere of postetching steps.

### 3.9 Study of the Crystal Structure Properties of Well Etched Samples

Analysis of the XRD patterns of the etched sample B-3-50C-HFHCl revealed several events with crystal structure (Figure S12). First, it is clear that upon etching the *h*0*l* reflections decreased intensity, which indicated the transition of the 3D structure to the 2D structure upon etching away of the Al. Second, new intensities emerged. Elucidation of the crystal structure of etched samples was attempted using the Le Bail method. Information about the fitting and a full discussion are provided in the Supporting Information (sections S10 and S11). The fitting revealed that it is a mix of V_2_AlC (precursor), etched V_2_AlC and Al_2_O_3_ (Figure S11). However, the etched product, as revealed here by morphology studies, is a mix of fractions of material with different etching degrees. This and the presence of a fraction of fully delaminated V_2_C, especially at the surface of particles, was not taken into account in the refinement. Therefore, such refinement should be taken as an approximation to the solution of the problem. A full study of crystal structure of etched MAX phases will be addressed in a separate work.

**Figure 9:**
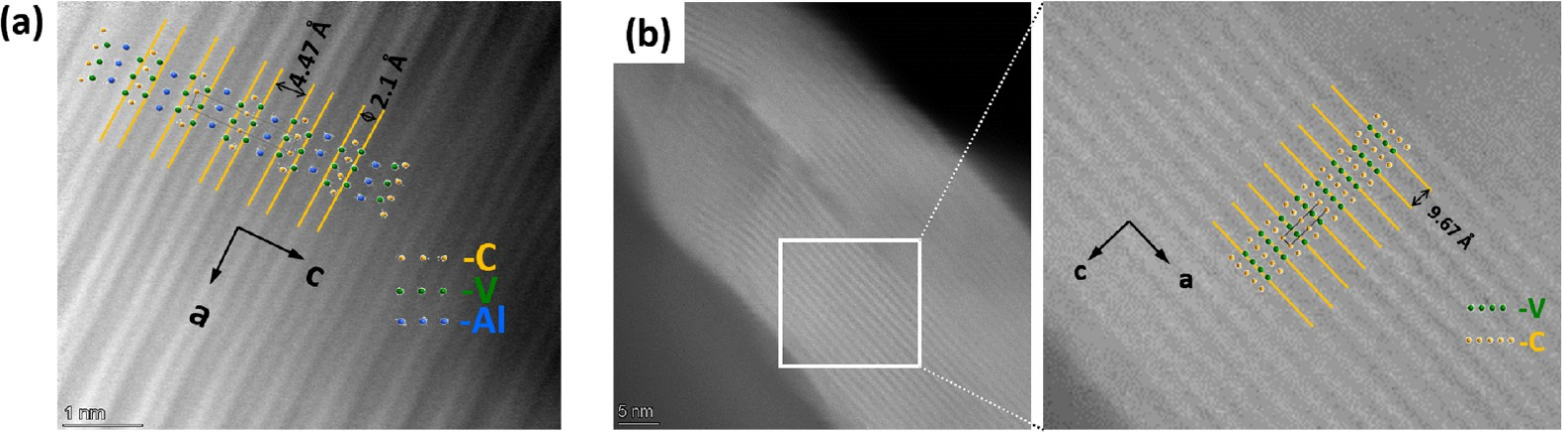
HRTEM images of (a) B-V_2_AlC and (b) etched sample B-3-50C-HFHCl. Measured lattice spaces are indicated. For the sample B-V_2_AlC, space *d* = 2.1 Å + 4.47 Å = 6.57 Å, which led to a lattice parameter *c* = 2*d* = 13.14 Å. From XRD data, the lattice parameter *c* = 2*d* = 13.14814(6) Å (Table S4), which agrees well with the TEM measurements. For the sample B-3-50C-HFHCl, space *d* = 9.67 Å, *c* = 2*d* = 19.34 Å. From XRD data, the lattice parameter *c* = 19.3377(4) Å (Table S6), which agrees well with the TEM measurements. The atom models were built using the lattice parameters of the corresponding refined XRD data (Tables S4 and S6).

The rise of a new broad reflection at 2θ = 4.18° (lattice parameter *c* = 19.4 Å) was assigned to the 002 reflection of the new etched phase or mix of etched phases (Figure S12b). In parallel, the intensity of the 002 reflection of the precursor B-V_2_AlC at 2θ = 6.18° (lattice parameter *c* = 13.14 Å) decreased. The increase of the *c* lattice parameter of the etched product, with respect to the precursor, described the removal of Al in between *ab* planes. This was confirmed by high resolution transmission electron microscopy (HRTEM) studies (Figure 9).

There were drastic differences in the XRD patterns at low angles (2θ = 2–7°) for samples etched in HF/HCl, e.g., samples of set B (Figure 7a) and samples etched in only HF, e.g., samples of set C (Figure 6b). Further insights about it were revealed by thermal treatment under Ar atmosphere of B-3-50C-HFHCl and C1-45C-HF samples. In experiment 1, samples were processed for 5 h at 200 °C. In experiment 2, thermal treatment was done in two stages, the first one at 120 °C for 3 h and the second one at 450 °C for 5 h.

**Figure 10:**
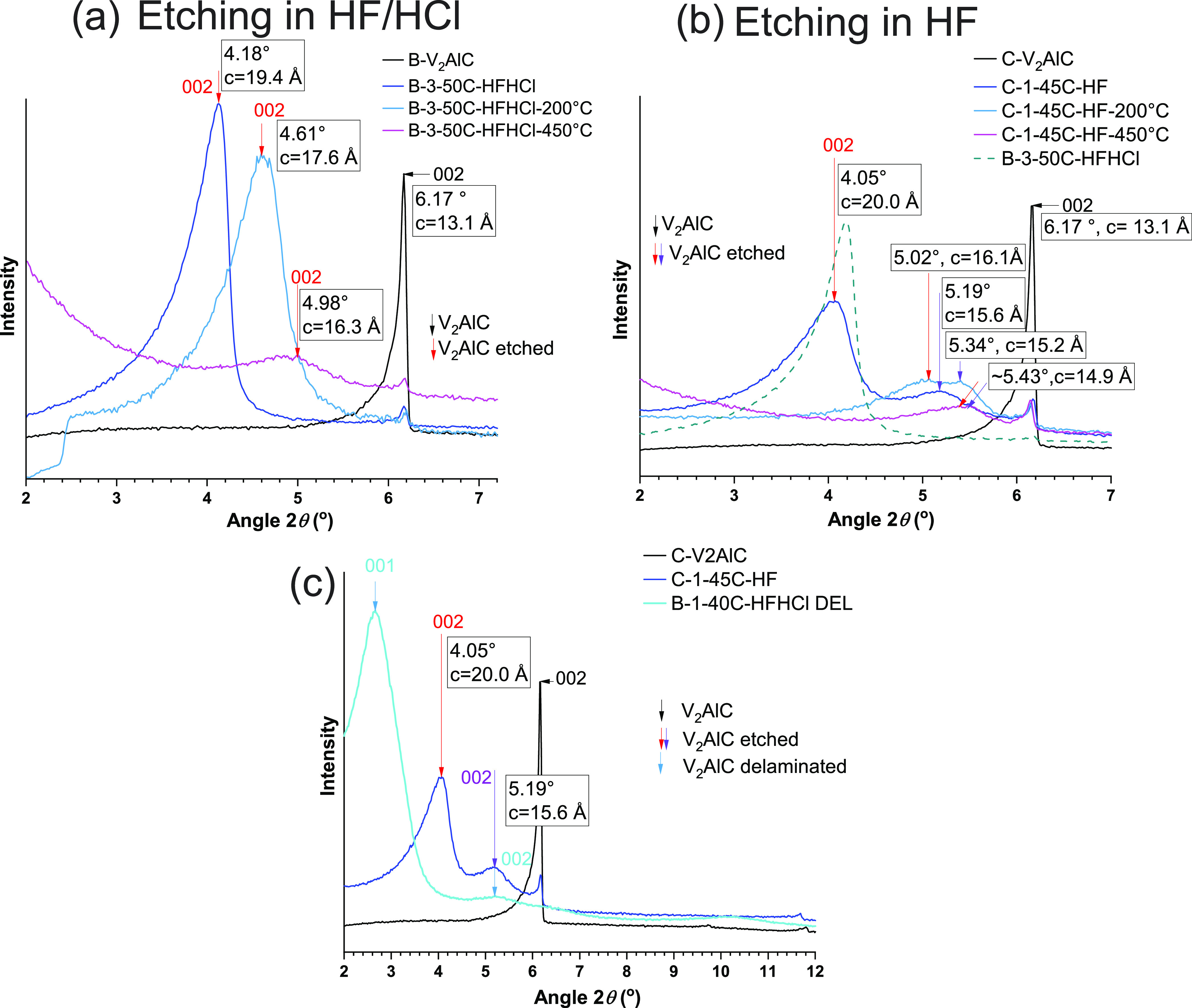
XRD patterns of (a) sample B-3-50C-HFHCl, etched in a mix of HFHCl, as prepared and upon thermal treatment, (b) sample C-1-45C-HF, etched in only HF, as prepared and upon thermal treatment, (c) sample C-1-45C-HF and sample B-1-40C-HFHCl-DEL (B-1-40C-HFHCl after delamination). Corresponding V_2_AlC phases are added for reference. In (b) the XRD pattern of sample B-3-50C-HFHCl is added as reference.

For the sample B-3-50C-HFHCl, etched in HF/HCl, the etched phase was described by a 002 reflection at 2θ = 4.18° (*c* = 19.44 Å, space *d* = 9.72 Å) (Figure 10a). Upon thermal treatment at 200 °C, this reflection shifted to 2θ = 4.61° (*c* = 17.64 Å, space *d* = 8.82 Å). Subtracting the *d* space magnitudes of “wet” (prior to thermal treatment) and thermally treated samples gives the interlayer spacing change of 0.90 Å.^24^ Upon further thermal treatment at 450 °C, the reflection shifted to 2θ = 4.98° (*c* = 16.32 Å, space *d* = 8.16 Å), which corresponded to an interlayer space change of 1.56 Å. Considering a diameter of water molecules of 2.8 Å, such interlayer spaces cannot be attributed to a full layer of water molecules. However, the “wet” sample had undergone a drying procedure prior to XRD testing, including a step of 40 °C for 5 h (see Experimental Methods). Therefore, changes in interlayer spacings upon thermal treatment most likely involved further partial dehydration at 200 °C. At 450 °C, the broadening of reflections, especially the 002, indicated structural changes (Figure S13).

In contrast, the sample C-1-45C-HF, etched in HF, showed two reflections at low angles (Figure 10b): one at 2θ = 4.05° (*c* = 20.07 Å, space *d* = 10.03 Å). Upon thermal treatment at 200 °C, it shifted to 2θ = 5.02° (*c* = 16.19 Å, space *d* = 8.09 Å). This corresponded to a change in interlayer spacing of 1.93 Å. As in the case of the sample B-3-50C-HFHCl, this indicated a continued partial dehydration. The second reflection was at 2θ = 5.19° (*c* = 15.66 Å, space *d* = 7.83 Å). Upon thermal treatment at 200 °C, it shifted to 2θ = 5.34° (*c* = 15.22 Å, space *d* = 7.61 Å), corresponding to a interlayer space change of 0.21 Å. Upon further thermal treatment at 450 °C, this reflection did not shift significantly. This reflection was particularly broad before and after thermal treatment. Altogether this indicated its structural nature, and it might well correspond to a phase or mix of phases with a different degree of etching. Furthermore, comparison to the XRD pattern of a delaminated film, B-1-40C-HFHCl-DEL, confirmed that this reflection may have a significant contribution of a fully delaminated phase, which has a 002 reflection at the same 2θ = 5.19° (Figure 10c).

Since the occurrence of two such reflections is reproducible across samples etched in HF (Figure 6b), this supports the view that etching in HF favors heterogeneous processes that give place to a fraction of fully delaminated V_2_C at the surface of V_2_AlC particles. This supports the core–shell etching pattern proposed for the etching processes undergone for samples of set A. In contrast, etching in HF/HCl seems to favor a more homogeneous etching process, described by a “monomodal” *c* lattice parameter. In practice, we have realized that the mix of HF/HCl favors the swelling/wetting of the etched material, which is observed as large volume to mass ratio (and higher total weight due to the larger quantity of embedded water in the powder), as compared to materials etched in HF. This swelling effect describes a better access of acids into the MAX phase, favoring a more homogeneous etching within and across particles. XPS findings revealed an enhanced surface chemical functionalization when etching in HF/HCl as compared to etching in HF only (section 3.10).

Other structural aspects revealed by thermal treatment experiments are discussed in the Supporting Information (section S12).

### 3.10 X-ray Photoelectron Spectroscopy Studies

The precursors A-V_2_AlC and B-V_2_AlC were analyzed by XPS. The full details of data modeling are given in the Supporting Information (section S14). Analysis of the V 2p spectral signal revealed the presence of the V–C bonds corresponding to the carbide but also the chemical environment of vanadium oxides (Figure S15, Tables S7 and S8). Thus, in agreement with the findings by analytical methods, it is confirmed that V_2_AlC precursors are indeed surface covered by vanadium oxides.

The etched samples of set A and set B were analyzed by XPS. The construction of models for the V 2p and O 1s transitions was guided by previously reported models for Ti_3_C_2_T_*x*_.^30^ The models are shown in Figures S16 and S17. Details of the model construction are given in the Supporting Information (section S15). The proposed spectral components are reported for representative samples A-1-35C-HF and B-1-40C-HFHCl in Tables S9 and S10, respectively.

The analysis of a fourth set of samples, set D, was necessary to provide suitable chemical contrast to aid in the identification of the proposed spectral components for the O 1s and other spectral regions. The synthesis conditions are as described in Table 1. Samples were prepared in HF at different temperatures of 35 °C, 40 °C, and 50 °C. Then, delamination of sample D-1-35C-HF rendered sample D-1-35C-HF-DEL.

**Figure 11:**
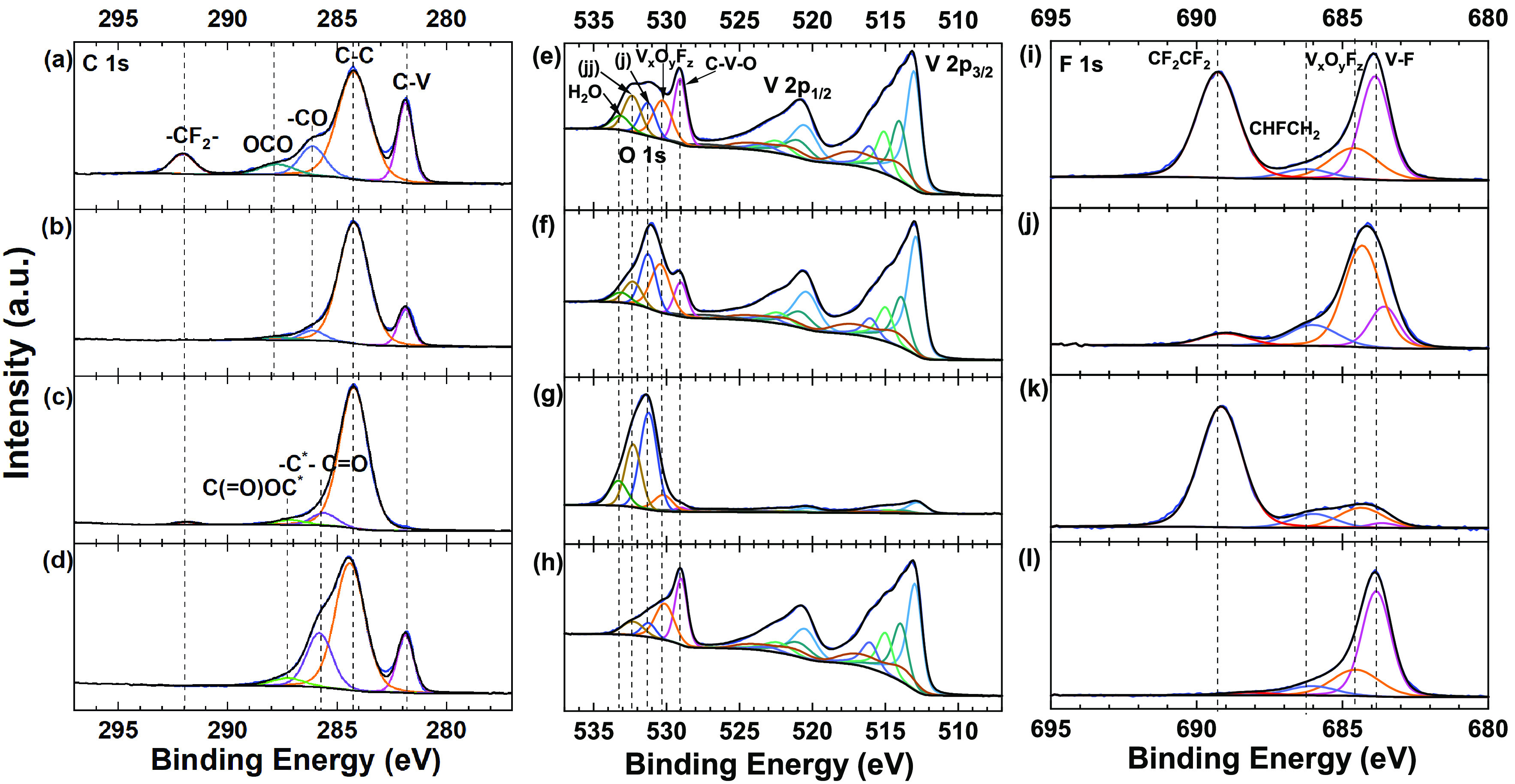
XPS spectra of samples of set D: (a, e, i) D-1-35C-HF, (b, f, j) D-2-40C-HFHCl, (c, g, k) D-3-50C-HFHCl, and (d, h, l) D-1-35C-HF-DEL (Table 1) showing (a–d) C 1s, (e–h) V 2p and O 1s, and (i–l) F 1s spectral regions.

A full description of the models for XPS data of set D for all spectral regions, C 1s, V 2p, O 1s, F 1s, Al 2s, and Al 2p, is given in the Supporting Information (section S16). The models for the V 2p and the O 1s spectral regions were built using the same considerations as for sets A and B and using the very same components. Namely, spectral components of the V 2p region included, from low to high binding energy (BE), C-V-O/O/O, C-V-F/O/O, C-V-F/F/O, and C-V-F/F/F chemical functionalities and a component for vanadium oxides (Table S12, Figure 11e–h). Spectral components of the O 1s region included a component for C–V–O bonds and a component V_*x*_O_*y*_F_*z*_ species, derived from reaction of V with O (oxidation) and F atoms^30^ (Figure 11e–h). The origin of other identified spectral components (j) and (jj) was investigated as described next.

A remarkable contrast of the sample D-3-50C-HF, synthesized at 50 °C, versus samples of sets A and B was a clear degradation of V. This was indicated by the poor V 2p signal (Figure 11g). In parallel, the C-V component of the C 1s spectrum vanished (Figure 11c), which indicated the dissolution of the vanadium carbide. In parallel, the (j) and (jj) components of the O 1s spectra were enhanced (Figures 11g and S20b), which confirmed that these components have a substantial contribution of O species bonded to an element other than V. The corresponding Al 2s spectrum showed components at 119.15 and 120.92 eV that were assigned to Al(OH)_3_ and Al_2_O_3_, respectively^31^ (Figure S19c). The corresponding Al 2p spectrum showed a component at 74.37 eV (Figure S19f), which was clearly enhanced for this sample (Figure S20e) and which most probably had contributions of both species. Based on all these pieces of evidence, it was concluded that the (j) and (jj) spectral components correspond to (j) Al(OH)_3_, and (jj)Al_2_O_3_ species. In addition, note that these components become insignificant in the delaminated sample D-1-35C-HF-DEL (Figures S20b), as expected upon removal of Al. A component at 533.09 eV was assigned to adsorbed water.

An Al 2s component at 117.05 eV was assigned to the V–Al bond (Figure S19b,c, Table S15). In the case of Al 2p, such spectrum is complex due to the overlapping of V 3s and Al 2p signals (Figure S19d–g, Table S16). However, the presence of a component at 71.4–71.61 eV is clear and was assigned to the V–Al bond (Figure S19d–f), clearly vanished for the overetched D-3-50C-HF sample (Figure S19f).

The F 1s spectrum had four components at 683.89 eV, 684.61 eV, 686.23 eV, 689.28 eV, which were assigned to V-F, V_*x*_O_*y*_F_*z*_, (CHFCH_2_)_*n*_, and (CF_2_-CF_2_) groups (Figure 11i–l, Table S14). The assignment of the first component is supported by the data of the sample D-1-35C-HF-DEL (Figure 11l), for which such component was clearly enhanced and proved effective chemical -F functionalization (Figure S20c). The assignment of the fourth component to (CF_2_-CF_2_) groups was supported by the parallel presence of a component at 292.0 eV of the C 1s spectrum, e.g., for sample D-1-40C-HF (Figure 11a). The origin of this species is PTFE contamination from the reaction vessel and stir bar (debris coming from erosion and chemical degradation over time). The third component at 686.23 eV is too low in BE to describe AlF_3_, previously reported at 687.5 eV, for C 1s at 284.8 eV ^32^ (687 eV for V–C at 281.88 eV used here for calibration). According to the same reference, Al 2p for AlF_3_ would be at 77.1 eV (76.53 eV for V–C at 281.88 eV used here for calibration), thus, too high in BE, as compared to the position of the Al 2p component here found at 74.3 eV for the D-3-50C-HF sample. Therefore, the presence of AlF_3_ was discarded.

Now, we discuss relevant chemistry facts of sets A and B, as informed by XPS data. The V 2p spectra of the samples of set A were quite similar (Figure S16) except for the sample A-5-45C-HF-100rpm where the 2p spin–orbit splitting was larger (7.4 eV for all the components and not only the first one). This could describe a different V bonding environment, which might be correlated to the enhanced presence of V_*x*_O_*y*_F_*z*_ species, as revealed by quantification (Figure S18a). This described an enhanced oxidation at a slow (100 rpm) mixing rate. The next significant spectral chemical species in this sample were those described by (j) Al(OH)_3_ and C-V-O spectral components (Figure S18a). This indicated an enhanced reactivity with O of Al and V elements, respectively, at a slow mixing rate.

For the sample A-4-45C-HFHCl, synthesized using HF/HCl, the atomic percentage of the C-V-O and V_*x*_O_*y*_F_*z*_ species was higher as compared to the other species (j) and (jj) (Figure S18a). The same trend was followed for all the samples of set B, synthesized using HF/HCl (Figures S17 and S18b). In addition, analytical methods revealed an enhanced -F functionalization when synthesizing in HF/HCl (Figure S6). All of this indicated that synthesis in HF/HCl favored C-V-O and C-V-F functionalization over secondary reactions producing (j) Al(OH)_3_, and (jj) Al_2_O_3_ components. On the other hand, oxidation to form V_*x*_O_*y*_F_*z*_ species was imminent synthesizing in either HF or HF/HCl. For samples of set A, it is clear that the atomic percentage of (j) and (jj) species follows the same trend across samples, indicating a correlation between the chemistry of these components. The same was observed for samples of set D (Figure S20b), also synthesized in HF.

## 4 Conclusions

Etching processes, using acid-based methods, undergone by two MAX precursors A-V_2_AlC and B-V_2_AlC were studied. The role of a range of reaction variables and precursor material properties were investigated using a broad range of chemical and materials characterization techniques.

Analytical methods confirmed and quantified the nonselectivity of etching processes where not only Al but also V got etched away. Elemental analysis, XRD studies, and quantification of yield of etching reactions were identified as a key set of studies, and not only XRD, necessary to adequately evaluate the quality of etched products. Using this approach, etched products were identified as underetched, early etched, well etched, early overetched, and overetched. Only early overetched and well etched samples led to delamination. Overetching was described by a large mass loss of V and Al, which was correlated with a low yield of reactions. Several reaction variables contributed to overetching including high temperature, mixing inefficiency, and early accelerated etching induced by overheating at early stages of the etching reactions.

Importantly, MAX properties were found to be determinant in etching processes. This was evidenced by the very different etching products obtained from the A-V_2_AlC and B-V_2_AlC starting materials. Analytical methods, morphology, and XRD studies identified that the former followed a core–shell etching pattern whereas the latter followed a plate-by-plate etching pattern. Core–shell etched products derived from A-V_2_AlC were highly heterogeneous and included under- and overetched materials. Plate-by-plate etched products derived from B-V_2_AlC were well etched products with high reaction yields. Overall studies indicated that the crucial MAX properties determining etching results were particle size and PSD, crystal structure, and elemental composition. In general, a broad PSD was found to be unfavorable to optimize reaction conditions and to lead to a high heterogeneity of morphology and degree of etching of products. XRD studies revealed the presence of secondary phases, which may have contributed to the heterogeneous etching processes in B-V_2_AlC. Previous studies suggest that differences in elemental composition of MAX phases can directly influence the behavior of MAX phases during etching processes such as stability due to crystal defects and/or the presence of atoms other than M, A, and X.

The need for optimization of reactors was recognized, particularly regarding dimensions, geometry, and mixing mechanisms, which have a direct impact on mixing efficiency, heat transfer, and reaction kinetics.

Further studies revealed details of reactions mechanisms and chemistry of etched products. XPS studies revealed the presence of vanadium oxides at the MAX surface, which did not have an impact on etching processes. XRD studies of overetched samples revealed the presence of Al_2_O_3_. XPS confirmed that overetching led to dissolution of V atoms and an increase of oxidation involving aluminum atoms resulting in the formation of Al_2_O_3_ and Al(OH)_3_. Revision of reaction mechanisms indicated that Al_2_O_3_ cannot be formed (neither exist as a solid product) during the etching reaction but rather by oxidation of unetched Al during postetching steps. Similarly, the secondary product of etching reactions AlF_3_ cannot precipitate during the etching reaction but it is rather present in the dissociated state in the aqueous reaction mix. Consequently, it is mostly washed away during postetching steps. XPS studies confirmed the absence of AlF_3_ in etched products.

Further studies of well etched materials revealed key differences of etching products according to the type of acid used. XRD analysis of thermally treated well etched materials revealed that etching in HF led to two fractions of etched materials described by two different reflections at low 2θ angles. In contrast etching in HF/HCl typically led to a material with a single *c* lattice parameter indicating a more homogeneous etching process. These findings were complemented with XPS studies, which indicated a more efficient surface chemical functionalization when etching in the HF/HCl mix, as compared to etching in HF only.

Our work revealed various areas of research in MXene synthesis that need attention: MAX synthesis in correlation with the properties of the derived MXene, optimization/engineering of reactors, and in-depth understanding of chemical mechanisms. Further understanding of acid-based etching methods as well as the development of alternative methods is clearly an area of opportunity for further contribution.

## Supporting Information Available

Further description of experimental methods; particle size studies of V_2_AlC; synthesis of etched materials; design of synthesis experiments; Rietveld refinement of XRD patterns of A-V_2_AlC and B-V_2_AlC; V_2_AlC chemical composition; further element analysis of etched samples of sets A and B; optical images of products of etching reactions of samples of set A and set B; optical images of delamination products of samples of set B; additional SEM images of etched samples of sets A and B; Rietveld refinement of the XRD pattern of the etched B-3-50C-HFHCl sample; discussion on the crystal structure of the etched B-3-50C-HFHCl sample; discussion of XRD data of thermally treated B-3-50C-HFHCl sample; XRD data of delaminated material obtained from sample B-3-50C-HFHCl; XPS of A-V_2_AlC and B-V_2_AlC precursors; XPS of etched samples, sets A and B; XPS of samples of set D (PDF)

## References

[ref1] Naguib M.; Mashtalir O.; Carle J.; Presser V.; Lu J.; Hultman L.; Gogotsi Y.; Barsoum M. W. Two-Dimensional Transition Metal Carbides. ACS Nano 2012, 6, 1322–1331. 10.1021/nn204153h. 22279971

[ref2] Wyatt B. C.; Rosenkranz A.; Anasori B. 2D MXenes: Tunable Mechanical and Tribological Properties. Adv. Mater. 2021, 33, 2007973 10.1002/adma.202007973. 33738850

[ref3] Gogotsi Y.; Anasori B. The Rise of MXenes. ACS Nano 2019, 13, 8491–8494. 10.1021/acsnano.9b06394. 31454866

[ref4] Anasori B.; Lukatskaya M. R.; Gogotsi Y. 2D Metal Carbides and Nitrides (MXenes) For Energy Storage. Nat. Rev. Mater. 2017, 2, 16098 10.1038/natrevmats.2016.98.

[ref5] VahidMohammadi A.; Rosen J.; Gogotsi Y. The World of Two-Dimensional Carbides and Nitrides (MXenes). Science 2021, 372, eabf1581 10.1126/science.abf1581. 34112665

[ref6] Mashtalir O.; Naguib M.; Mochalin V. N.; Dall’Agnese Y.; Heon M.; Barsoum M. W.; Gogotsi Y. Intercalation and Delamination of Layered Carbides and Carbonitrides. Nat. Commun. 2013, 4, 1716 10.1038/ncomms2664. 23591883

[ref7] Naguib M.; Unocic R. R.; Armstrong B. L.; Nanda J. Large-Scale Delamination of Multi-Layers Transition Metal Carbides and Carbonitrides “MXenes”. Dalton Trans 2015, 44, 9353–9358. 10.1039/C5DT01247C. 25912071

[ref8] Sang X.; Xie Y.; Lin M.-W.; Alhabeb M.; Van Aken K. L.; Gogotsi Y.; Kent P. R. C.; Xiao K.; Unocic R. R. Atomic Defects in Monolayer Titanium Carbide (Ti_3_C_2_T_*x*_) MXene. ACS Nano 2016, 10, 9193–9200. 10.1021/acsnano.6b05240. 27598326

[ref9] Kim Y.-J.; Kim S. J.; Seo D.; Chae Y.; Anayee M.; Lee Y.; Gogotsi Y.; Ahn C. W.; Jung H.-T. Etching Mechanism of Monoatomic Aluminum Layers during MXene Synthesis. Chem. Mater. 2021, 33, 6346–6355. 10.1021/acs.chemmater.1c01263.

[ref10] Alhabeb M.; Maleski K.; Anasori B.; Lelyukh P.; Clark L.; Sin S.; Gogotsi Y. Guidelines for Synthesis and Processing of Two-Dimensional Titanium Carbide (Ti_3_C_2_T_*x*_ MXene). Chem. Mater. 2017, 29, 7633–7644. 10.1021/acs.chemmater.7b02847.

[ref11] Han M.; Maleski K.; Shuck C. E.; Yang Y.; Glazar J. T.; Foucher A. C.; Hantanasirisakul K.; Sarycheva A.; Frey N. C.; May S. J.; Shenoy V. B.; Stach E. A.; Gogotsi Y. Tailoring Electronic and Optical Properties of MXenes through Forming Solid Solutions. J. Am. Chem. Soc. 2020, 142, 19110–19118. 10.1021/jacs.0c07395. 33108178

[ref12] Halim J.; Kota S.; Lukatskaya M. R.; Naguib M.; Zhao M.-Q.; Moon E. J.; Pitock J.; Nanda J.; May S. J.; Gogotsi Y.; Barsoum M. W. Synthesis and Characterization of 2D Molybdenum Carbide (MXene). Adv. Funct. Mater. 2016, 26, 3118–3127. 10.1002/adfm.201505328.

[ref13] Li Y.; Shao H.; Lin Z.; Lu J.; Liu L.; Duployer B.; Persson P. O. Å.; Eklund P.; Hultman L.; Li M.; Chen K.; Zha X.-H.; Du S.; Rozier P.; Chai Z.; Raymundo-Piñero E.; Taberna P.-L.; Simon P.; Huang Q. A General Lewis Acidic Etching Route for Preparing MXenes With Enhanced Electrochemical Performance in Non-aqueous Electrolyte. Nat. Mater. 2020, 19, 894–899. 10.1038/s41563-020-0657-0. 32284597

[ref14] Urbankowski P.; Anasori B.; Makaryan T.; Er D.; Kota S.; Walsh P. L.; Zhao M.; Shenoy V. B.; Barsoum M. W.; Gogotsi Y. Synthesis of Two-Dimensional Titanium Nitride Ti_4_N_3_ (MXene). Nanoscale 2016, 8, 11385–11391. 10.1039/C6NR02253G. 27211286

[ref15] Arole K.; Blivin J. W.; Saha S.; Holta D. E.; Zhao X.; Sarmah A.; Cao H.; Radovic M.; Lutkenhaus J. L.; Green M. J. Water-dispersible Ti_3_*C*_2_T_*z*_ MXene Nanosheets by Molten Salt Etching. iScience 2021, 24, 103403 10.1016/j.isci.2021.103403. 34849467 PMC8607195

[ref16] Khazaei M.; Ranjbar A.; Esfarjani K.; Bogdanovski D.; Dronskowski R.; Yunoki S. Insights Into Exfoliation Possibility of MAX Phases to MXenes. Phys. Chem. Chem. Phys. 2018, 20, 8579–8592. 10.1039/C7CP08645H. 29557432

[ref17] Anayee M.; Shuck C. E.; Shekhirev M.; Goad A.; Wang R.; Gogotsi Y. Kinetics of Ti_3_AlC_2_ Etching for Ti_3_C_2_T_*x*_ MXene Synthesis. Chem. Mater. 2022, 34, 9589–9600. 10.1021/acs.chemmater.2c02194.

[ref18] Kim Y.; Gkountaras A.; Chaix-Pluchery O.; Gélard I.; Coraux J.; Chapelier C.; Barsoum M. W.; Ouisse T. Elementary Processes Governing V_2_AlC Chemical Etching in HF. RSC Adv. 2020, 10, 25266–25274. 10.1039/D0RA00842G. 35517448 PMC9055248

[ref19] Michałowski P. P.; Anayee M.; Mathis T. S.; Kozdra S.; Wójcik A.; Hantanasirisakul K.; Jóźwik I.; Piatkowska A.; Możdżonek M.; Malinowska A.; Diduszko R.; Wierzbicka E.; Gogotsi Y. Oxycarbide MXenes and MAX Phases Identification Using Monoatomic Layer-By-Layer Analysis With Ultralow-Energy Secondary-Ion Mass Spectrometry. Nat. Nanotechnol. 2022, 17, 1192–1197. 10.1038/s41565-022-01214-0. 36138199

[ref20] Mathis T. S.; Maleski K.; Goad A.; Sarycheva A.; Anayee M.; Foucher A. C.; Hantanasirisakul K.; Shuck C. E.; Stach E. A.; Gogotsi Y. Modified MAX Phase Synthesis for Environmentally Stable and Highly Conductive Ti_3_C_2_ MXene. ACS Nano 2021, 15, 6420–6429. 10.1021/acsnano.0c08357. 33848136

[ref21] Bard A. J.; Parsons R.; Jordan J. Standard Potentials in Aqueous Solutions; Monographs in Electroanalytical Chemistry and Electrochemistry; Marcel Dekker Inc.: New York, USA, 1985.

[ref22] Bak S.-M.; Qiao R.; Yang W.; Lee S.; Yu X.; Anasori B.; Lee H.; Gogotsi Y.; Yang X.-Q. Na-Ion Intercalation and Charge Storage Mechanism in 2D Vanadium Carbide. Adv. Energy Mater. 2017, 7, 1700959 10.1002/aenm.201700959.

[ref23] Thakur R.; VahidMohammadi A.; Moncada J.; Adams W. R.; Chi M.; Tatarchuk B.; Beidaghi M.; Carrero C. A. Insights Into the Thermal and Chemical Stability of Multilayered V_2_CT_*x*_ MXene. Nanoscale 2019, 11, 10716–10726. 10.1039/C9NR03020D. 31120085

[ref24] Shekhirev M.; Shuck C. E.; Sarycheva A.; Gogotsi Y. Characterization of MXenes at Every Step, From Their Precursors to Single Flakes and Assembled Films. Prog. Mater. Sci. 2021, 120, 100757 10.1016/j.pmatsci.2020.100757.

[ref25] Liang J.-N. J. The Hammett Acidity Function for Hydrofluoric Acid and Some Related Superacid Systems. Ph.D. Thesis, McMaster University, Hamilton, Canada, 1976.

[ref26] Hyman H. H.; Kilpatrick M.; Katz J. J. The Hammett Acidity Function H_0_ for Hydrofluoric Acid Solutions. J. Am. Chem. Soc. 1957, 79, 3668–3671. 10.1021/ja01571a016.

[ref27] Estruga M.; Meng F.; Li L.; Chen L.; Li X.; Jin S. Large-scale Solution Synthesis of α-AlF_3_ ·3H_2_O Nanorods under Low Supersaturation Conditions and their Conversion to Porous β-AlF_3_ Nanorods. J. Mater. Chem. 2012, 22, 20991–20997. 10.1039/c2jm33782g.

[ref28] Pourbaix M. Atlas of Electrochemical Equilibria in Aqueous Solutions; National Association of Corrosion Engineers, 1974.

[ref29] Chupas P. J.; Corbin D. R.; Rao V. N. M.; Hanson J. C.; Grey C. P. A Combined Solid-State NMR and Diffraction Study of the Structures and Acidity of Fluorinated Aluminas: Implications for Catalysis. J. Phys. Chem. B 2003, 107, 8327–8336. 10.1021/jp0300905.

[ref30] Natu V.; Benchakar M.; Canaff C.; Habrioux A.; Célérier S.; Barsoum M. W. A Critical Analysis of the X-Ray Photoelectron Spectra of Ti_3_C_2_T_*z*_ MXenes. Matter 2021, 4, 1224–1251. 10.1016/j.matt.2021.01.015.

[ref31] National Institute of Standards and Technology (NIST). 2012. https://srdata.nist.gov/xps/Default.aspx.

[ref32] Hess A.; Kemnitz E.; Lippitz A.; Unger W. E. S.; Menz D. H. ESCA, XRD, and IR Characterization of Aluminum Oxide, Hydroxyfluoride, and Fluoride Surfaces in Correlation with Their Catalytic Activity in Heterogeneous Halogen Exchange Reactions. J. Catal. 1994, 148, 270–280. 10.1006/jcat.1994.1208.

