# Supplementary material for: Systematic Study of the Multiple Variables Involved in V2AlC Acid-Based Etching Processes, a Key Step in MXene Synthesis
Source: ACS Appl Mater Interfaces. 2023 May 30;15(23):28332–48. doi: 10.1021/acsami.3c01671 (PMC10273183; doi:10.1021/acsami.3c01671)
Supplement: Supplementary file 1 — am3c01671_si_001.pdf [file am3c01671_si_001.pdf]

Supporting Information

A systematic study of the multiple variables involved in V_2AlC acid-based etching processes, a key step on MXene synthesis

Beatriz Mendoza-Sánchez[a,*], Enrique Samperio-Niembro[a], Oleksandr Dolotko[a,b], Thomas Bergfeldt[c], Christian Kübel[d], Michael Knapp[a], and Christopher E. Shuck[e]

[a]*Institute for Applied Materials-Energy Storage Systems (IAM-ESS), Karlsruhe Institute of Technology, Eggenstein-Leopoldshafen, D-76344, Germany*

[b]*Helmholtz Institute Ulm for Electrochemical Energy Storage (HIU), Ulm, 89081, Germany.*

[c]*Institute for Applied Materials- Applied Materials Physics (IAM-AWP), Karlsruhe Institute of Technology, Eggenstein-Leopoldshafen, D-76344, Germany*

[d]*Institute of Nanotechnology and Karlsruhe Nano Micro Facility, Eggenstein-Leopoldshafen, D-76344, Germany*

[e]*A.J. Drexel Nanomaterials Institute and Department of Materials Science and Engineering, Drexel University, Philadelphia, PA 19104, USA*

---

*Corresponding author

Email address: `beatriz.sanchez.wa@gmail.com` (Beatriz Mendoza-Sánchez)

Contents

S1 Experimental methods **S4**

S1.1 Equipment and materials characterisation S4

S1.2 Analytical Methods S4

S2 Particle size studies of V_2AlC samples **S6**

S2.1 Particle classification prior to etching reactions S6

S2.2 SEM images and particle size distribution histograms of A$-V_2AlC$ and B$-V_2AlC$. S7

S3 Synthesis of etched materials - Design of synthesis experiments **S9**

S4 Rietveld refinement of XRD patterns of A-V_2AlC and B-V_2AlC **S11**

S5 V_2AlC chemical composition - determined using analytical and XRD methods **S14**

S6 Further elemental analysis of samples of sets A and B - determined using analytical methods **S15**

S6.1 Elemental analysis of etched samples of sets A in B in mol ratios S15

S6.2 Analysis of O and F of etched materials of sets A and B S15

S6.3 Elemental analysis of a delaminated sample S16

S7 Optical images of products of etching reactions of samples of set A and set B **S17**

S8 Optical images of delamination products of samples of set B **S18**

S9 Additional SEM images of etched samples of sets A and B **S19**

S10 Rietveld refinement of the XRD pattern of the etched B-3-50C-HFHCl sample **S20**

S11 Discussion on the crystal structure of the etched B-3-50C-HFHCl sample **S21**

S12 Discussion of XRD data of thermally treated B-3-50C-HFHCl sample **S23**

S13 XRD data of delaminated material obtained from sample B-3-50C-HFHCl **S25**

S14 X-Ray photoelectron spectroscopy studies of A-V_2AlC and B-V_2AlC precursors **S26**

S15 X-Ray photoelectron spectroscopy studies of etched samples, sets A and B **S29**

S16 X-Ray photoelectron spectroscopy studies of samples of set D **S36**

S1. Experimental methods

S1.1. Equipment and materials characterisation

Centrifugation was performed using a centrifuge (Thermo Scientific Heraeus Multifuge X1R) equipped with a TX-400 rotor (16.8 cm radius). The relative centrifugal force (RCF) is defined as RCF (x g) = $1.118 \times 10^{-5} S^2$ (rpm) x r (cm), in g (gravity acceleration) units, S is the centrifugal rotational speed in rpm, and r is the radius of the rotor in cm. This formula should be used to convert the S (rpm) cited in this work to RCF (x g). The mass of the powders was measured using a microbalance (Sartorius, Germany) with a ± 0.01 mg accuracy.

Scanning electron microscopy (SEM) was performed in a *ZEISS Merlin* (*ZEISS*, Germany) microscope equipped with a GEMINI II column and using the In Lens detector. High-resolution transmission electron microscopy (HRTEM) was performed using a Themis-Z (FEI, USA). X-ray diffraction (XRD) was performed in a STOE STADI P diffractometer with a Mo Kα1 radiation source (= 0.7093 $\AA$, 50 kV, 40 mA), a Ge 111 monochromator and a MYTHEN 1D silicon strip detector. The samples were measured using a *Debye-Scherrer* geometry using glass capillaries (No. 50, 0.5 mm diameter, 0.01 mm thickness). The data was acquired from $2\theta = 2$ to 92 ° in $2\theta = 0.495$ ° steps and acquisition of 1.98 min/step for a total measuring time of 6 h. Each measurement was done twice to ensure reproducibility.

X-ray photoelectron spectroscopy (XPS) was performed in an *Axis Ultra* instrument (Kratos Analytical) equipped with a Delay-line detector (DLD) and a monochromated Al source (300 W, 1486.6 eV). The data was acquired using a hybrid lens mode and charge neutralization was applied. The area of analysis was 700 μm x 300 μm, slot mode was used. Samples were mounted in an insulated state. Data acquisition parameters are specified for each set of samples, sections S14, S15 and S16.

S1.2. Analytical Methods

V and Al were determined by **Inductively Coupled Optical Emission Spectroscopy (ICP-OES)** using an iCAP 7600 DUO (*Thermofisher Scientific*). About 5-10 mg of the sample (accuracy ± 0.05 mg) was dissolved in 10 ml of nitric acid, ultrapurified using the subboiling method, at 523 K for 12 h in the pressure digestion vessel DAB-2 (*Berghof*). The analysis of the elements was accomplished with four different calibration solutions (concentration < 1 mg ml^{-1}) and an internal standard (Sc). Three wavelengths of the elements were used for calculation.

The carbon content was analyzed with a **Carbon-Sulfur analyzer** (*CS 600, LECO*). The carbon concentration was calibrated with two different Fe standards (BAM 19/1326, BAM CrMoNi5/774) and verified with WC (*H.C. Starck*). The calibration range was close to the concentration of the samples. The standards and the samples weighed 4-7 ± 0.03 mg. The samples were placed in ceramic crucibles for measurement. The measurements took place at 100 % power. The evolving gases CO and CO_2 were flushed out by oxygen as a carrier gas and measured by infrared detectors.

The oxygen content was analyzed using the **Carrier Gas Hot Extraction (CGHE)** method. A commercial Hydrogen/Oxygen/Nitrogen analyzer G8 GALILEO (*Bruker AXS*) was used. The oxygen concentration was calibrated with Fe-powder (KED 1025, *ALPHA*) and was verified with another carbide silicon standard (BAM S008) and boron nitrate standard (ERM-ED103-BN). The calibration range was close to the concentration of the samples. The standards and the samples weighed 0.7-12 ± 0.03 mg and were placed in Sn crucibles (5-12 mm). All the samples in the Sn crucibles were then placed in a Ni crucible with 500 mg gravel. The Ni crucible was then placed into an outgassed (> 1000 A) high temperature graphite crucible graphite. The measurements took place at about 1000 A. The evolving gas CO was flushed out by helium as an inert carrier gas and measured by an infrared detector.

The fluorine content, in the digestion samples used for determination of V and Al, was analyzed using a **Combination Fluorine Electrode** (*perfectION*, *Mettler Toledo*). The digestion samples were diluted and adjusted to a pH value between 5 and 8 using a 5 mol L^{-1} NaOH solution and a total ionic strength adjustment buffer (TISAB) solution. The fluorine concentration was measured with the method of known addition (one addition).

Chemical analysis methods reported the % mass of elements of each precursor(A− or B−V_2AlC) and each of the etched samples. A mass balance allowed to determine % $m_{f,n}$ = ($m_{f,n}$ / $m_{i,n}$) x 100, where $m_{f,n}$ is the mass of an element n (V,Al,C,O,F) remaining after etching and $m_{i,n}$ is the initial mass of element n, i.e. its mass in the corresponding precursor.

S2. Particle size studies of V_2AlC samples

S2.1. Particle classification prior to etching reactions

Generally, V_2AlC samples were sieved a first time after synthesis procedures (using sieve 1), and, a second time, prior to etching procedures (using sieve 2). In this last step the mass fractions of below and above 36 μm were determined and, generally, the fraction < 36 μm was selected to undergo etching procedures. Variations of particle size distribution across samples reflects variations on milling procedures after synthesis (see main text).

**Sample**	**Sieve 1 (μm)**	**Sieve 2 (μm)**	**f_1 < 36 μm (wt. %)**	**f_2 > 36 μm (wt. %)**	**Etched f**
**A-V_2AlC**	40	36	94.8	5.2	1+2
**B-V_2AlC**	-	36	65	35	1
**C-V_2AlC**	71	36	82.7	17.3	1
**D-V_2AlC**	40	36	100.0	0.0	1

Table S1: Particle size selection of V_2AlC samples. Here f = fraction. The last column indicates the selected fraction used for etching procedures.

Why particle size classification? Because etching of the A element in a MAX is a solid-liquid reaction, the surface area plays a key role on etching processes. Etching will increase efficiency as particle size decreases, i.e. as surface area increases. Reaction time and temperature are a function of particle size/surface, and, thus, optimization can be done for a hypothetical monodisperse MAX sample of particle size p_m. In practice, in a size polydisperse MAX sample, particles of size < p_m will undergo overetching, and particles of size > p_m will undergo underetching. The former, generally, leads to poor etching reaction yields, and the latter to poor or no delamination. Thus, in order to mitigate effects of particles size on etching processes, MAX particle classification was considered in this study.

S2.2. SEM images and particle size distribution histograms of $A-V_2AlC$ and $B-V_2AlC$

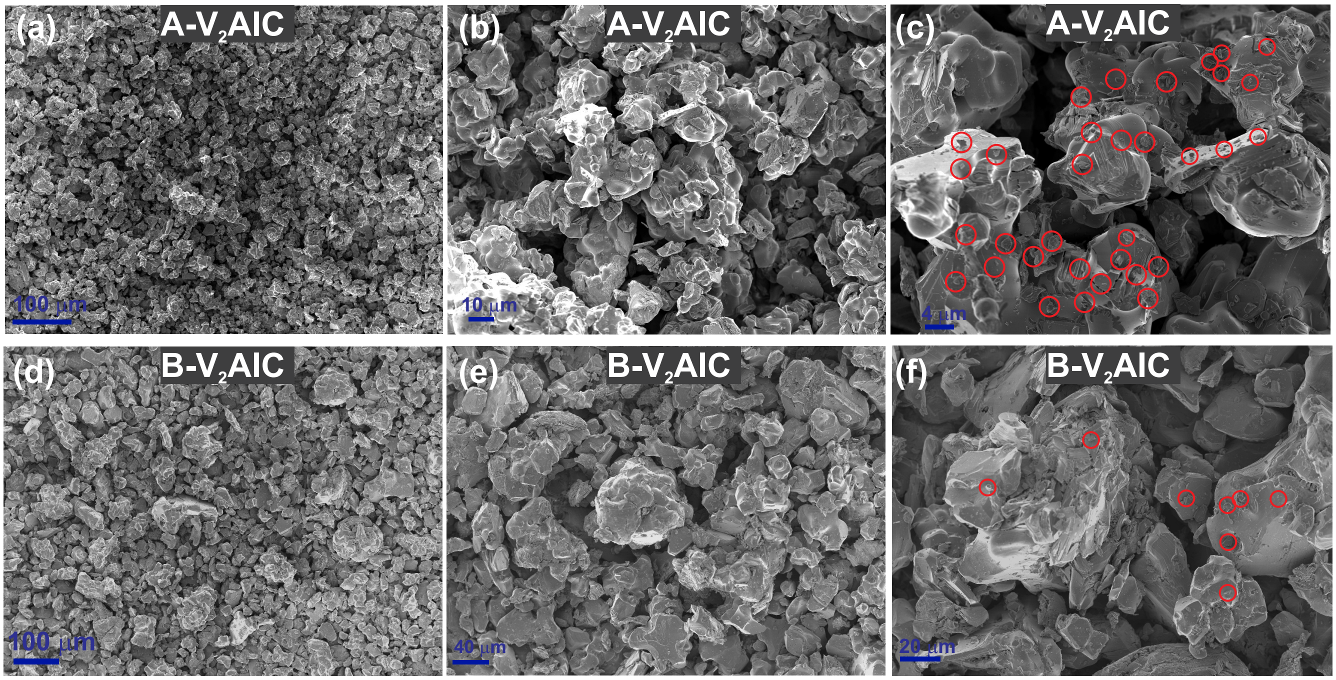

Figure S1: SEM images of MAX phases: (a-c) $A-V_2AlC$ (after first sieving using a 40 μm sieve) and (b-d) $B-V_2AlC$. Red circles in (c) and (f) indicate particles of size below 2-4 μm.

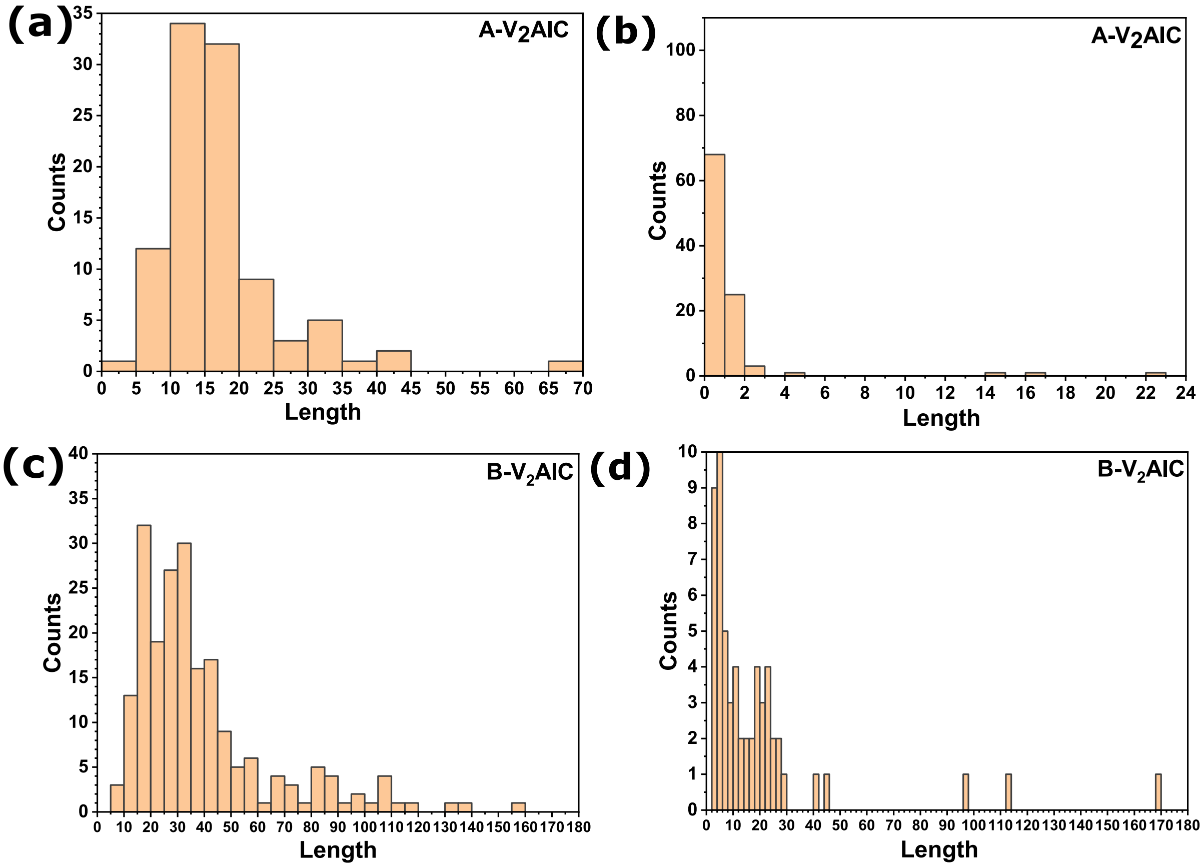

Figure S2: Particle size histograms of (a-b) A−V_2AlC (after first sieving using a 40 μm sieve) and (c-d) B−V_2AlC (no sieving). (a) Counting over image S1b, (b) Counting over image S1c, (c) Counting over image S1d and (d) Counting over image S1e. Particle aggregates were counted as a unit. In (b-d), small particles (1-6 μm, indicated with red circles in Figures S1c,f) lying on top of selected larger particles (outliers of the histogram) were counted. If a larger sample is to be considered, counting of the small particles over the entire number of large particles should be scaled up.

S3. Synthesis of etched materials - Design of synthesis experiments

A design of experiments for the synthesis of etched materials departed from a preliminary partial optimization of reaction time and temperature, which were selected based on the sole criteria of etched materials leading or not to delamination (Table S2).

**Set A, A-V_2AlC(1 g)**						
Sample	Acid (ml)	Vessel size	T (°C)	t (d)	MR (rpm)	Delamination
A'1	HF(20)	L	45	3	400	no
A'2	HF(20)	L	45	4	400	no
A'3	HF (20)	L	45	5	400	yes
**Set B, B-V_2AlC(1 g)**						
B'1	HF(12)/HCl(8)	L	40	3	400	no
B'2	HF(12)/HCl(8)	L	40	4	400	no
B'3	HF(12)/HCl(8)	L	40	5	400	yes

Table S2: Synthesis conditions selection- preliminary experiments. T = temperature, t = time, L = Large, MR = Mixing rate. All samples underwent delamination reaction; the notes "no" or "yes" makes reference to whether delaminated product was obtained. HF is 48 wt. % and HCl is 37 wt.%.

For each precursor A$-V_2AlC$ and B$-V_2AlC$, a minimum reference temperature was used for the etching reaction (as per previous findings), 45 °C and 40 °C, respectively. Then, reaction times were tested (Table S2). The minimum reaction time leading to delamination was selected as the optimised reaction time.

For set A, using A$-V_2AlC$, and etching in HF (20 ml), the optimised reaction time was 4 days. For the main investigation, temperatures 10 °C below and above the reference temperature of 45 °C were considered, keeping all other synthesis conditions the same (Table 1). In a fourth sample, a different combination of acids was used, HF 48 wt.% (12 ml)/HCl 37 wt.% (8 ml). In a fifth sample, mixing (stirring with a magnetic bar) was set to 100 rpm (poor mixing), instead of 400 rpm used for the other samples (Table 1).

For set B, using B$-V_2AlC$, and etching in HF 48 wt.% (12 ml)/HCl 37 wt.% (8 ml), the optimised reaction time was 5 days. For the main investigation, a temperature 10 °C above the reference temperature of 40 °C was considered (Table 1). All samples of set B were mixed at the

same rate of 400 rpm.

Sets C and D are complementary samples for further XRD or XPS analysis and synthesis conditions are reported in Table 1.

Dimensions of reaction vessel/stir bar. In most experiments of sets A to D, the same type of reaction vessel (250 ml, 5 cm diameter, named L = large) and stir magnetic bar (2 cm length x 9 mm diameter) were used. In selected experiments of set A (Table 1), a small PTFE reaction vessel was used (60 ml, 2.0 cm diameter, named S= small) and the same stirring bar were used.

S4. Rietveld refinement of XRD patterns of A-V_2AlC and B-V_2AlC

The XRD patterns of the MAX precursors A-V_2AlC and B-V_2AlC were analysed using Rietveld refinement (RR) (Figures S3 and S4). The *FullProf* software package was utilised [1]. The background of all the diffraction patterns was fitted using a linear interpolation between selected data points in regions with no reflections present. The Thompson-Cox-Hastings pseudo-Voigt function was used for the reflection profile shape description. In general, scale factor, zero angular shift and lattice parameters were allowed to vary during refinement. A prior refinement of a LaB_6 standard allowed the determination of the U,V,W (*Caglioti*) parameters [2], which constituted the instrumental resolution function used for the refinement of the XRD patterns of the samples. For the V_2AlC phase, the atom coordinate z of the V atom was allowed to vary. For some phases, the overall isotropic displacement (temperature) parameter and additional profile parameters were allowed to vary during refinement. Preferred orientation along the [001] direction was taken into account for the V_2AlC phase.

For the A-V_2AlC, a main phase was identified as V_2AlC (P 6_3/m m c space group (194)). Other 3 secondary phases were identified. The first was V_4AlC_3 (P 6_3/m m c space group (194). CIF files for V_2AlC and V_4AlC_3 were generated using as guidance previous reports [3, 4] but mostly using our own raw data. The second phase was Al_2O_3 (R -3 c space group, ICSD 9770). The third minor phase was not identified and had very low intensity reflections at 2θ = 15.05 °, 16.87 °, and 19.73 °. The refined lattice parameters and the weight fraction of each phase are reported in Table S3.

**Phase**	**Space group**	**a** (*Å*)	**b** (*Å*)	**c** (*Å*)	**wt.%**	$\mathbf{R}_p$	$\mathbf{R}_{wp}$	χ^2
V_2AlC	P 6_3/m m c	2.91646(2)	2.91646(2)	13.13175(3)	91.89(0.24)	3.1	4.21	3.37
V_4AlC_3	P 6_3/m m c	2.92697(1)	2.92697(1)	22.68779(2)	6.52(0.07)			
Al_2O_3	R -3 c	4.75482(4)	4.75482(4)	13.004(1)	1.59(0.07)			

Table S3: Structural parameters of the phases found in the A-V_2AlC sample. For all phases, $\alpha = \beta = 90$ °, γ = 120 °. Numbers in brackets give statistical deviations for the last significant digit. Standard deviations have been multiplied by the Bérar factor to correct for local correlations. The profile R-factor (R_p), the weight profile R-factor(R_{wp}) and χ^2 residuals are reported.

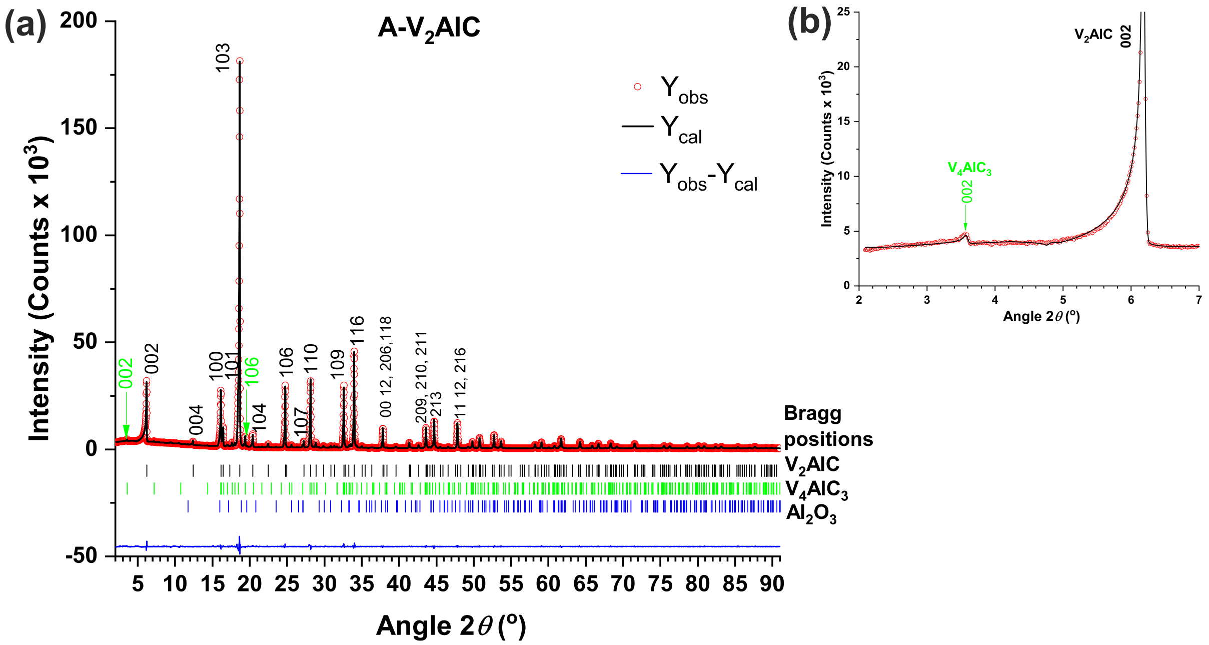

Figure S3: (a) XRD pattern of A-V_2AlC and structural model calculated using Rietveld refinement and (b) inset showing the 002 reflection of V_4AlC_3. The main reflections of the main phase V_2AlC are indicated (black labels) as well as some of the V_4AlC_3 secondary phase (green labels).

For the B-V_2AlC, a main phase was identified as V_2AlC ($P6_3/m$ m c space group (194)). A secondary phase present was identified as Al_2O_3 (R -3 c space group, ICSD 9770). The refined lattice parameters and the weight fraction of each phase are reported in Table S4.

**Phase**	**Space group**	**a** (Å)	**b** (Å)	**c** (Å)	**wt.%**	$\mathbf{R}_p$	$\mathbf{R}_{wp}$	χ^2
V_2AlC	P 6_3/m m c	2.91531(2)	2.91531(2)	13.14814(6)	97.32(0.37)	3.04	4.36	3.66
Al_2O_3	R -3 c	4.75977(6)	4.75977(6)	12.98661(2)	2.68(0.26)			

Table S4: Structural parameters of the phases found in the B-V_2AlC sample. For all phases, $\alpha = \beta = 90$ °, $\gamma =$ 120 °. Numbers in brackets give statistical deviations for the last significant digit. Standard deviations have been multiplied by the Bérar factor to correct for local correlations [5]. The profile R-factor (R_p), the weight profile R-factor(R_{wp}) and χ^2 residuals are reported.

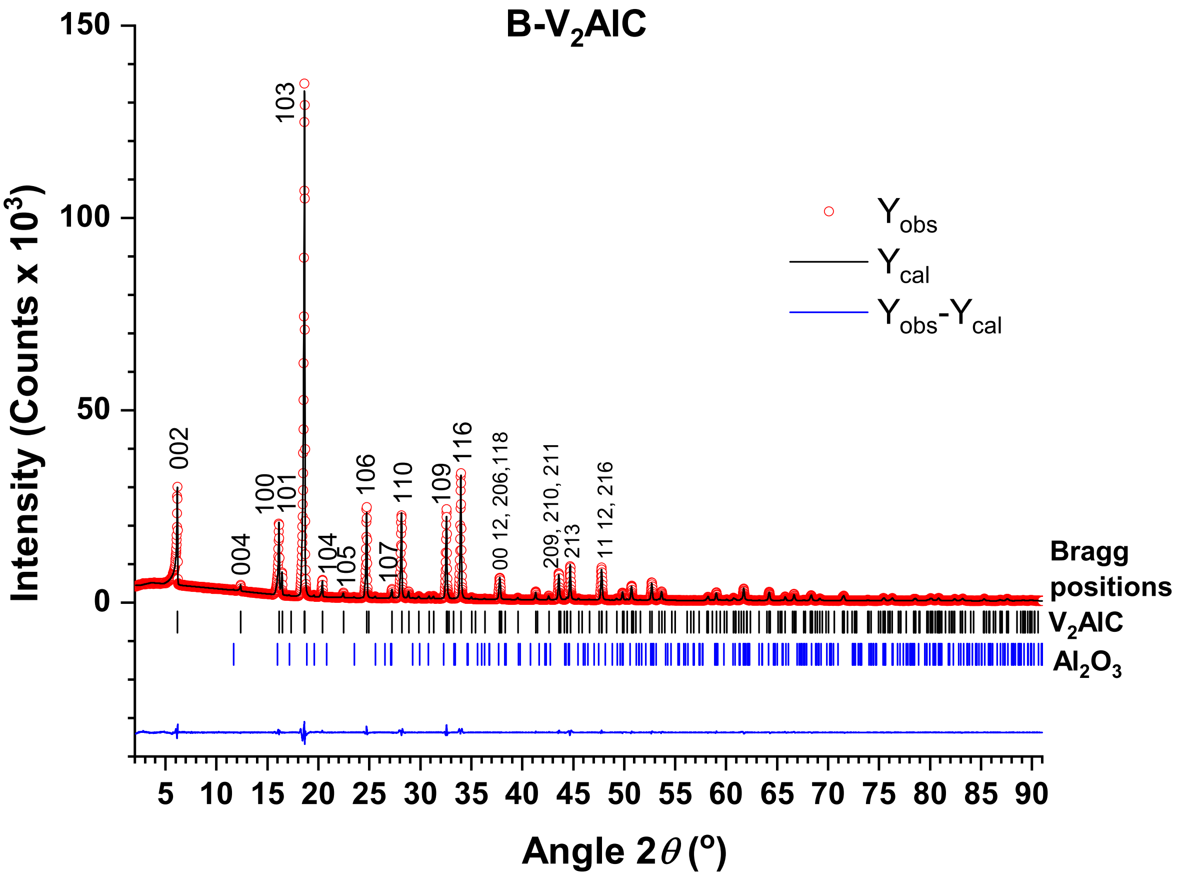

Figure S4: XRD pattern of B-V_2AlC and structural model calculated using Rietveld refinement. The main reflections of the main phase V_2AlC are indicated (black labels).

S5. V_2AlC chemical composition - determined using analytical and XRD methods

**A-V_2AlC**			
**Element**	**wt.% (total)**[a]	**wt. % (in V_2AlC)**[b]	**2 x (mol/mol V)**[c]
**V**	70.54 ± 0.08	93.02	2.00 ± 0.02
**Al**	18.21 ± 0.04	92.14	0.96 ± 0.007
**C**	8.67 ± 0.06	89.89	1.00 ± 0.008
**O**[d]	1.02 ± 0.22	-	0.09 ± 0.0007
**Unknown**	1.56		
**Total**	100.00		
**B-V_2AlC**			
**V**	70.40 ± 0.2	100	2.00 ± 0.02
**Al**	18.30 ± 0.1	92.92	0.91 ± 0.007
**C**	8.32 ± 0.06	100	1.00 ± 0.008
**O**[d]	1.09 ± 0.08	–	0.09 ± 0.0007
**Unknown**	1.89		
**Total**	100		

Table S5: Elemental composition of A-V_2AlC and B-V_2AlC. (a) This is a weight percentage of elements per total mass of the etched powder. (b) This is the weight percentage of each element in the V_2AlC phase per total mass of the respective element in all phases - as calculated from percentages of phases reported by XRD studies (Tables S3 and S4). (c) The ratio (mol/mol V) x 2 considers only the V_2AlC phase and excludes secondary phases.(d) The quantified O is the total in the etched sample (main and secondary phases).

Note 1: Calculating mol ratios respect a certain element assumes chemical stability during synthesis of such element. It is known that amongst the three elements in this MAX phase, Al is the element that could be evaporated during the solid state synthesis.Therefore, normalisation respect to Al was not considered. About V and C, it is known that secondary phases such as VC can form during synthesis, yet V and C are not expected to be lost. Normalisation respect C, would report a deficiency in V (< 2), which is not likely, thus, normalisation to V was considered.

Note 2: A–V_2AlC and B–V_2AlC samples had similar O contents. This O content consist of surface O on V_2AlC particles, as revealed by XPS studies (Section S14), most likely also surface O on the V_4AlC_3 secondary phase, and O in Al_2O_3, as revealed by XRD studies (Section S4). Surface O content is expected as per intrinsic reactivity of V at a standard atmosphere environment. The precursors were stored in a glove box before etching procedures but this oxidation most probably has place right after MAX synthesis, upon withdrawal of samples from tube furnaces and during milling procedures, performed in standard atmosphere.

S6. Further elemental analysis of samples of sets A and B - determined using analytical methods

S6.1. Elemental analysis of etched samples of sets A in B in mol ratios

The elemental analysis of etched samples is shown in (mol i/mol C) ratio , where i = V, Al, C, O, for an alternative visualization of the variations of V and Al versus the precursor MAX. It must be noted that as the analytical methods determine bulk elemental composition, this cannot be interpreted as the stoichiometry of a "compound" in heterogeneous samples.

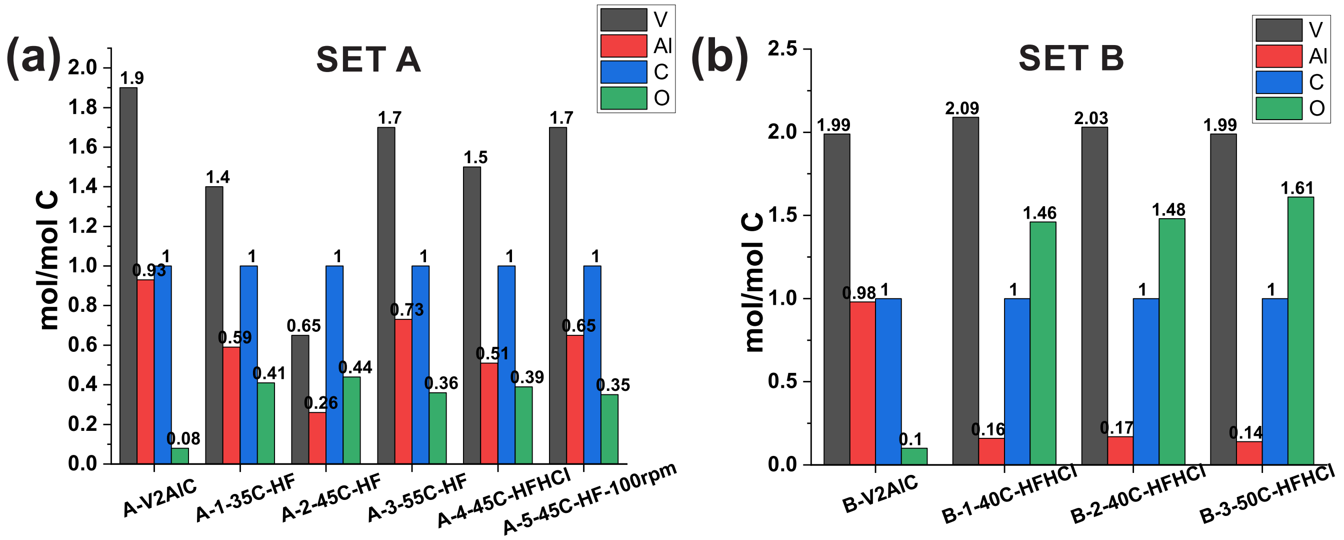

Figure S5: Element analysis of samples of (a) set A and (b) set B in mol ratios.

S6.2. Analysis of O and F of etched materials of sets A and B

Regarding **O content**, it is clear that samples of set B had a larger mass content of O (979-1113 % mass) than set A (213-330 %) (Figure 1a,b). Etching procedures are performed in standard atmosphere, thus oxidation is expected to occur. Then, etching procedures are followed by washing and vacuum-assisted filtration procedures, also performed in standard atmosphere. This is enough time (hours) for oxidation to occur before samples are spared in a glove box.These conditions are the same for samples of set A and B. Thus, differences in the oxidation degree of set B vs set A, can be explained rather by the waiting time between synthesis and O analytical determination. Samples of set A were seating between 15-31 days before analysis, whereas samples of set B sat for 97 days. Even though the samples were spared in a glove box with an O_2 content < 0.1 ppm, the oxidation was imminent. This gives an idea of the effects of aging, even in nearly inert atmospheres, of these type of samples.

Regarding **F content**, set A had a higher F mass content for samples etched with a HF/HCl mix as compared to samples etched in HF only (Figure S6). The F mass content was also higher for samples of set B (up to 8.6 %), all etched in HF/HCl as compared to set A (3.9 %). This indicated that the acid mix promotes a better fluorination of the MXene surface.

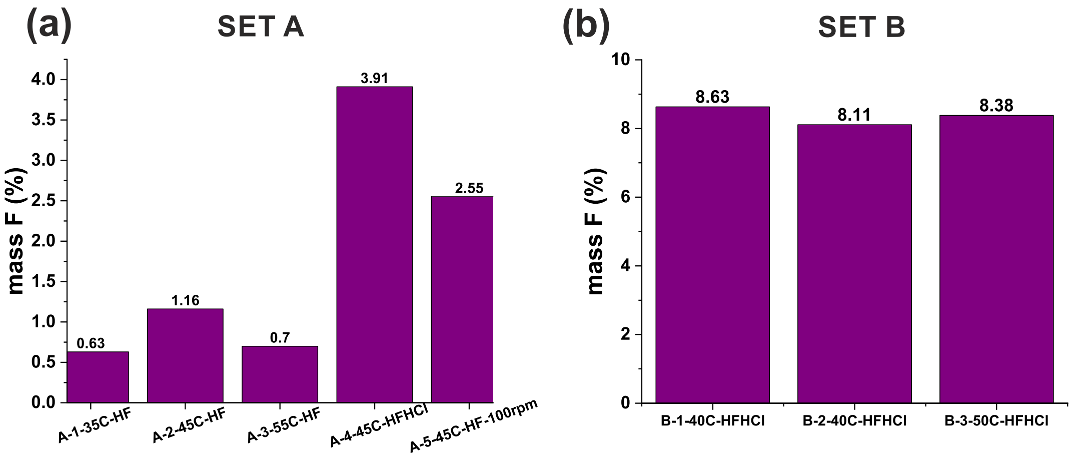

Figure S6: Mass element analysis of F for samples of (a) set A and (b) set B.

S6.3. Elemental analysis of a delaminated sample

In set B, sample B-1-40C-HFHCl was delaminated to obtain a B-1-40C-HFHCl-DEL sample (Figure S9). A film was obtained by vacuum-assisted filtration and analysed (Figure S9c). The mass content of elements were: V (54.40 ± 0.3 wt.%), Al (0.02 wt.%), C (14.40 ± 0.6 wt.%), O (16.70 ± 0.5 wt.%) and F (7.3 ± 0.15 wt.%). This confirmed that upon delamination, all the Al is indeed completely etched away. Then, the mol ratio of V:C was 2:2.25. The excess of C, expected 2:1 for V_2C, could be explained on the basis of residual carbon coming from the TBAOH molecule used for delamination. O content, as for the etched material, is always present as per the high susceptibility to oxidation of this MXene.

S7. Optical images of products of etching reactions of samples of set A and set B

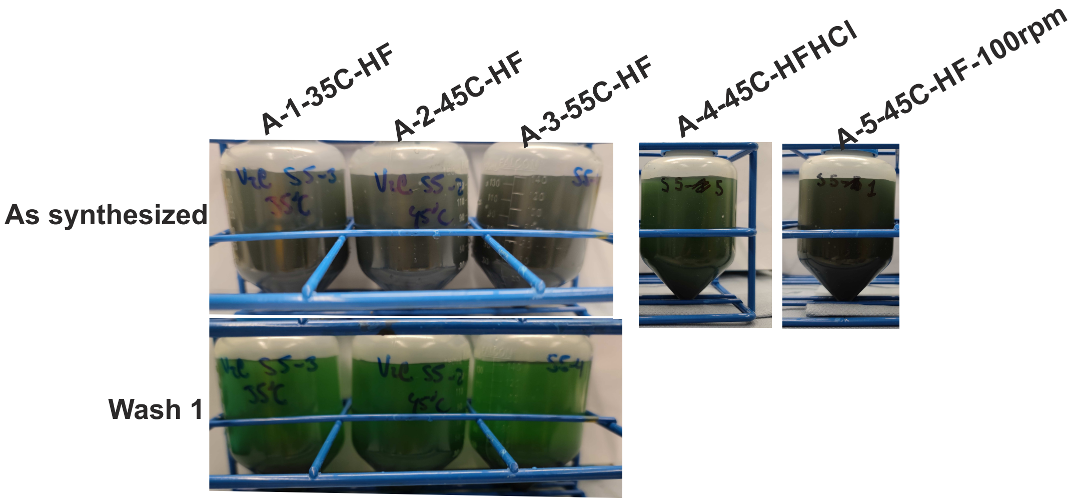

Figure S7: Optical images of the product of etching reactions according to reaction conditions of set A (Table 1 in the main text). The top row displays products just after synthesis where DI water was added for washing. The bottom row displays the products after the first centrifugation step of 5000 rpm for 10 min.

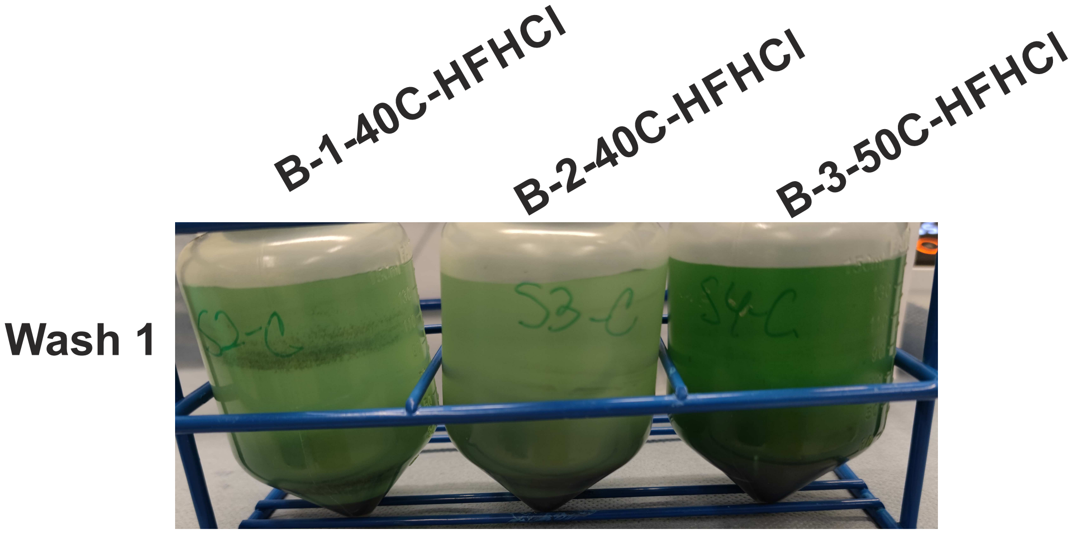

Figure S8: Optical images of the product of etching reactions according to reaction conditions of set B (Table 1 in the main text), after the first centrifugation step of 5000 rpm for 10 min.

S8. Optical images of delamination products of samples of set B

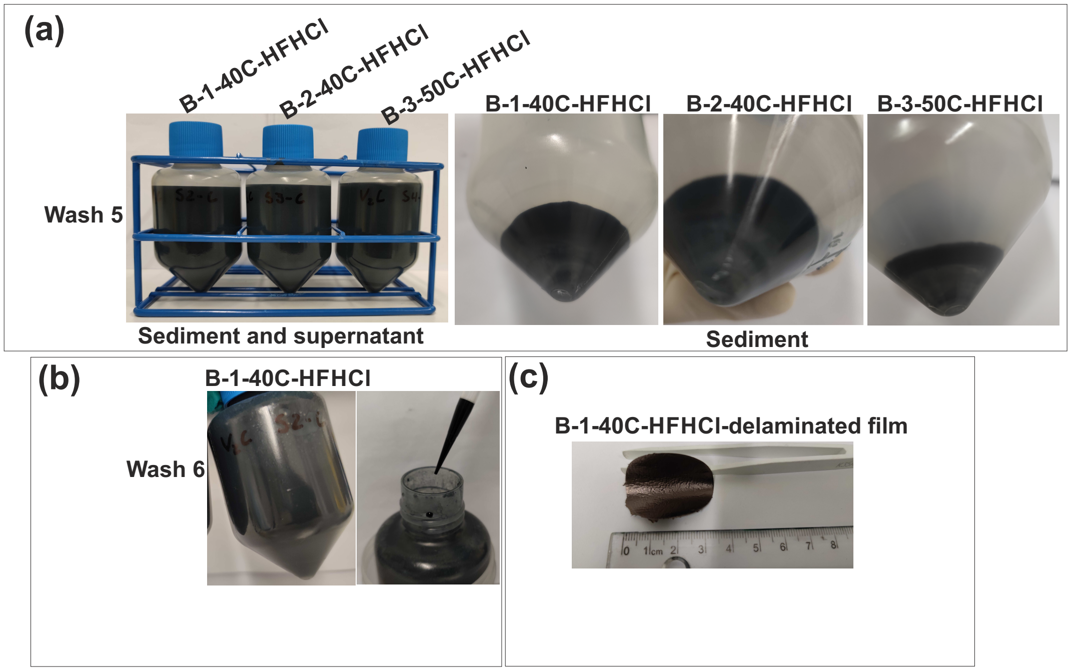

Figure S9: Optical images of delamination products corresponding to etched samples of set B: B-1-40C-HFHCl, B-2-40C-HFHCl and B-3-50C-HFHCl.(a) Delamination products after the 5th washing step consisting of vigorous mixing and centrifugation at 3500 rpm for 30 min - delaminated products are obtained from the 4th washing step onwards. The delamination products are shown before (left) and after (right) pipetting out the supernatant (right).(b) Delamination product of the samples B-1-40C-HFHCl after the 6th wash, the as pipetted out supernatant is shown. (c) A film obtained by vacuum-assisted filtration of supernatant of sample B-1-40C-HFHCl.

S9. Additional SEM images of etched samples of sets A and B

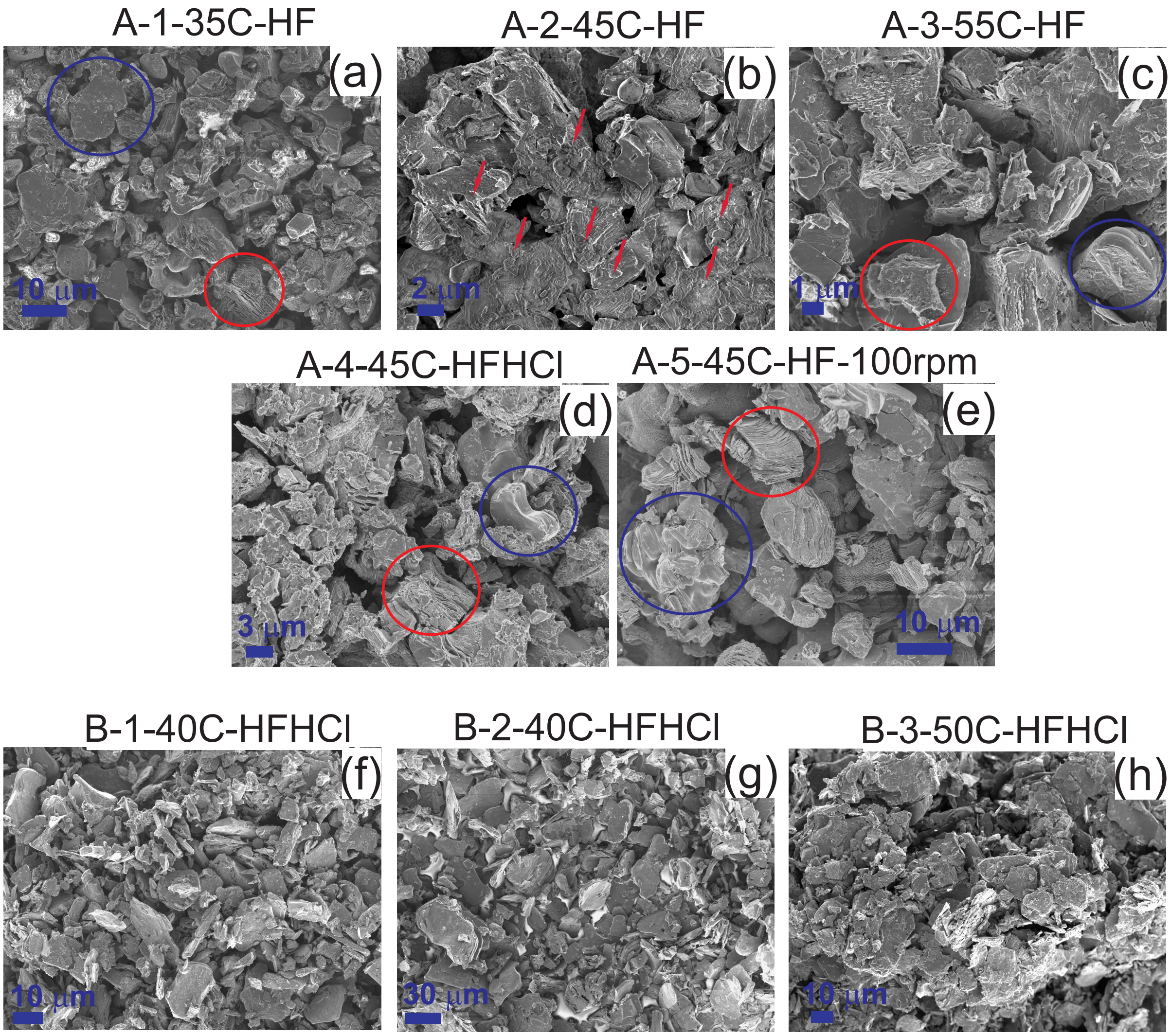

Figure S10: SEM images of etched samples of (a-e) set A and (f-h)set B. Red circles indicate etched particles, blue circles indicate poorly or non etched particles, arrows indicate very small particles.

S10. Rietveld refinement of the XRD pattern of the etched B-3-50C-HFHCl sample

The XRD data of the etched B-3-50C-HFHCl sample was analysed (Figure S11). An *ab initio* semi-exhaustive trial-and-error method (*TREOR* software [6]) was used to determine indexation of the Bragg reflections that were best fitted when using an hexagonal symmetry for the etched V_2AlC phase. Subsequently, the Le Bail refinement method was used to verify this solution (using *FullProf* software[1]) and to determine the space group of the etched V_2AlC phase while taking into account the presence of secondary phases. The space group of the etched V_2AlC phase was determined as P 6_3/m m c. The final solution determined three phases: etched V_2AlC phase, unetched V_2AlC and Al_2O_3. The determined lattice parameters are summarized in Table S6.

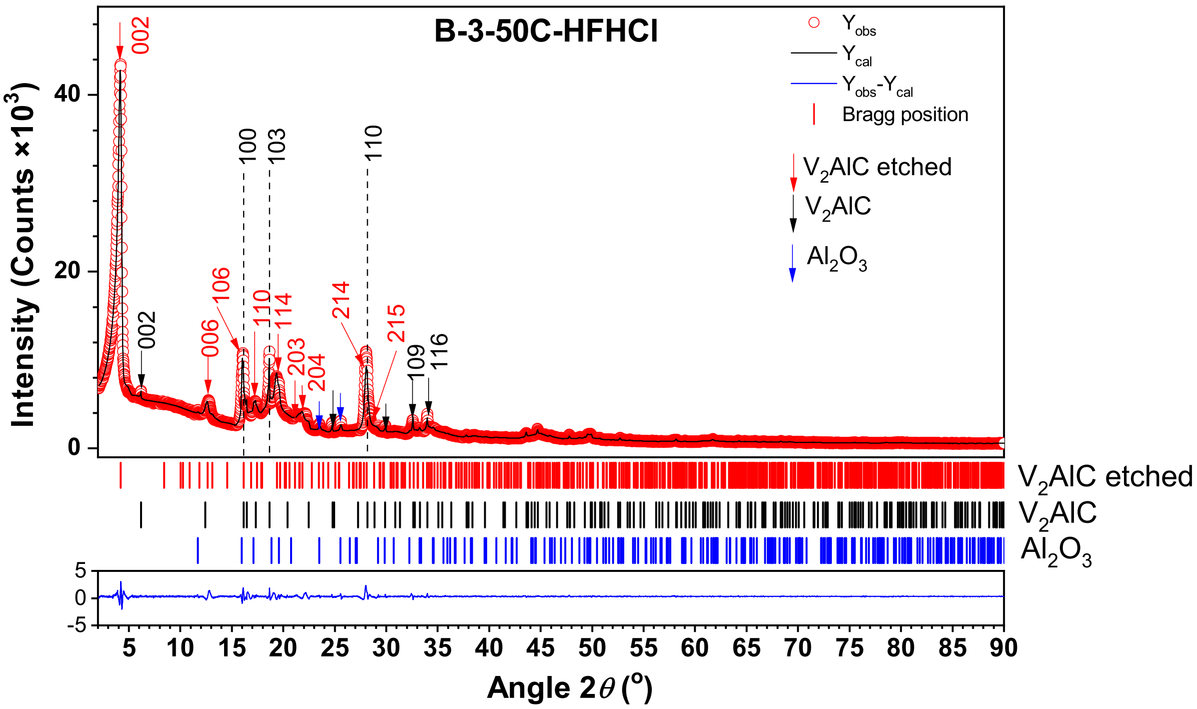

Figure S11: XRD pattern of the etched B-3-50C-HFHCl sample and model calculated using the Le Bail method.

Phase	Space group	a ($\AA$)	b ($\AA$)	c ($\AA$)	R_p	R_{wp}	χ^2
V_2AlC etched	P 6_3/m m c	4.6836(3)	4.6836(3)	19.3377(4)	3.13	4.8	5.31
V_2AlC	P 6_3/m m c	2.9156(4)	2.9156(4)	13.1362(4)			
Al_2O_3	R -3 c	4.7699(2)	4.7699(2)	12.9995(6)			

Table S6: Lattice parameters of the phases determined for the etched B-3-50C-HFHCl sample. For all phases, $\alpha = \beta = 90$ °, $\gamma = 120$ °. Numbers in brackets give statistical deviations for the last significant digit. Standard deviations have been multiplied by the Bérar factor to correct for local correlations [5]. The profile R-factor (R_p), the weight profile R-factor(R_{wp}) and χ^2 residuals are reported.

S11. Discussion on the crystal structure of the etched B-3-50C-HFHCl sample

A refinement of the XRD data of the etched B-3-50C-HFHCl sample was done using the Le Bail method (section S10, Figure S11) and revealed the presence of at least 3 phases: unetched B-V_2AlC, Al_2O_3, and etched V_2AlC phase (Table S6). The XRD intensity signals for the etched V_2AlC phase, most likely have contributions from fractions with different degrees of etching and of delaminated V_2C. This was not taken into account in the refinement. Therefore, the solution provided by the refinement is considered an approximation. Here, experimental data is discussed only taking into account the identified phases but not the reflection assignation of the etched V_2AlC phase.

The XRD patterns of B-V_2AlC and etched B-3-50C-HFHCl samples are shown in Figure S12. The presence of the precursor B-V_2AlC was evidenced by the presence of the corresponding 002 reflection (Figure S12b). This reflection has been broadly discussed in the main text. Here, a discussion about other relevant structural aspects at higher 2θ angles is addressed.

First, upon etching h0l reflections (describing transversal 2D planes cutting the c-axis), i.e. 101, 103, 106 and 109, and 116 (describing 3D planes) became less intense and/or broadened, which indicated the transition of a 3D to a 2D structure (Figure S12a). Other minor h0l reflections, such as 104, 105 and 107, almost vanished. Upon delamination all the h0l 3D structure turned into a broad intensity or vanished (Figure S14) indicating a successful 3D to a 2D transformation and interlayer ordering changes [7].

Second, some broad intensities emerged. A broad signal appeared at slightly lower 2θ angles than the 100 reflection of the B-V_2AlC precursor (3) (Figure S12c). The broadening and shift of this signal was more enhanced for samples of set C, at $2\theta = 15.92$ ° (Figure 6c). This signal must have contributions of the reflection 100 of the B-V_2AlC precursor and the newly emerging etched V_2AlC phase. The shift to lower angles described a larger a lattice parameter of the etched V_2AlC phase. For the A-V_2AlC, one should keep in mind the presence of Al_2O_3 as secondary phase. In this case, the 104 reflection of Al_2O_3 should have a contribution to the broadening of this signal. This is more obvious in the overetched samples (Figure 8b).

A similar intensity broadening was observed around the 110 reflection of the B-V_2AlC precursor (Figure S12d). This signal seemed to have contributions from two reflections (9 and 10) at $2\theta = 28.04$ ° and $2\theta = 28.34$ °. This was, again, more enhanced for set C, where new intensities emerged at slightly different angles, $2\theta = 27.72$ ° and $2\theta = 28.31$ ° (Figure 6d). This signal must have contribution from the the B-V_2AlC precursor and the emerging

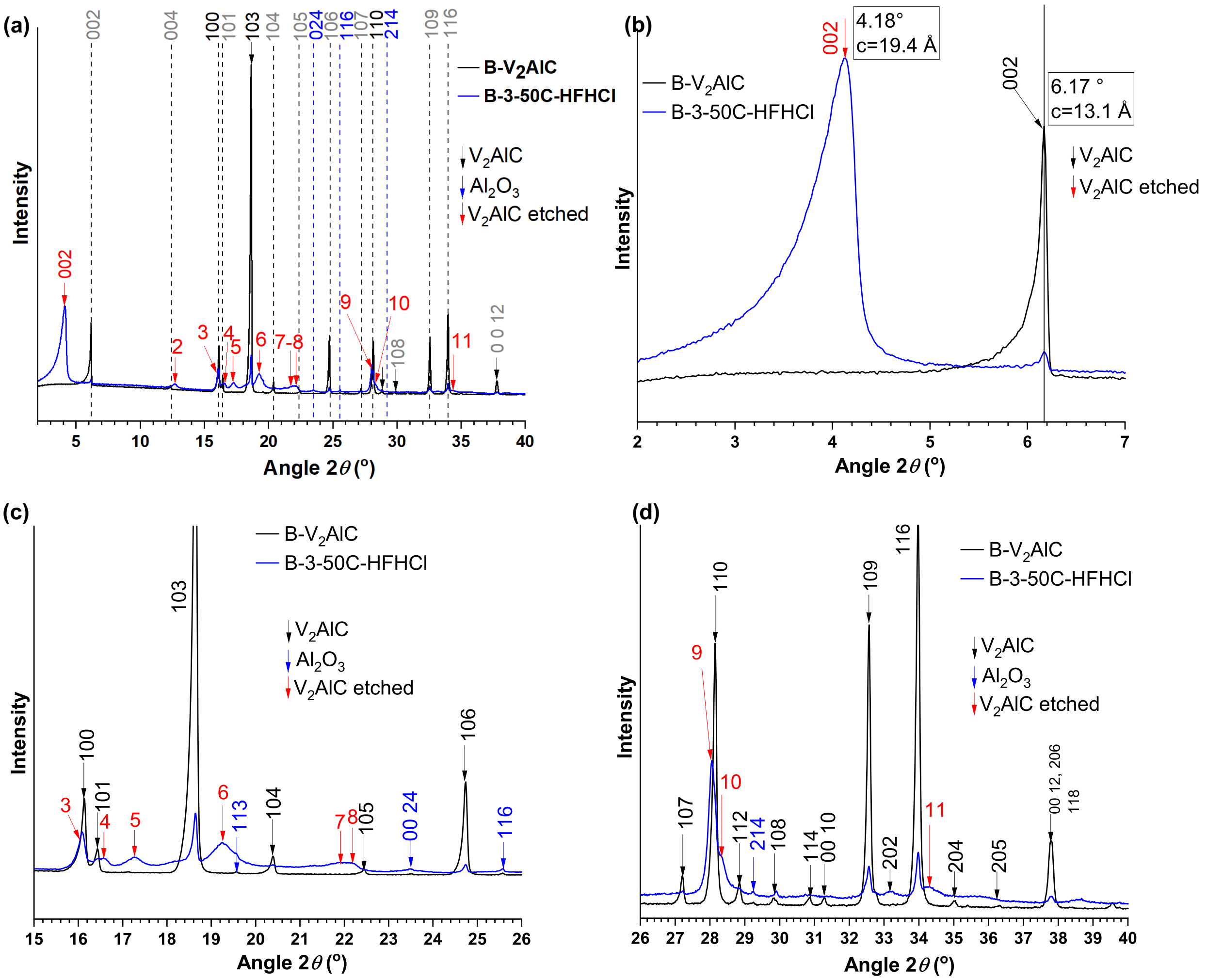

Figure S12: (a) XRD pattern of sample B-3-50C-HFHCl and (b-d) high magnification at specific 2θ ranges. The XRD pattern of B-V_2AlC is added as reference. In (a) labels of reflections of the V_2AlC phase "present" (2θ shifted in most cases) in the etched B-3-50C-HFHCl are coloured in black (if of high intensity in the etched B-3-50C-HFHCl sample) and gray (if of low or very low intensity in the etched B-3-50C-HFHCl sample).

etched V_2AlC phase. The shift to lower angles of this signal, again, described larger a=b lattice parameters of the etched V_2AlC phase.

Comparison of XRD patterns of precursor B-V_2AlC, etched material B-1-40C-HFHCl and the corresponding B-1-40C-HFHClDELAMINATED delaminated material (Figure S14), confirmed that upon delamination, reflections 3 and 9 are still present and further shifted to lower angles respect the corresponding 100 and 110 reflections of B-V_2AlC. This is indicative of a continuous crystal evolution as the number of crystal layers decreased upon etching and delamination processes. Equivalently, this indicated that reflections 3 and 9 are the result of crystal transformations of reflections 100 and 110 of the precursor. Shift of the reflections indicated expansion of lattice parameters a and b; and a mild broadening implied other crystal transformations [7]. In other 2D materials, such as MoS_2, reflections 100 and 110, are present in monolayer material, and, thus, these reflections are not necessarily

indicative of few-layers material [7].

Other new intensities emerged in the etched V_2AlC phase at $2\theta = 16.53$ ° (4), 17.92 ° (5), 19.37 ° (6), a broad signal with no well defined reflections between $2\theta = 20.96$ ° and $2\theta = 22.63$ ° (7-8), and a small broad signal at 34.29 ° (11) (Figure S12c-d). Since these intensities are definitely not present in the precursor B-V_2AlC, taking into account main and the secondary phase Al_2O_3, they belong to the new etched phase. A further insight into the crystal structure of the etched phase was given by thermal treatment of samples.

S12. Discussion of XRD data of thermally treated B-3-50C-HFHCl sample

The etched sample B-3-50C-HFHCl was thermally treated in Ar atmosphere for 5 hours at 200 °C (Figure S13). In a second experiment, thermal treatment was done in two stages, a first one of at 120 °C for 3 h and a second one at 450 °C for 5 h (Figure S13).

Upon thermal treatment at 200 °C and 450 °C, the main h0l reflections 101, 103, 106, 109 and 116, became more defined indicating a re-establishment of the 3D structure of the B-V_2AlC precursor. No shift was observed, indicating that corresponding planes are not involved in molecule intercalation and that are structurally stable up to 450°C, as expected given their origin in the B-V_2AlC precursor prepared at 1550 °C. Some reflections of the secondary phase Al_2O_3 became more defined.

At 200 °C, the intensities 3 and 9 slightly shifted further down to lower angles, respect to reflections 100 and 110 of the B-V_2AlC precursor, indicating further expansion of the a and b lattice parameters. At 450 °C, major structural transformations took place around reflection 3 that does not let to establish clearly an evolution. In the case of the reflection 9, it shifted back to the same position than the reflection 110 of the B-V_2AlC precursor. These findings, further indicate that reflections 3 and 9, are indeed structural transformation derived from reflections 100 and 110 of the B-V_2AlC precursor.

At 200 °C, intensity 4 vanished and 5 shifted to higher angles. At 450 °C, intensities 3-5 seemed to have merged in a broad intensity, indicating the collapse of the crystal structures involved [8]. At 200 °C and 450 °C, intensity 6 shifted to higher angles and became more defined, indicating reduction of interplanar distances and an improved crystallinity. This indicated that reflection 6 described a structural component of the etched phase. At 200 °C, intensities 7-8 vanished, but at 450 °C, a new intensity (12) appeared instead. In summary, at 450 °C major structural changes occurred, affecting greatly the structure described by the new reflections of the etched phase.

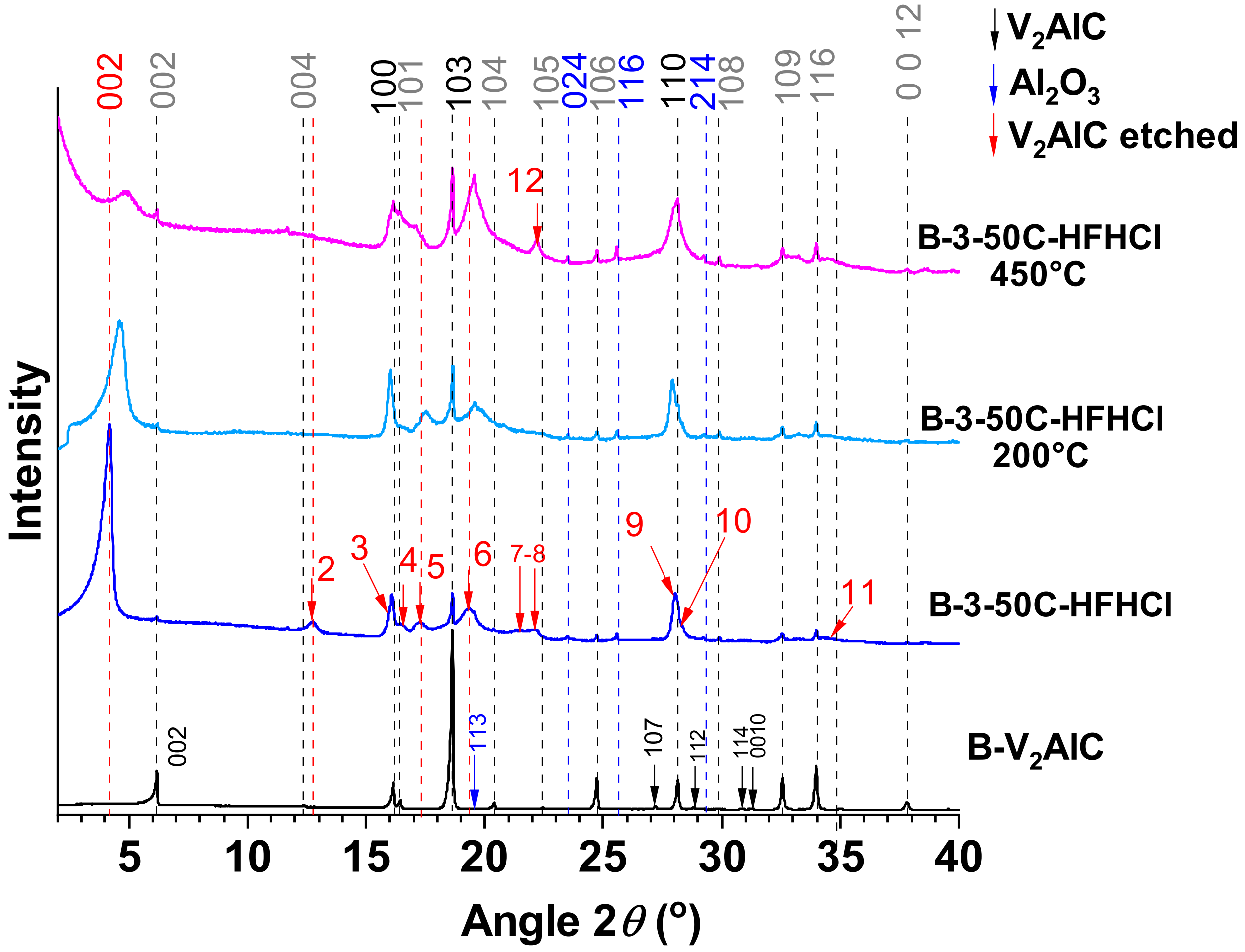

Figure S13: XRD patterns of pristine B-3-50C-HFHCl sample and after thermal treatment in Ar atmosphere. The XRD pattern of the precursor B-V_2AlC is added for reference. Labels of reflections of the V_2AlC phase “present” (2θ shifted in most cases) in the etched samples are coloured in black (if of high intensity in the etched sample) and gray (if of low or very low intensity in the etched sample).

S13. XRD data of delaminated material obtained from sample B-3-50C-HFHCl

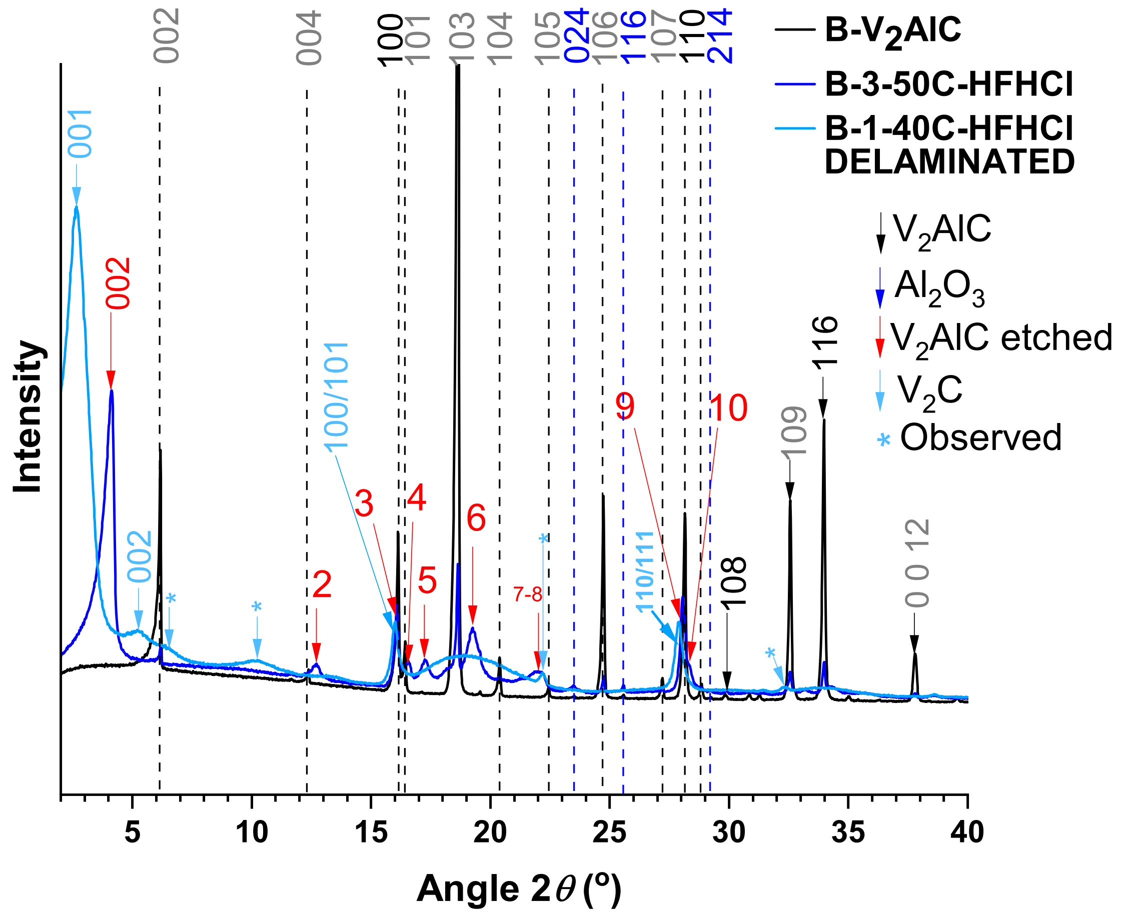

Figure S14: XRD patterns of the etched B-3-50C-HFHCl sample and the B-1-40C-HFHClDELAMINATED delaminated sample.

S14. X-Ray photoelectron spectroscopy studies of A-V_2AlC and B-V_2AlC precursors

The V 2p and O 1s spectra of the precursors A-V_2AlC and B-V_2AlC were studied.

The XPS data was acquired using a pass energy of 50 eV, energy step of 0.1 eV, acquisition time of 50 ms x 7 measurements. The proposed XPS model considered a Shirley type background, finite Lorentzian (LF) spectral line shapes [9]. Calibration was done with the O 1s component of the vanadium oxides and set at 530 eV [10].

The XPS models and fitting to the V 2p and O 1s spectra are shown in Figure S15. The corresponding parameters of the models are summarized in Tables S7 and S8. A first V $2p_{3/2}$ component at binding energy (BE) 512.53 eV was assigned to the V-C environment of the vanadium carbide. This spectral component is presented on delaminated materials, which confirmed their V-C nature. A second component (i) at 518.64 eV was identified. This component was first identified in sample B-V_2AlC where the BE range from 511 eV to 514 eV could not be modeled with a single component. Therefore, a second component was acknowledged and used also to model the spectrum of sample A-V_2AlC. The nature of this component was not identified with certainty. It could describe V(+1)/V(+2) oxides [10] or it could describe a different V-C chemical environment. In the V_2AlC, there are different atomic environments for V-C bonds having at least two different V-C bond lengths. So, this second spectrum component might be related to it. V-Al chemical environment is discarded as this is deemed to have a lower BE than the V-C bonds. Then a series of components describing vanadium oxides were identified. First, a component at 515.93 eV, 516 eV which is an oxide where vanadium has valence state slightly higher than +4, tentatively + 4.3 (V_6O_{13}) [11, 10], second, a component at 517.07 eV, 517.19 eV, which was assigned to V_2O_5 [10].

Shake-up satellite signals are pervasive in vanadium oxides [12]. In these samples a spectral signal at the 526 eV-528 eV BE range was identified with the presence of a shake-up satellite of the $2p_{3/2}$ transition. This satellite signal is known to be present in VO_2 and V_2O_3 oxides [12]. Here, this satellite was modeled with a component at 526.53 eV, 526.91 eV.

The O 1s spectrum was modeled with 3 components. A first one at 530 eV was identified as the one describing the vanadium oxides. A second component (ii) at 531.59 eV was identified. This component has been found in vanadium oxides, and was identified either with defective oxides or the presence of hydroxides [10]. Alternatively, it has been proposed as to be originated from -OH groups adsorbed onto transition metals [13]. Finally, a third component (iii) at 533.12 eV, 533.27 eV could be adsorbed water [13] and/or have a contribution of another satellite of the vanadium oxides present on the sample [12].

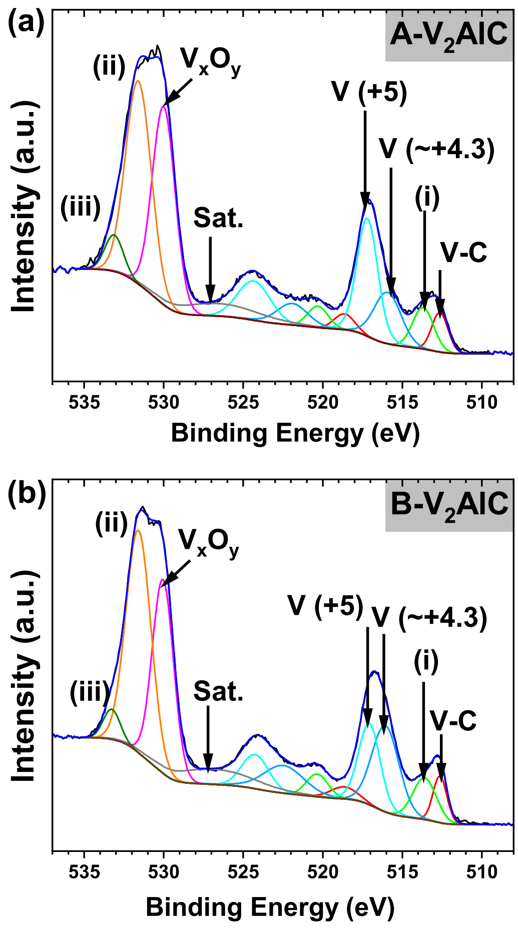

Figure S15: XPS spectra of the V 2p and O 1s transitions of (a) A-V_2AlC and (b) B-V_2AlC samples.

**Component**	**Transition**	**BE (eV)**	**FWHM (eV)**	**Δ2p (eV)**	**% at.conc.**
V-C	$2p_{3/2}$	512.63	1.2	6.01	1.53
V-C	$2p_{1/2}$	518.64	1.5		0.76
(i)	$2p_{3/2}$	513.69	1.55	6.62	2.09
(i)	$2p_{1/2}$	520.31	1.52		1.04
V_6O_{13}	$2p_{3/2}$	515.93	2.0	5.98	3.34
V_6O_{13}	$2p_{1/2}$	521.91	2.27		1.67
V_2O_5	$2p_{3/2}$	517.19	1.54	7.15	6.16
V_2O_5	$2p_{1/2}$	524.34	2.44		3.08
Sat. V_xO_y		526.53	6.16		-
V_xO_y	O 1s	530.0	1.69		34.93
(ii)	O 1s	531.59	1.9		40.26
(iii)	O 1s	533.12	1.35		5.14

Table S7: Parameters of the model used to fit the V 2p and O 1s XPS spectra of the A-V_2AlC sample

**Component**	**Transition**	**BE (eV)**	**FWHM (eV)**	**Δ2p (eV)**	**% at.conc.**
V-C	$2p_{3/2}$	512.63	1.03	6.03	1.55
V-C	$2p_{1/2}$	518.66	2.0		0.78
(i)	$2p_{3/2}$	513.60	1.73	6.75	2.34
(i)	$2p_{1/2}$	520.35	1.59		1.17
V_6O_{13}	$2p_{3/2}$	516.06	2.01	6.41	5.65
V_6O_{13}	$2p_{1/2}$	522.47	2.97		2.83
V_2O_5	$2p_{3/2}$	517.07	1.60	7.18	4.42
V_2O_5	$2p_{1/2}$	524.25	2.00		2.21
Sat. V_xO_y		526.91	6.09		-
V_xO_y	O 1s	530.0	1.57		31.92
(ii)	O 1s	531.58	1.86		43.23
(iii)	O 1s	533.27	1.24		3.91

Table S8: Parameters of the model used to fit the V 2p and O 1s XPS spectra of the B-V_2AlC sample

S15. X-Ray photoelectron spectroscopy studies of etched samples, sets A and B

The etched samples of set A and set B were analysed by XPS. The XPS data was acquired using a pass energy of 50 eV, energy step of 0.1 eV, acquisition time of 50 ms x 7 measurements.

The V 2p and O 1s transitions were modeled. The components for the V 2p spectra we modeled by asymmetric Doniach-Sunjic-Shirley (DSS) line shapes [14]. The O 1s spectra were modeled using LF spectral line shapes [9]. A Shirley type background was used. The model was guided by a previous discussion on models of $Ti_3C_2T_x$ [15]. The full width half maximum (FWHM) of V $2p_{3/2}$ components were set to be equal. The same applies for V $2p_{1/2}$. The area ratio of V $2p_{3/2}$/V $2p_{1/2}$ was set to be 2:1. The ΔV 2p of related components was not constrained. Calibration was done setting the components assigned to C-V-O to 529 eV. This calibration was found to be equivalent to calibrating with C 1s of C-V to 281.88 eV [13], which was used also for the analysis of the samples of set D (Section S16).

The components were assigned according to previous reports [15] and our own analysis considering samples synthesized at various conditions (see the main text). The model parameters are summarized in Table S9 for the sample A-1-35C-HF and Table S10 for the sample B-1-40C-HFHCl, as representative data of each set. Other samples were analysed using the same model and consistency across samples was confirmed (Figures S16, S17).

First, several V $2p_{3/2}$, V $2p_{1/2}$ components were identified. The first 4 were assigned to chemical functionalities attached to V. The assignation was according a previous model proposed for $Ti_3C_2T_x$ synthesized using the same method and similar conditions than used here [15]. Namely, components were assigned, from low to high BE order, to C-V-O/O/O, C-V-F/O/O, C-V-F/F/O and C-V-F/F/F chemical functionalities (Table S9), which considers bonds to atoms of increasing electronegativity in several proportions [15]. The origin of these spectral components on the stated chemical functionalities has been validated by some authors using temperature programmed XPS and scanning transmission electron microscopy (STEM) [16, 13]. Second, a spectral component due to the presence of vanadium oxides was considered. As largely discussed in this work, V_2C is very prone to oxidation. The line shape to account for vanadium oxides was derived from the vanadium oxide components identified in the corresponding V_2AlC precursor (Section S14). The method to derive such line shape is largely discussed in previous work by Mendoza-Sánchez et al. [17]. The resulting model was consistent across samples (Figures S16, S17).

The O 1s transition considered 4 components. The first component at 529.0 eV was assigned to the C-V-O bonds. The second component at 530.23 eV was assigned to the vanadium oxides. Since this component was at 530.0 eV for vanadium oxides in the V_2AlC , the slight shift to higher BE for the etched samples can be attributed to the incorporation of F atoms, as in the case of the oxides in $Ti_3C_2T_x$ [15]. So, this component was considered to correspond to $V_xO_yF_z$ species. Since the BE shift is very small, then it is considered that the number of incorporated F atoms is low. A third component at (j) 531.23 eV seems to be at a similar position than the component (ii) in the corresponding model of the V_2AlC precursor, and thus, according to the previous discussion, could be related to defective vanadium oxides or hydroxides or be due to the presence of adsorbed -OH groups onto the V. A fourth component (jj) at 532.29 eV is present in the etched samples but not on the precursor V_2AlC, thus, it should be associated to chemical groups arising from the etching reactions or secondary reactions. According to previous findings, this component might correspond to C-V-OH and/or C-V-O/F bonds [15]. However, as discussed in the

main text, these (j) and (jj) components remain after etching procedures at high temperatures, which rids vanadium off, and according to XRD studies, forms Al_2O_3. This indicated that these spectral components describe chemical groups involving Al, instead of V, namely, $Al(OH)_3$ for the (j) component and Al_2O_3 for the (jj) component. Finally, a component at 533.09 eV was assigned to adsorbed water.

This is the best model we could propose based on guidance of previous findings and experimental data and statistics here obtained. The model certainly needs a more in-depth description and discussion, but this not the objective of this work. Here, the the focus is rather to use XPS to elucidate key aspects of the chemistry of etching reactions (main text). A full discussion of XPS models for etched and delaminated V-based MXenes will be reported in a separate work.

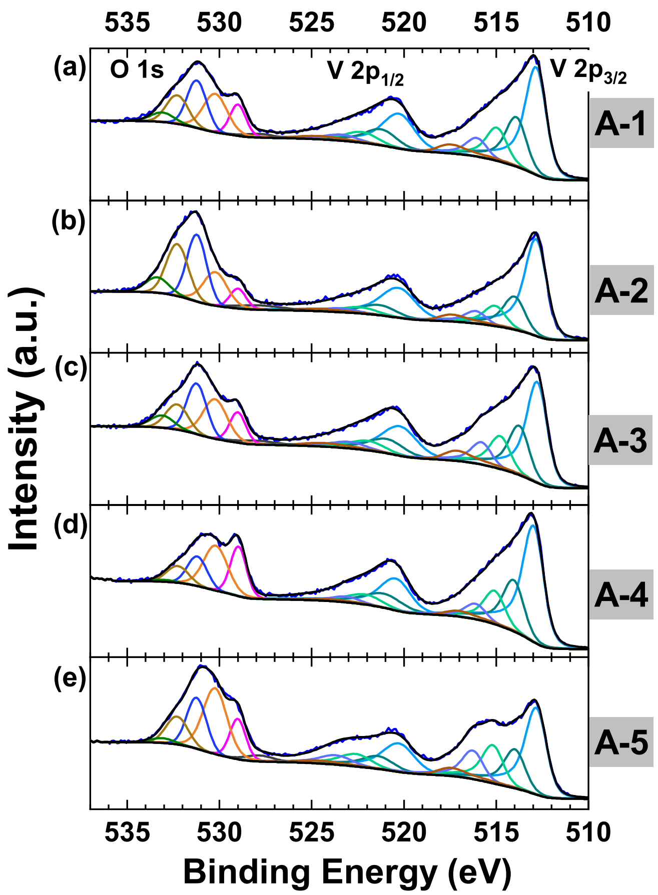

Figure S16: XPS spectra of the V 2p and O 1s transitions of samples of set A: (a) A-1-35C-HF, (b) A-2-45C-HF, (c) A-3-55C-HF, (d) A-4-45C-HFHCl and (e) A-5-45C-HF-100rpm.

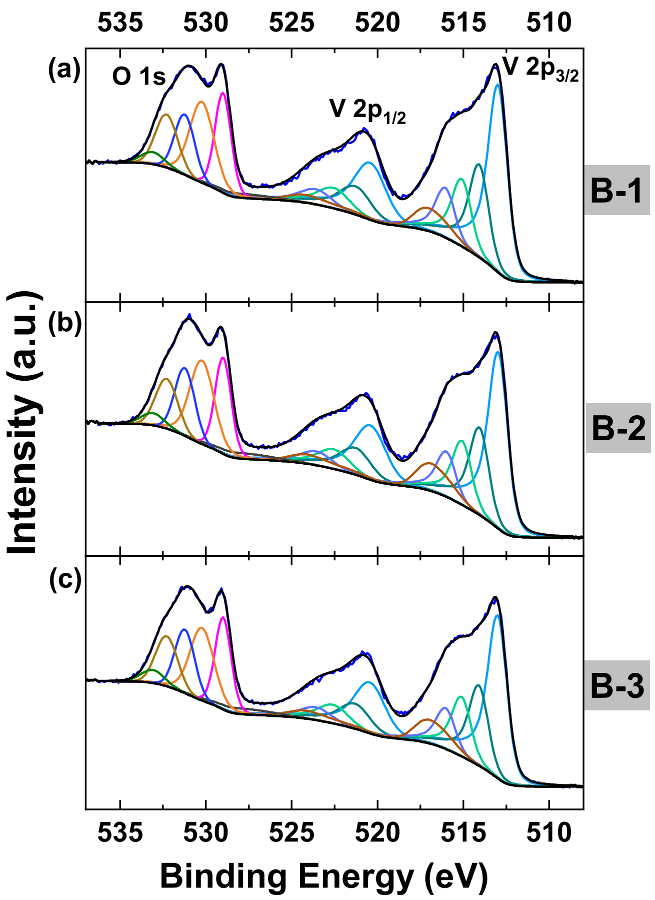

Figure S17: XPS spectra of the V 2p and O 1s transitions of samples of set B: (a) B-1-40C-HFHCl, (b) B-2-40C-HFHCl and (c) B-3-50C-HFHCl.

Transition	Assignation	BE (eV)	FWHM (eV)	Δ2p (eV)	% at.conc.
V $2p_{3/2}$	C-V-O/O/O	512.79	1.3	7.4	14
V $2p_{1/2}$	C-V-O/O/O	520.19	2.01		7
V $2p_{3/2}$	C-V-F/O/O	513.87	1.3	7.3	6.45
V $2p_{1/2}$	C-V-F/O/O	521.17	2.01		3.23
V $2p_{3/2}$	C-V-F/F/O	514.92	1.3	7.3	4.31
V $2p_{1/2}$	C-V-F/F/O	522.22	2.01		2.15
V $2p_{3/2}$	C-V-F/F/F	516.01	1.3	7.3	2.43
V $2p_{1/2}$	C-V-F/F/F	523.31	2.01		1.21
V 2p	$V_xO_yF_z$	517.47	1		7.69
V 2p	V $2p_{3/2}$ sat.	527.89	1.9		–
O 1s	C-V-O	529.00	1.02		8.01
O 1s	$V_xO_yF_z$	530.23	1.53		15.5
O 1s	(j)	531.23	1.25		14.59
O 1s	(jj)	532.29	1.39		9.74
O 1s	H_2O ads.	533.09	1.46		3.69

Table S9: Parameters of the model used to fit the V 2p and O 1s XPS spectra of the A-1-35C-HF sample

**Transition**	**Assignation**	**BE (eV)**	**FWHM (eV)**	**Δ2p (eV)**	**% at.conc.**
V $2p_{3/2}$	C-V-O/O/O	512.93	1.3	7.41	11.85
V $2p_{1/2}$	C-V-O/O/O	520.34	2.13		5.93
V $2p_{3/2}$	C-V-F/O/O	514.03	1.3	7.18	5.87
V $2p_{1/2}$	C-V-F/O/O	521.21	2.13		2.94
V $2p_{3/2}$	C-V-F/F/O	515.04	1.3	7.44	4.26
V $2p_{1/2}$	C-V-F/F/O	522.48	2.13		2.13
V $2p_{3/2}$	C-V-F/F/F	516.01	1.3	7.52	3.15
V $2p_{1/2}$	C-V-F/F/F	523.52	2.13		1.58
V 2p	$V_xO_yF_z$	517.05	1		9.5
V 2p	V $2p_{3/2}$ sat.	527.89	4.0		–
O 1s	C-V-O	529.00	1.11		13.57
O 1s	$V_xO_yF_z$	530.23	1.67		17.32
O 1s	(j)	531.23	1.36		9.81
O 1s	(jj)	532.29	1.52		9.63
O 1s	H_2O ads.	533.1	1.6		2.46

Table S10: Parameters of the model used to fit the V 2p and O 1s XPS spectra of the B-1-40C-HFHCl sample

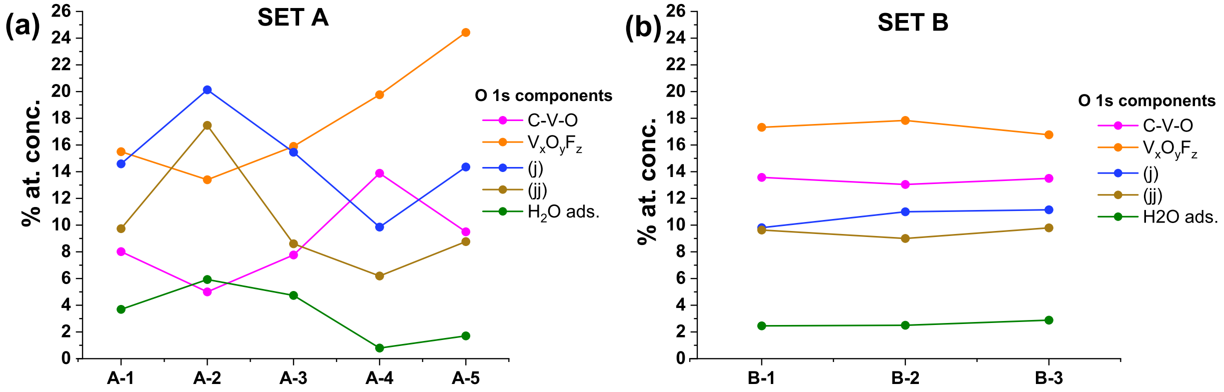

Figure S18: Quantification of the O 1s components of samples of (a) set A (A-1-35C-HF, A-2-45C-HF, A-3-55C-HF, A-4-45C-HFHCl and A-5-45C-HF-100rpm) and (b) (B-1-40C-HFHCl, B-2-40C-HFHCl and B-3-50C-HFHCl).

S16. X-Ray photoelectron spectroscopy studies of samples of set D

A set of samples D was specifically analysed by XPS, in order to find the necessary contrast to identify the chemical species involved in $V_xAl_yC_z$ etched samples and V_2CT_x delaminated samples. The synthesis conditions for this set of samples are described in Table 1 (main text) and reproduced again here for the sake of clarity (Table S11).

**Set D, D-V_2AlC (1 g)**								
**Sample**	**Acid (ml)**	**Vessel size**	**T (°C)**	**t (d)**	**MR (rpm)**	**Yield (%)**	**Delam.**	**Deg. etch**
D-1-35C-HF	HF(20)	S	35	4	400	80.84	yes	well
D-2-40C-HF	HF(20)	L	40	4	400	64.1	-	early over
D-3-50C-HF	HF(20)	L	50	4	400	31.9	-	over

Table S11: Synthesis conditions for experimental set D used for XPS analysis. T= temperature, t = time, L = Large, S= small, MR = Mixing rate. The notes well = well etched, early over = early overetched and over = overetched make reference to the degree of etching of the sample. HF is 48 wt. %. Only the sample D-1-35C-HF underwent delamination to render the D-1-35C-HF-DEL sample. This sample was delaminated in TBAOH 10 wt.% and a 1.5:1 TBAOH: V_2C mol ratio, for 16 h at 35 °C and mixing rate of 350 rpm and in a small vessel. The note "yes" makes reference to whether delaminated product was obtained.

The XPS data was acquired using a pass energy of 20 eV, energy step of 0.1 eV, and acquisition times from 2,000 to 40,000 ms/step as required for particular spectral regions.

The V $2p_{3/2}$, V $2p_{1/2}$ spectra were modelled just as for sets A and B (Section S15). The same applies for the O 1s spectral region, which considered the very same components than in sets A and B. However, the origin of components (j) and (jj) was elucidated using key information provided by XPS data of set D, as described in the main text (Section 3.10). The model data is reported for the D-1-35C-HF sample (Table S12)

The C 1s spectrum was modeled using LF spectral line shapes [9] and a Shirley-type background. The spectral components identified are reported in Table S13. A component at the lowest BE was assigned to the V-C bonds and was used for calibration, set at 281.88 eV [13]. A second component was identified at BE 284.23-284.4 eV and was assigned to C-C sp^2 bonds [13]. Other components were assigned as described in Table S13 based on the literature [18]. The origin of these species is attributed to hydrocarbon compounds present as contamination (adventitious carbon) and, in the case of CF_2 groups, contamination from the PTFE of the reactions vessel and stirring bar. CF_2 groups were also identified with the F 1s spectral region.

The F 1s spectra were modeled using LF spectral lines and a Shirley-type background. Four components were identified and assigned as described in Table S14. The BE of the component assigned to V-F bonds was slightly lower for the samples D-2-40C-HF and D-3-50C-HF by 0.25-0.32 eV (Figure 11i-l). This could be attributed to charging effects or to different degrees of -F functionalisation. The same can be said about the component assigned to the $V_xO_yF_z$ component, which also showed slight shifts.

The Al 2s spectra were modeled using Voigt (LA) line shapes [14] and a linear background (Figure S19a-c). A plausible model was proposed considering 3 components (Table S15). The component at the lowest BE of 117.04 eV was assigned to the V-Al component, which as per the etching procedures is expected to have a low intensity. This component was present in the precursor V_2AlC at 117.23 eV. The other components at higher binding energies were assigned to $Al(OH)_3$ and Al_2O_3 according to the literature [19].

The Al 2p spectra were modeled using LF shapes and a Shirley-type background (Figure S19d-g, Table S16). These spectra are complex due to the overlap with the V 3s signal. Here, 4 components were proposed for the V 3s part of the spectra, corresponding to the 4 components revealed by the V 2p spectra. A component do to the vanadium oxides is not included as it could not be fitted. Data improvement will be necessary for an improved modelling. These components were, thus, not discussed. The Al 2p part of the spectra showed clearly two components. Here, again, because of the signal overlap the model was not stable and those two components had shifts that cannot be attributed to changes in chemical environment. The first component at ca. 71-72 eV was assigned to the V-Al bond, and the second component at ca. 73.93-74.20 was assigned to contributions from Al-based compounds, Al_2O_3 and/or $Al(OH)_3$. In the strict sense, the Al 2p spectra of a chemical species should have two components corresponding to the spin-orbit splitting. However, the current data does not allow for adequate modelling. Nevertheless, the assignation of the high BE components is certain as confirmed by contrast across samples. The spectrum for the sample D-3-50C-HF (Figure S19f) clearly showed a comparatively high intensity of the component at 74.37 eV as compared to the one at 71.61 eV (Figure S20e), which clearly demonstrates that the latter described the V-Al bonds, almost gone in this overetched sample, whereas the former described Al-based impurities, becoming significant in this overetched sample.

Transition	Assignation	BE (eV)	FWHM (eV)	Δ2p (eV)	% at.conc.
V $2p_{3/2}$	C-V-O/O/O	512.97	1.13	7.48	11.46
V $2p_{1/2}$	C-V-O/O/O	520.45	1.95		5.73
V $2p_{3/2}$	C-V-F/O/O	513.98	1.13	6.93	5.8
V $2p_{1/2}$	C-V-F/O/O	520.91	1.95		2.9
V $2p_{3/2}$	C-V-F/F/O	515.03	1.13	7.31	4.09
V $2p_{1/2}$	C-V-F/F/O	522.34	1.95		2.05
V $2p_{3/2}$	C-V-F/F/F	516.03	1.13	7.3	2.19
V $2p_{1/2}$	C-V-F/F/F	523.33	1.95		1.1
V 2p	$V_xO_yF_z$	514.26	1		21.01
V 2p	V $2p_{3/2}$ sat.	527.05	4.0		–
O 1s	C-V-O	529.05	1.02		12.1
O 1s	$V_xO_yF_z$	530.28	1.53		11.32
O 1s	(j)	531.28	1.25		7.3
O 1s	(jj)	532.34	1.39		9.00
O 1s	H_2O ads.	533.15	1.46		3.96

Table S12: Parameters of the model used to fit the V 2p and O 1s XPS spectra of the D-1-35C-HF sample.

C 1s	D-1		D-2		D-3		D-1-DEL	
Assignation	BE (eV)	FWHM (eV)	BE (eV)	FWHM (eV)	BE (eV)	FWHM (eV)	BE (eV)	FWHM (eV)
V-C	281.88	0.85	281.88	0.79	-	-	281.88	0.89
C-C	284.23	1.75	284.23	1.62	284.23	1.55	284.41	1.61
C*-C=O	-	-	-	-	285.59	1.38	285.81	1.38
−CO	286.11	1.38	286.12	1.29	-	-	-	-
C(=O)OC*	-	-	-	-	287.09	1.65	287.25	1.79
OCO	287.73	1.9	287.75	1.73	-	-	-	-
CF_2	292.04	1.28	-	-	291.89	1.2	-	-

Table S13: Parameters of XPS models for C 1s spectra of samples of set D (D-1-35C-HF, D-2-40C-HFHCl, D-3-50C-HFHCl and D-1-35C-HF-DEL).

**F 1s**	**D-1**		**D-2**		**D-3**		**D-1-DEL**	
**Assignation**	**BE (eV)**	**FWHM (eV)**	**BE (eV)**	**FWHM (eV)**	**BE (eV)**	**FWHM (eV)**	**BE (eV)**	**FWHM (eV)**
V-F	683.89	1.19	683.57	1.19	683.64	1.1	683.84	1.17
$V_xO_yF_z$	684.61	1.96	684.32	1.47	684.36	1.81	684.54	1.92
$(CHFCH_2)_n$	686.23	1.93	686	1.93	685.99	1.78	686.04	1.89
$(CF_2\text{-}CF_2)$	689.28	1.67	689.02	1.92	689.16	1.71	688.17	2

Table S14: Parameters of XPS models for F 1s spectra of samples of set D (D-1-35C-HF, D-2-40C-HFHCl, D-3-50C-HFHCl and D-1-35C-HF-DEL).

**Al 2s**	**D-1**		**D-2**		**D-3**	
**Assignation**	**BE (eV)**	**FWHM (eV)**	**BE (eV)**	**FWHM (eV)**	**BE (eV)**	**FWHM (eV)**
V-Al	117.04	1.02	117.04	1.17	117.08	1.15
$Al_2(OH)_3$	119.15	2.2	118.85	1.96	119.19	2.03
Al_2O_3	120.88	2.2	120.9	1.96	120.92	2.03

Table S15: Parameters of XPS models for Al 2s spectra of samples of set D (D-1-35C-HF, D-2-40C-HFHCl and D-3-50C-HFHCl).

**Al 2p**	**D-1**		**D-2**		**D-3**		**D-1-DEL**	
**Assignation**	**BE (eV)**	**FWHM (eV)**	**BE (eV)**	**FWHM (eV)**	**BE (eV)**	**FWHM (eV)**	**BE (eV)**	**FWHM (eV)**
V 3s 1	64.15	3	64.15	2.94			64.62	3
V 3s 2	65.86	1.82	65.87	1.79	66.21	1.88	66.34	1.82
V 3s 3	67.32	2.16	67.32	2.12	67.92	1.87	67.79	2.16
V 3s 4	69.09	2.25	69.09	2.21	69.38	2.06	69.56	2.25
V-Al	71.18	2.01	71.4	1.83	71.61	0.67	71.87	1.86
$Al_2(OH)_3/Al_2O_3$	74.2	1.9	73.93	1.58	74.37	1.66	74.4	1.61

Table S16: Parameters of XPS models for Al 2p spectra of samples of set D (D-1-35C-HF, D-2-40C-HFHCl, D-3-50C-HFHCl and D-1-35C-HF-DEL).

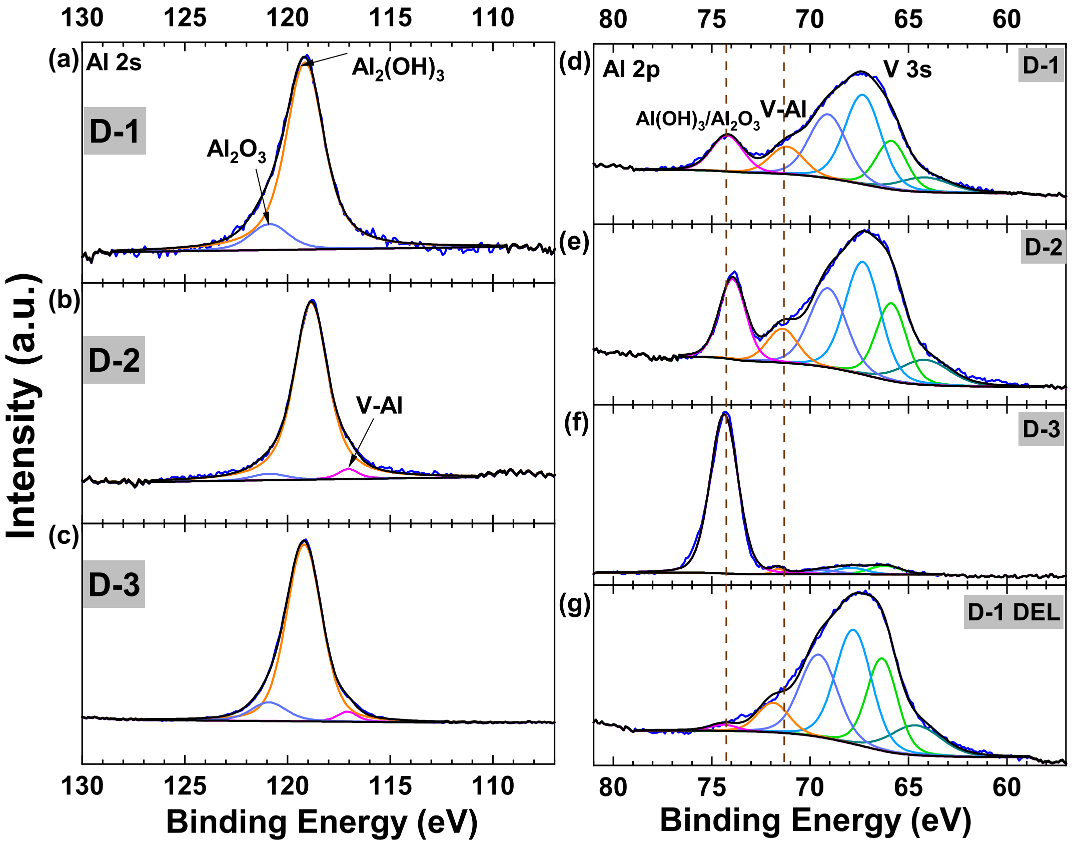

Figure S19: XPS spectra of samples of set D, top to bottom row: D-1-35C-HF, D-2-40C-HFHCl, D-3-50C-HFHCl and D-1-35C-HF-DEL (Table S11), (a)-(c) Al 2s, (d)-(g) V 3s and Al 2p spectral regions.

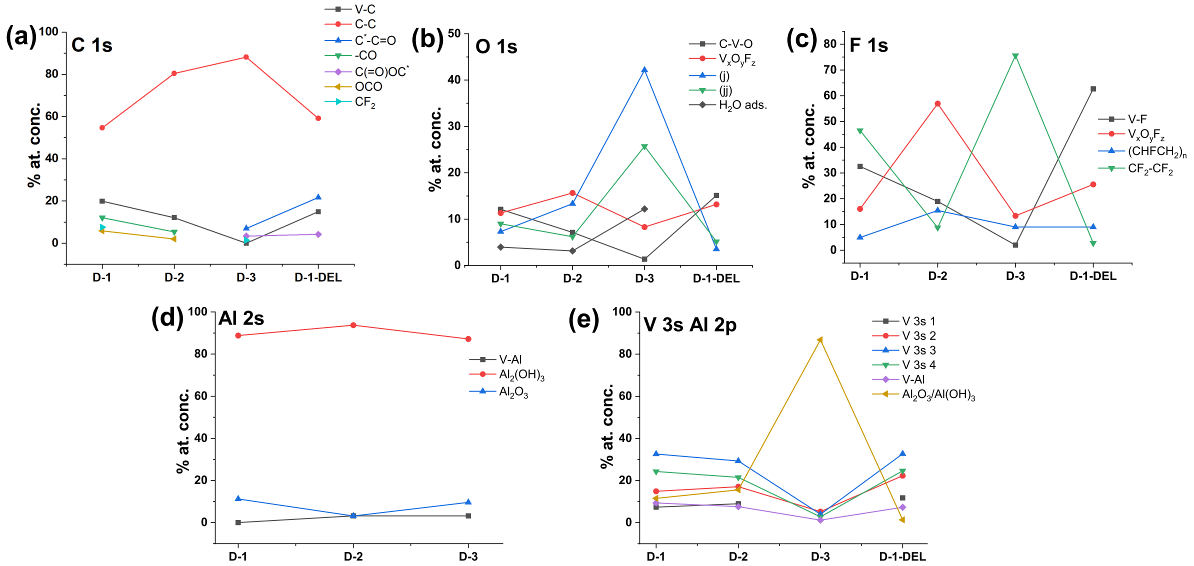

Figure S20: Quantification of chemical species for samples of set D (D-1-35C-HF, D-2-40C-HFHCl, D-3-50C-HFHCl and D-1-35C-HF-DEL) according to spectral components of several spectral regions: (a) C 1s, (b) O 1s, (c) F 1s, (d) Al 2s and (e) V 3s Al 2p.

References

[1] Rodriguez-Carvajal, J. FULLPROF: A Program for Rietveld Refinement and Pattern Matching Analysis. *Abstracts of the Satellite Meeting on Powder Diffraction of the XV Congress of the IUCr* **1990**, 127.

[2] Finger, L. W.; Cox, D. E.; Jephcoat, A. P. A Correction for Powder Diffraction Peak Asymmetry Due to Axial Divergence. *J. Appl. Crystallogr.* **1994**, *27*, 892–900.

[3] Shan, Q.; Mu, X.; Alhabeb, M.; Shuck, C. E.; Pang, D.; Zhao, X.; Chu, X.-F.; Wei, Y.; Du, F.; Chen, G.; Gogotsi, Y.; Gao, Y.; Dall'Agnese, Y. Two-Dimensional Vanadium Carbide (V_2C) MXene as Electrode for Supercapacitors With Aqueous Electrolytes. *Electrochem. Commun.* **2018**, *96*, 103–107.

[4] Hu, C.; Zhang, J.; Wang, J.; Li, F.; Wang, J.; Zhou, Y. Crystal Structure of V_4AlC_3: A New Layered Ternary Carbide. *J. Am. Ceram. Soc.* **2008**, *91*, 636–639.

[5] Bérar, J.-F.; Lelann, P. E.S.D.'s and Estimated Probable Error Obtained in Rietveld Refinements with Local Correlations. *J. Appl. Crystallogr.* **1991**, *24*, 1–5.

[6] Werner, P.-E.; Eriksson, L.; Westdahl, M. TREOR, a Semi-exhaustive Trial-And-Error Powder Indexing Program for All Symmetries. *J. Appl. Crystallogr.* **1985**, *18*, 367–370.

[7] Liang, K. S.; Chianelli, R. R.; Chien, F. Z.; Moss, S. C. Structure of Poorly Crystalline MoS_2 -A Modeling Study. *J. Non-Cryst. Solids* **1986**, *79*, 251–273.

[8] Shekhirev, M.; Shuck, C. E.; Sarycheva, A.; Gogotsi, Y. Characterization of MXenes at Every Step, From Their Precursors to Single Flakes and Assembled Films. *Prog. Mater Sci.* **2021**, *120*, 100757.

[9] Major, G. H.; Shah, D.; Avval, T. G.; Fernandez, V.; Fairley, N.; Linford, M. R. Advanced Line Shapes in X-Ray Photoelectron Spectroscopy II. The Finite Lorentzian (LF) Line Shape (with some MATLAB code illustrating the use of the subplot function). *Vacuum Technology and coating* **2020**, 35–39.

[10] Biesinger, M. C.; Lau, L. W. M.; Gerson, A. R.; Smart, R. S. C. Resolving Surface Chemical States in XPS Analysis of First Row Transition Metals, Oxides and Hydroxides: Sc, Ti, V, Cu and Zn. *Appl. Surf. Sci.* **2010**, *257*, 887–898.

[11] Mendialdua, J.; Casanova, R.; Barbaux, Y. XPS Studies of V_2O_5, V_6O_{13}, VO_2 and V_2O_3. *J. Electron. Spectrosc. Relat. Phenom.* **1995**, *71*, 249–261.

[12] Zimmermann, R.; Steiner, P.; Claessen, R.; Reinert, F.; Hüfner, S.; Blaha, P.; Dufek, P. Electronic Structure of 3d-Transition-Metal Oxides: On-Site Coulomb Repulsion Versus Covalency. *J. Phys.: Condens. Matter.* **1999**, *11*, 1657–1682.

[13] Näslund, L.-; Persson, P. O. ; Rosen, J. X-Ray Photoelectron Spectroscopy of Ti_3AlC_2, $Ti_3C_2T_z$, and TiC Provides Evidence for the Electrostatic Interaction between Laminated Layers in MAX-Phase Materials. *J. Phys. Chem. C* **2020**, *124*, 27732–27742.

[14] Moeini, B.; Linford, M. R.; Fairley, N.; Barlow, A.; Cumpson, P.; Morgan, D.; Fernandez, V.; Baltrusaitis, J. Definition of a New (Doniach-Sunjic-Shirley) Peak Shape for Fitting Asymmetric Signals Applied to Reduced Graphene Oxide/Graphene Oxide XPS Spectra. *Surf. Interface Anal.* **2022**, *54*, 67–77.

[15] Natu, V.; Benchakar, M.; Canaff, C.; Habrioux, A.; Célérier, S.; Barsoum, M. W. A Critical Analysis of the X-Ray Photoelectron Spectra of $Ti_3C_2T_z$ MXenes. *Matter* **2021**, *4*, 1224–1251.

[16] Persson, I.; Näslund, L.-; Halim, J.; Barsoum, M. W.; Darakchieva, V.; Palisaitis, J.; Rosen, J.; Persson, P. O. On the Organization and Thermal Behavior of Functional Groups On Ti_3C_2 MXene Surfaces in Vacuum. *2D Materials* **2018**, *5*, 015002.

[17] Baltrusaitis, J.; Mendoza-Sanchez, B.; Fernandez, V.; Veenstra, R.; Dukstiene, N.; Roberts, A.; Fairley, N. Generalized Molybdenum Oxide Surface Chemical State XPS Determination via Informed Amorphous Sample Model. *Appl. Surf. Sci.* **2015**, *326*, 151–161.

[18] Beamson, G.; Briggs, D. High Resolution XPS of Organic Polymers: The Scienta ESCA300 Database. *J. Chem. Educ.* **1993**, *70*, A25.

[19] National Institute of Standards and Technology (NIST). 2012; https://srdata.nist.gov/xps/Default.aspx.
